# Chiral Recognition of Amino Acid Esters in Organic Solvents Using a Glucose-Based Receptor

**DOI:** 10.3390/molecules27072177

**Published:** 2022-03-28

**Authors:** Leah Susanne Mönkemöller, Martin Schnurr, Bartosz Lewandowski

**Affiliations:** Laboratory of Organic Chemistry, ETH Zurich, Vladimir-Prelog-Weg 1-5, 8093 Zurich, Switzerland; mleah@student.ethz.ch (L.S.M.); martin.schnurr@org.chem.ethz.ch (M.S.)

**Keywords:** amino acids, chiral recognition, carbohydrates, crown ethers, host:guest chemistry

## Abstract

Due to the chemical and biological relevance of amino acids, efficient methods for the recognition and separation of their enantiomers are highly sought after. Chiral receptors based on extended molecular scaffolds are typically employed for this purpose. These receptors are often effective only in specific environments and towards a narrow scope of amino acid guests. Recently we reported a simple, glucose-based macrocycle capable of enantioselective binding of a broad range of amino acid methyl esters in water. Herein we demonstrate that the same receptor can be used for chiral recognition of amino acid esters in organic solvents. We show that the binding affinity and selectivity of the receptor are highly dependent on the coordinating strength of the solvent. An in-depth analysis of the receptor’s conformation and its interactions with amino acid methyl esters allowed us to propose a binding mode of amino acids to the receptor in CDCl_3_. The binding modes in CDCl_3_ and D_2_O were then compared, highlighting the main interactions responsible for binding affinity and selectivity in each solvent. We envision that the insight provided by this study will facilitate the development of further amino acid receptors based on monosaccharides with improved binding affinities and both enantio- as well as chemoselectivities.

## 1. Introduction

Amino acids are one of the most important classes of biomolecules. They are applied for the purposes of asymmetric synthesis [1], catalysis [2,3], chemical biology [4] and materials science [5,6]. In nature, apart from being the constituents of proteins, amino acids play crucial roles in numerous biological processes [7]. The chemical and biological properties of amino acids often depend on their absolute configuration [8]. For instance, *l*-serine is a precursor of phosphatidylserine and sphingolipids, which are constituents of the plasma membrane and are involved in cell cycle signaling [9]. Its enantiomer, *d*-serine, serves as a ligand regulating the activity of N-methyl-*d*-aspartate–type glutamate receptor (NMDAR), and plays a key role in brain development and learning [10]. The racemization of *l*- to *d*-aspartic acid, which occurs at a known rate in mammalian tooth enamel [11], can be used to determine the age of living mammals and to date unidentified samples in forensic science [12]. The selective binding of amino acid enantiomers is therefore highly valuable for diagnostic, therapeutic and technological purposes [13,14,15].

**Figure 1:**
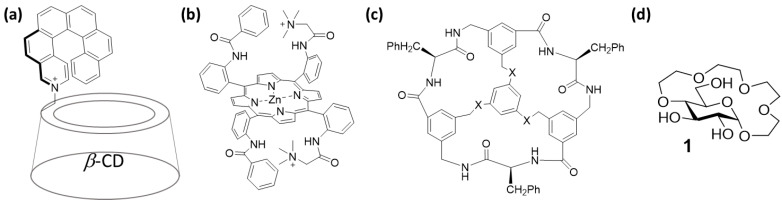
Examples of enantioselective receptors of amino acids based on: (**a**) cyclodextrins [19], (**b**) porphyrins [21], (**c**) and cyclophanes [22]. (**d**) Glucose-based receptor **1** for enantioselective binding of amino acid methyl esters in water [23].

Enantioselective binding/separation of amino acids has been achieved with synthetic molecular receptors, typically based on extended scaffolds (e.g., resorcin- and calixarenes [16,17], cyclodextrins (Figure 1a) [18,19], porphyrins (Figure 1b) [20,21] or cyclophanes (Figure 1c) [22]). Despite the breadth of available receptors, most of them are only effective in a very specific environment and display binding selectivity towards a limited number of amino acids. Recently, we reported a simple glucose-based crown ether (**1**) capable of enantioselective binding of a broad range of amino acid methyl esters in water (Figure 1d) [23]. The enantioselectivities displayed by receptor **1** towards amino acids with hydrophobic side-chains were among the highest reported for small molecule synthetic receptors in aqueous media to date [24,25]. Herein, we demonstrate that the same receptor is also capable of binding amino acid methyl esters in several organic solvents displaying similar levels of enantioselectivity and often greatly increased binding affinity. Through conformational studies and computational modelling, we provide insight into the binding mode of the receptor and the origins of enantioselectivity in CDCl_3_.

## 2. Results and Discussion

### 2.1. Experiment Design

Receptor **1** was prepared following a previously reported 6-step procedure for phenyl-*β*-*d*-glucopyranoside [23]. **1** is soluble in the majority of common organic solvents, apart from the highly apolar ones, such as hexane or toluene. This gives an opportunity to study the binding of amino acid-derived guests by **1** in organic media. We chose to investigate amino acid methyl ester hydrochlorides as guests to compare the results of the binding studies in organic media to those obtained earlier in water [23]. Furthermore, salts of amino-acid methyl esters are reasonably well soluble in several organic solvents (which is not the case for free, zwitterionic amino acids). In order to validate how solvent polarity and coordinating ability [26] affect the host-guest interactions between **1** and amino acid methyl ester hydrochlorides, we selected DMSO, acetonitrile and chloroform for our studies. Incidentally, these are also solvents that can dissolve the majority of amino acid methyl ester hydrochlorides.

**Figure 2:**
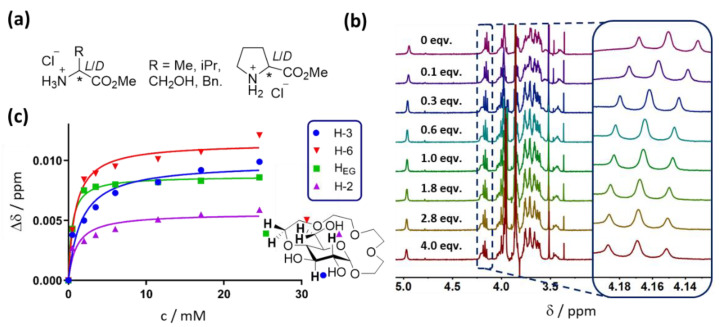
(**a**) Structures of amino acid guests used in this study. (**b**) ^1^H NMR spectra recorded during the titration of H-*l*-Val-OMe to **1** in CDCl_3_ upon addition of the indicated number of equivalents of guest (inset shows the shifting of H-3 glucopyranose protons in **1** during the titration)—shown as a representative example. (**c**) Titration curves obtained from the ^1^H NMR data shown in (**b**).

For the binding studies, we selected methyl ester salts derived from alanine, threonine, valine, phenylalanine and proline as guests (Figure 2a). We envisioned that this should allow us to verify how the size and character of the amino acid side chain (aromatic, hydrophobic or hydrophilic) influences the binding affinity and selectivity of receptor **1** in different solvents. Proline was chosen as the only example of a proteinogenic amino acid bearing a cyclic secondary amine. Furthermore, these five amino acids were bound by **1** in water with the highest (valine and phenylalanine), moderate (alanine and threonine) and lowest (proline) affinities [23].

### 2.2. Binding Studies

The binding affinities of receptor **1** to the amino acid methyl esters in DMSO-d_6_, CD_3_CN and CDCl_3_ were studied by titration experiments monitored by ^1^H NMR spectroscopy. In a typical experiment, a solution of an enantiomerically pure guest was added in portions to the solution of **1**, and a ^1^H NMR spectrum was recorded after each addition (see Figure 2b for an example). The changes in the chemical shifts of several receptor protons upon guest addition were followed (Figure 2c) and then fitted into the equation for single-site non-competitive binding. The results of the titration experiments are summarised in Table 1. For comparison, data previously obtained in D_2_O [23] are also presented.

In DMSO-d_6_, both the binding affinities as well as enantioselectivities of **1** towards investigated amino acid ester salts were considerably lower than in D_2_O, except for Pro, which was bound with similar affinity and selectivity. In CD_3_CN, the association constants were higher than in D_2_O with similar (in the case of Ala, Thr and Pro) or slightly lower selectivity (for Val and Phe). The measured association constants were the highest in CDCl_3_, with the strongest binding and highest selectivity observed for Val (albeit the selectivity was slightly lower than for the same amino acid in water). In general, the binding selectivities in CDCl_3_ were at a similar level to those measured in D_2_O, the only exception being Phe, bound with low enantioselectivity. The low enantioselectivity of Phe binding could be partly due to the fact that 10 vol% of DMSO-d_6_ had to be added to the guest solution for solubility reasons.

The binding studies in DMSO-d_6_, CD_3_CN and CDCl_3_ revealed a clear correlation between the coordinating strength of the solvent [26] and the association constants of complexes formed between the glucose-based crown ether and ammonium salts of amino acid methyl esters. As expected, the lowest binding affinities were observed in DMSO-d_6_, which is a highly coordinating polar solvent. The strong solvation of ammonium cations in DMSO-d_6_ results in their very weak binding to the macrocyclic receptor. The association constants of complexes measured in CD_3_CN were higher than in DMSO-d_6_ and D_2_O. CD_3_CN is less polar and has lower coordinating strength than both DMSO-d_6_ and D_2_O. Thus, it attenuates the interactions between the crown ether oxygens in **1** and the ammonium cation in the guest to a much lesser extent allowing for the formation of complexes with higher association constants. Since CDCl_3_ is the least polar and least coordinating of the studied solvents, the association constants of the complexes between **1** and amino-acid methyl ester salts measured in CDCl_3_ were the highest.

The obtained results were more ambiguous with regards to binding enantioselectivity. The only common features were the preference of receptor **1** towards *l*-enantiomers of amino acids observed for all guests and in all studied solvents, and that proline was bound with rather low enantioselectivity. The selectivity of threonine binding varied between solvents. It was low in DMSO-d_6_ and CD_3_CN and somewhat higher in CDCl_3_ and D_2_O. The selectivity of alanine binding was low in DMSO-d_6_, CD_3_CN and D_2_O and slightly increased in CDCl_3_. The enantioselectivity of valine binding was, in general, high; however, it was slightly lower in organic solvents compared to D_2_O. The largest variations in enantioselectivity were observed for the binding of phenylalanine. The selectivity in DMSO-d_6_ and CD_3_CN was relatively high but already considerably lower than in D_2_O. In CDCl_3_, however, the selectivity dropped all the way to 1.5:1, being the lowest value measured in this solvent among the five tested amino acids. In general, the largest variations in binding affinity and enantioselectivity were seen in CDCl_3_ compared to the other two solvents.

### 2.3. Conformational Studies and Binding Mode Elucidation in CDCl_3_

To gain further insight into the binding mode of amino acid methyl esters salts by receptor **1** in CDCl_3_, we performed additional NMR spectroscopic analyses and computational modelling.

#### 2.3.1. Conformational Analysis of Receptor **1**

**Figure 3:**
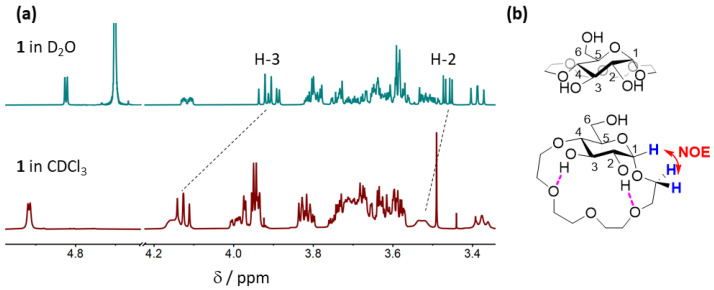
(**a**) ^1^H NMR spectra of **1** in D_2_O (top) and in CDCl_3_ (bottom). (**b**) Proposed conformation of **1** in D_2_O (top) and in CDCl_3_ (bottom)—H atoms involved in through space interactions are shown in blue, the interaction is indicated in red, and proposed intramolecular H-bonds are shown in purple.

First, we investigated the conformation of **1** in CDCl_3_ and compared it to the one adopted by the receptor in D_2_O. NOESY analysis revealed strong through-space interactions between H-1 of the pyranose ring and the adjacent ethylene glycol protons of the crown ether fragment (Figure S1). However, no NOEs between the H-4 and H-6 protons of the glucopyranose and the crown ether protons were observed (Figure S1), which were present in the NOESY spectra of **1** in D_2_O. These findings suggest that the tetraethylene glycol fragment in **1** extends below the pyranose ring in CDCl_3_ rather than in a perpendicular fashion, as was the case in D_2_O (Figure 3b). This is further supported by considerable downfield shifts of H-2 and in particular H-3 in the ^1^H NMR spectrum in CDCl_3_ compared to D_2_O (Figure 3a). Such a shift could arise as a result of H-bonding interactions between the 2- and 3-OH groups of the glucopyranose and the oxygen atoms of the crown ether fragment—an interaction that can only be established if the crown ether is located below the pyranose ring.

**Figure 4:**
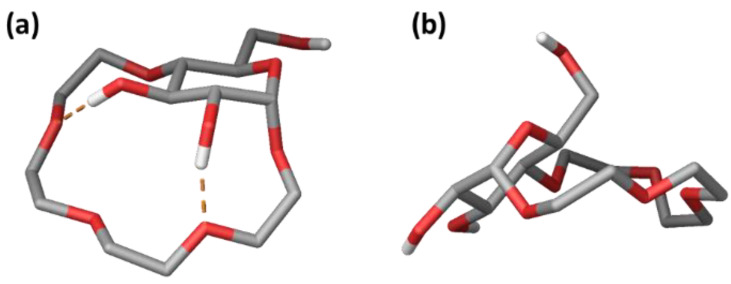
Lowest energy conformation of **1** obtained with MacroModel using: (**a**) CHCl_3_ and (**b**) D_2_O as implicit solvent models.

The results of the NMR experiments were further validated by computational modelling. We performed a conformational search (mixed-torsional/low-mode sampling; 10, 000 steps) using MacroModel, with chloroform as an implicit solvent model. The resulting unique conformers were optimized using the OPLSe3 Force Field, and the structures were sorted by relative potential energy (5 kcal/mol energy window). The calculations revealed an ensemble of similar conformers within an energy window of 2 kcal/mol (Figure S2). The lowest energy conformation of receptor **1** highly resembled the one proposed based on the NOE analysis, with the tetraethylene glycol fragment below the glucopyranose unit and two H-bonding interactions between 2-OH and 3-OH and the oxygen atoms of the tetraethylene glycol (Figure 4a).

#### 2.3.2. Binding Mode Analysis of **1** with H-Val-OMe × HCl

**Figure 5:**
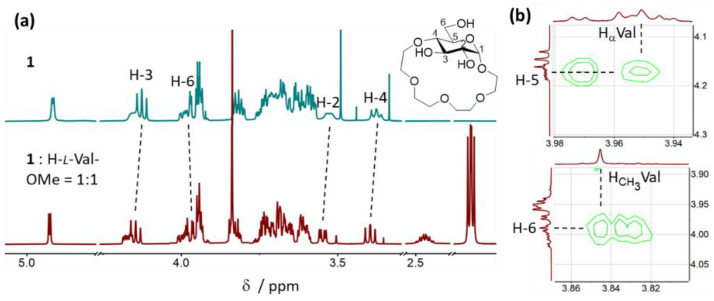
(**a**) ^1^H NMR spectra in CDCl_3_ of **1** (top) and **1** + H-*l*-Val-OMe × HCl (bottom). (**b**) Through-space interactions observed in the NOESY spectra of the 1:1 complex.

Since H-Val-OMe was bound by receptor **1** in CDCl_3_ with the highest affinity and selectivity, we decided to study the interactions between **1** and valine in detail to shed light on the binding mode of amino acid methyl esters to **1**. For this purpose, we investigated the NMR data from the titration experiments and performed NOESY analyses of the 1:1 complexes between **1** and both *l*- and *d*-Val-OMe × HCl. Upon addition of H-*l*-Val-OMe to the CDCl_3_ solution of receptor **1**, a considerable downfield shift of signals of the glucopyranose protons, in particular, H-2, H-3 and H-4, as well as several tetraethylene glycol protons, was observed (Figure 5a). These shifts are consistent with the binding of an ammonium cation inside the crown ether cavity of **1**. Interestingly, an upfield shift of the signal corresponding to H-6 of the pyranose unit was detected (Figure 5a). This suggests that upon formation of the host:guest complex, a possible intramolecular hydrogen bond between the 6-OH and a neighboring oxygen atom in **1** is broken and that the 6-OH could potentially engage in interactions with the guest. NOE analysis of the 1:1 mixture of **1** and H-*l*-Val-OMe revealed through-space interactions between H_α_ of valine and H-5 of the pyranose as well as between the CH_3_ of the methyl ester and H-6 of the pyranose (Figure 5b). No NOEs between H_β_ or H_γ_ of valine and the receptor were observed.

The pattern of changes in the chemical shifts of receptor proton signals in the ^1^H NMR spectrum was similar when H-*d*-Val-OMe was titrated to the CDCl_3_ solution of **1**. However, the amplitudes of changes were in general smaller, indicative that perhaps the ammonium cation is not bound as tightly inside the cavity, and the interactions between 6-OH and the guest are weaker. NOE analysis of the 1:1 mixture of **1** and H-*d*-Val-OMe revealed through-space interactions between the CH_3_ of the methyl ester and the tetraethylene glycol protons in **1** (Figure S3). No NOEs between H_α_ of Val and the receptor were observed, but an interaction between H_β_ and H-5 of glucopyranose was found (Figure S3).

**Figure 6:**
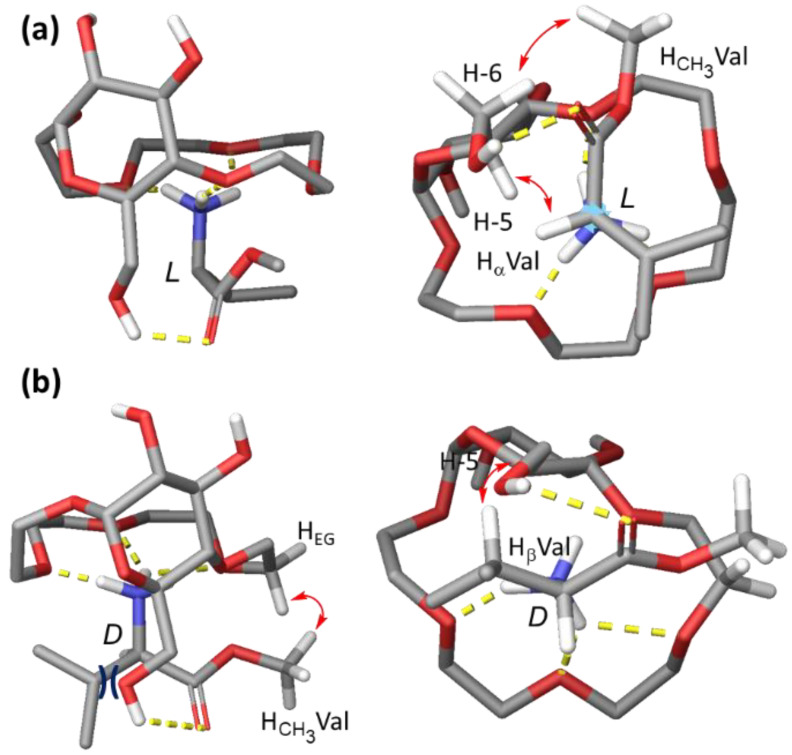
Models of the complexes between **1** and H-*l*-Val-OMe (**a**) H-*d*-Val-OMe (**b**)—side view (left), bottom view (right). Models were obtained by manual adjustment of the calculated lowest energy conformers after sampling of the non-covalent interactions with MacroModel (NOEs observed in the NOESY spectra marked with red arrows, H-bonds marked in yellow, steric repulsion indicated in dark blue).

Based on the NMR analysis, we propose the following binding mode of Val-OMe × HCl by receptor **1** in CDCl_3_. The binding occurs predominantly by a combination of Coulombic and H-bonding interactions between the tetraethylene glycol unit of the host and the ammonium cation of the guest. Additionally, H-bonding interactions between 6-OH of the glucopyranose unit in the host and the carbonyl oxygen of the methyl ester stabilize this complex. In the case of the *l*-enantiomer, establishing these two interactions allows the bulky isopropyl group in valine to be exposed to the solvent, whereas the H_α_ points in the direction of the glucose scaffold (Figure 6a). On the other hand, when the *d*-enantiomer of the guest is bound via the above-described interactions, the isopropyl side chain points towards the glucopyranose ring, leading to steric repulsion and thus considerably decreasing the association constant of the host:guest complex (Figure 6b). The proposed binding mode was also supported by computational modelling (Figure 6a,b).

### 2.4. Discussion of the Proposed Binding Mode

#### 2.4.1. Interpretation of Binding Data Obtained for Ala, Thr, Phe and Pro

The data obtained in the titration experiments with methyl esters of Ala, Thr and Pro in CDCl_3_ are in good agreement with the proposed binding mode of the host-guest complexes. Alanine was bound by receptor **1** with a slightly lower affinity than valine (K_a_*l* = 707 M^−1^ vs. 977 M^−1^) and considerably lower enantioselectivity (2.5:1 vs. 4.7:1). The latter is most likely the result of a smaller steric demand of the methyl group in the side-chain of Ala compared to the isopropyl in Val. The lower association constant of the complex between **1** and H-*l*-Ala-OMe, compared to H-*l*-Val-OMe, indicates that additional interactions (most likely hydrophobic in nature) are involved in the binding of valine by **1**. Threonine was bound with a similar affinity and selectivity as alanine (K_a_*l* = 680 M^−1^, K_a_*l*/K_a_*d* = 2.3:1). The affinity measured for the H-*l*/*d*-Thr-OMe complexes further suggests that a hydrophobic character of the guest is beneficial for the binding in CDCl_3_. The moderate binding selectivity, on the other hand, is most likely due to the fact that the OH group in the side chain of threonine can engage in additional interactions which stabilize the complex with the *d*-enantiomer (e.g., H-bonding to the 6-OH or the pyranose oxygen in the host). The lowest binding affinity and low selectivity of H-Pro-OMe binding by **1** (K_a_*l* = 255 M^−1^, K_a_*l*/K_a_*d* = 1.6:1) are most probably due to the secondary ammonium cation present in proline which interacts more weakly with the crown ether fragment of the receptor than the primary one in other amino acids. Thus, the potential steric clash (or favorable hydrogen bonding interactions) with the glucopyranose unit of one or the other enantiomer of the guest are weakened. In the case of experiments with H-*l*/*d*-Phe-OMe, both the affinity and selectivity measured for the complexes with **1** were lower than expected if we consider the proposed binding mode (K_a_*l* = 315 M^−1^, K_a_*l*/K_a_*d* = 1.5:1). This was likely caused by the lower solubility of H-Phe-OMe × HCl in CDCl_3_, forcing us to use 10 vol% of DMSO-d_6_ in these titration experiments. We showed earlier that the binding affinity of **1** to amino acid methyl esters in DMSO-d_6_ is greatly diminished compared to other solvents, and the enantioselectivity also drops to a significant extent. This detrimental effect probably also manifested itself in the experiments involving H-Phe-OMe × HCl performed in CDCl_3_. Furthermore, the presence of a highly coordinating solvent could also result in an altered binding mode of Phe to **1** since the attractive interactions between the ammonium cation in H-Phe-OMe and the crown ether moiety in **1** would be strongly attenuated.

#### 2.4.2. Additional Titration Experiments

To further validate the proposed binding mode of **1** to amino acids in CDCl_3_, we performed additional titration experiments using leucine, tert-leucine and asparagine methyl esters as guests. The results of these experiments are summarized in Table 2.

Leucine methyl ester was bound with a slightly lower affinity and lower enantioselectivity than valine methyl ester. On the other hand, tert-leucine ester was bound with a notably lower affinity but higher enantioselectivity than valine. Asparagine was bound with the lowest affinity and virtually no enantioselectivity.

The obtained results provide further support to our proposed binding mode of amino acid methyl ester salts by receptor **1** in CDCl_3_. The lower enantioselectivity of leucine binding, in comparison to valine, most likely arises from the fact that the bulky part of the side chain in leucine is located one CH_2_ group further away from the chiral center, and thus the steric clash between this group in the *d*-enantiomer of the guest and the pyranose ring is smaller than in the case of valine. For the same reason, the enantioselectivity of t-Leu binding is higher than that of Val. The tert-butyl side-chain in H-*d*-t-Leu-OMe causes a larger steric hindrance when binding to the receptor than the isopropyl group in H-*d*-Val-OMe. The increased steric bulk of t-Leu also leads to an overall diminished binding affinity or **1** towards this amino acid. Asparagine contains a primary amide in the side-chain, which is capable of engaging in H-bonding interactions. Thus, both the methyl ester and the amide in H-Asn-OMe can serve as H-bond acceptors for the 6-OH of the glucose crown ether **1** when either the *l*- or the *d*-enantiomer of the guest is bound, resulting in very similar association constants for both complexes.

#### 2.4.3. Comparison of Binding Modes in CDCl_3_ and D_2_O

**Figure 7:**
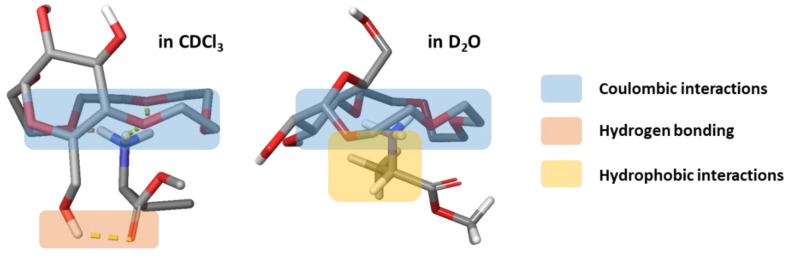
Proposed binding modes of *l*-amino-acid methyl esters by **1** in CDCl_3_ (**left**) and D_2_O (**right**). The main interactions contributing to the binding are marked with colored rectangles.

The proposed binding modes of amino acid methyl esters to receptor **1** in CDCl_3_ and D_2_O (discussed in detail in ref. [23]) bear a certain similarity. The main factor responsible for the complex formation in both solvents are interactions between the ammonium group of the guest and the crown-ether moiety of the host. Otherwise, we hypothesize that in D_2_O, hydrophobic interactions between the host and the side chain of the guest play an important role, whereas in CDCl_3_, H-bonding between the 6-OH of the glucopyranose in **1** and the carbonyl oxygen of the methyl ester in the guest is the secondary contributor to binding (Figure 7).

We presume that hydrophobic interactions between the guests and **1**, pronounced in D_2_O, cannot also be entirely neglected in CDCl_3_ and increase the binding affinity of **1** towards amino acids with hydrophobic side-chains such as Val and Leu.

## 3. Conclusions and Outlook

In conclusion, we have demonstrated that the glucose-based crown ether can be used for the chiral recognition of amino acid methyl ester salts in organic solvents. We have shown that the binding affinities and selectivities displayed by receptor **1** in organic media are strongly dependent on the coordinating ability of the solvent, with the poorest results obtained in DMSO-d_6_ and the best in CDCl_3_. By conducting an in-depth study using NMR spectroscopy and computational modelling, we were able to propose a plausible binding mode of receptor **1** towards amino acid guests in CDCl_3_. The proposed binding mode facilitated the interpretation of obtained binding data and was further supported by experiments with additional amino acid guests. We also performed a comparative analysis of the binding properties of receptor **1** in CDCl_3_ and D_2_O, discussing the main types of interactions responsible for the affinity of the receptor towards amino acids displayed in both solvents. This detailed analysis should facilitate the development of further monosaccharide-based receptors for the binding of amino acids and related guests with high affinities and stereo- or chemoselectivities both in aqueous and organic media.

## 4. Materials and Methods

**Materials**

Solvents and reagents were of the highest commercially available grade and were used without further purification. They were purchased from Sigma Aldrich (Buchs, Switzerland), Fischer Scientific (Wohlen, Switzerland), Bachem (Bubendorf, Switzerland), Biotage (Uppsala, Sweden), IRIS Biotech (Marktredwitz, Germany), Gyros Protein Technologies AB (Uppsala, Sweden). Solvents used for MPLC were HPLC-grade quality.

**Preparative medium pressure liquid chromatography (MPLC)**

Purifications of the building blocks were carried out on a CombiFlash EZ Prep flash chromatography system (Teledyne ISCO, Lincoln, NE, USA). Two different solvent sets were used: 1. Solvent A was HPLC-grade DCM without stabilizer, and solvent B was HPLC-grade methanol for the building blocks. 2. Solvent A was HPLC-grade hexane, and solvent B was HPLC-grade ethyl acetate.

**Thin-layer chromatography (TLC)**

TLC was conducted on aluminium sheets coated with silica gel 60 F254 (Merck, Zug, Switzerland) using UV fluorescence (254 and 366 nm). Analytical grade solvents were used.

**Liquid chromatography-mass spectrometry (LC-MS)**

Analytical reverse-phase HPLC (RP-HPLC) was performed on a Dionex UHPLC, Ultimate 3000. Reprosil gold 120 C18 (150 × 4 mm, 5 µm) with a flow of 0.5 mL/min was used as the analytical column. Two different solvents were used. Solvent A was assigned to be pure acetonitrile, and solvent B was a mixture of 1 % acetonitrile and 0.1 % TFA in Milli-Q pure water. The mass analysis was performed on an amaZone speed ion trap mass analyzer (Bruker, Coventry, UK).

**Nuclear magnetic resonance (NMR) spectroscopy**

Next, 1D and 2D NMR spectra were recorded on 400, 500 and 600 MHz Ultrashield spectrometers (Bruker, Coventry, UK). NMR chemical shifts (δH) are quoted in parts per million (ppm), and coupling constants (J) are quoted in Hertz (Hz). Abbreviations for NMR data are s (singlet), d (doublet), t (triplet), q (quartet), and m (multiplet).

**High-resolution mass spectrometry (HR-MS)**

High-resolution electrospray ionization (HR-ESI) spectra were measured on a Bruker maXis spectrometer.

**Computational modelling**

Computational simulations were carried out with the Schrodinger Maestro Suite (Program version 2021-2 for MacOS, Portland, OR, USA). Conformational searches were performed in MacroModel using the Mixed-torsional/Low mode conformational sampling starting from a non-optimized input geometry. Unique conformer geometries were optimized with the OPLS3e force field (GB/SA chloroform implicit solvation model) and sorted by relative potential energy. The conformational search was performed with 10,000 steps, and a 21 kJ/mol (5 kcal/mol) relative potential energy cut-off was applied (0.5 Å maximum atom deviation).

Non-covalent interactions were modeled by merging the host and the guest structure, followed by a conformational search in MacroModel. The same parameters were applied as stated above, but 25,000 steps were used per search.

## Figures and Tables

**Table 1:** Association constants of complexes between receptor **1** and amino acid methyl ester HCl salts ^1^.

Amino Acid	DMSO-d_6_			CD_3_CN			CDCl_3_			D_2_O		
	K_a_*l *^2^	K_a_*d*	Sel.	K_a_*l*	K_a_*d*	Sel.	K_a_*l*	K_a_*d*	Sel.	K_a_*l*	K_a_*d*	Sel.
Ala	98 ± 26	51 ± 14	1.8:1	299 ± 94	139 ± 55	2.2:1	707 ± 308	279 ± 103	2.5:1	133 ± 14	69 ± 9	1.9:1
Thr	104 ± 10	61 ± 11	1.4:1	306 ± 81	201 ± 44	1.5:1	680 ± 213	295 ± 80	2.2:1	166 ± 22	61 ± 9	2.7:1
Val	189 ± 72	52 ± 12	3.6:1	405 ± 98	117 ± 26	3.5:1	977 ± 266	201 ± 34	4.7:1	232 ± 43	46 ± 5	5.1:1
Phe	167 ± 43	54 ± 12	3.1:1	420 ± 161 ^3^	141 ± 22 ^3^	3.0:1	315 ± 59 ^3^	212 ± 37 ^3^	1.5:1	275 ± 30	45 ± 5	6.2:1
Pro	72 ± 15	32 ± 2	2.2:1	132 ± 32	72 ± 26	1.9:1	255 ± 74	162 ± 50	1.6:1	70 ± 5	44 ± 5	1.6:1

^1^ The stoichiometry of complexes was determined to be 1:1 based on the fitting of data to different binding models. ^2^ K_a_s are reported in M^−1^. The values are given with standard deviations. ^3^ 10 vol% DMSO-d6 was added for solubility reasons.

**Table 2:** Binding affinities of complexes between **1** and methyl ester HCl salts of Leu, t-Leu and Asn ^1^.

Amino-Acid	K_a_*l *^2^	K_a_*d*	Sel.
Leu	833 ± 354	258 ± 91	3.2:1
tLeu	699 ± 249	123 ± 29	5.6:1
Asn	322 ± 108	293 ± 64	1.1:1

^1^ The stoichiometry of complexes was determined to be 1:1 based on data fitting to binding models. ^2^ K_a_s are reported in M^−1^. The values are given with standard deviations.

## Data Availability

The data presented in this study are available in the article and the Supplementary Materials.

## Supplementary Materials

The following supporting information can be downloaded at: https://www.mdpi.com/article/10.3390/molecules27072177/s1: Figure S1: Fragments of the NOESY spectrum of **1** in CDCl_3_; Figure S2: Overlay of lowest energy conformers (within 1 kcal/mol window, left; within 2 kcal/mol window, right) of receptor **1** obtained using the OPLSe3 Force Field calculations using CHCl_3_ as solvent model; Figure S3: Fragments of the NOE spectrum of the 1:1 complex between **1** and H-*d*-Val-OMe in CDCl_3_; Characterization of compound **1**; Titration tables; NMR spectra and titration curves; NMR spectra of the complexes.
