# Supplementary material for: Chiral Recognition of Amino Acid Esters in Organic Solvents Using a Glucose-Based Receptor
Source: Molecules. 2022 Mar 28;27(7):2177. doi: 10.3390/molecules27072177 (PMC9000842; doi:10.3390/molecules27072177)
Supplement: Supplementary file 1 [file molecules-27-02177-s001.zip › molecules-1652425-supplementary.pdf]

Supporting Information

Index

1. **Supplementary figures for the manuscript.** 3
2. **Materials, methods and instruments.** 5
3. **Characterisation of receptor 1 in $CDCl_3$, CD_3CN and DMSO-d_6.** 6
4. **Titration experiments monitored by ^1H NMR.** 7
   - **4.1 Titrations in $CDCl_3$.** 8
   - **4.2 Titrations in CD_3CN.** 40
   - **4.3 Titrations in DMSO-d_6.** 60
5. **NMR spectra of receptor 1.** 80
6. **NMR spectra of 1 + H-Val-OMe complexes in $CDCl_3$.** 89
7. **HR MS spectrum of receptor 1.** 92

1. Supplementary figures for the manuscript.

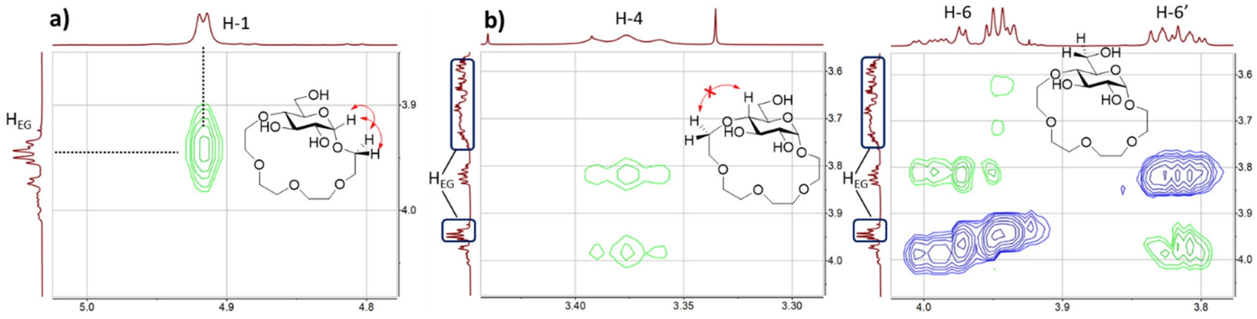

Figure S1 Fragments of the NOESY spectrum of **1** in $CDCl_3$: a) NOE between H-1 and the adjacent ethylene glycol protons; b) lack of NOEs between H-4 or H-6 and the ethylene glycol protons.

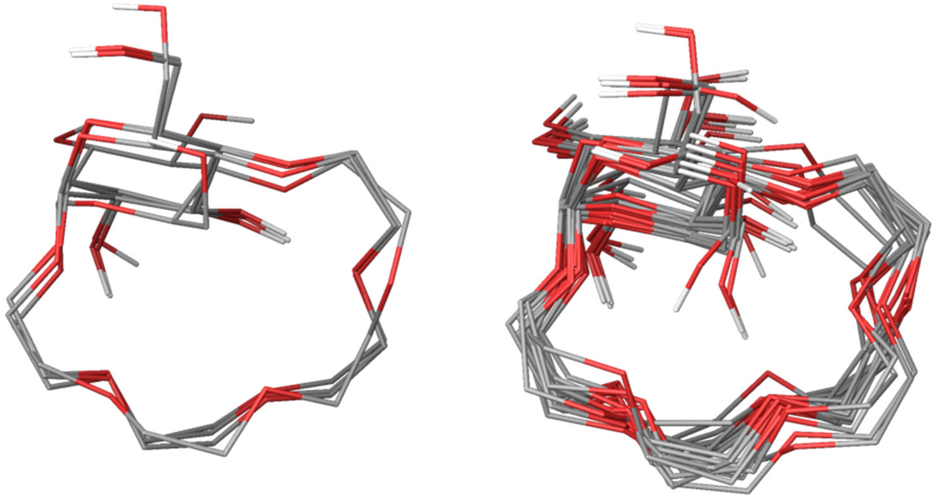

Figure S2 Overlay of lowest energy conformers (within 1 kcal/mol window, left; within 2 kcal/mol window, right) of receptor **1** obtained using the OPLSe3 Force Field calculations using $CHCl_3$ as solvent model.

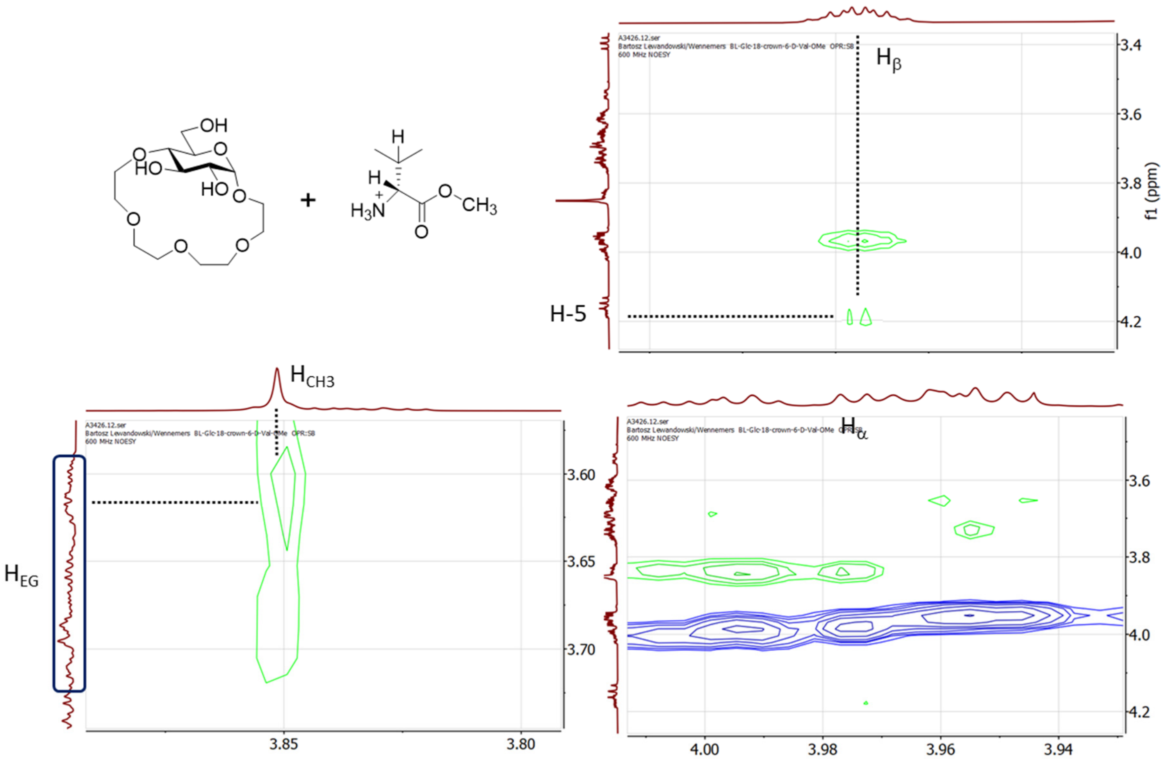

Figure S3 Fragments of the NOE spectrum of the 1:1 complex between **1** and H-D-Val-OMe in $CDCl_3$ depicting: NOEs between H_{CH3} of the guest and ethylene glycol protons of the host, bottom left; NOEs between H_β of the guest and H-5 of the host, top right; lack of NOEs between H_α of the guest and the host, bottom right.

2. Materials, methods and instruments

Materials

Solvents and reagents were of the highest commercially available grade and used without further purification. They were purchased from Sigma Aldrich, Fischer Scientific, Fluka, Bachem, BioMatrix, Biotage, IRIS Biotech, Protein Technologies and Acros Organics. Solvents used for MPLC were HPLC-grade quality.

Preparative medium pressure liquid chromatography (MPLC)

Purifications of the building blocks were carried out on a CombiFlash EZ Prep flash chromatography system (Teledyne ISCO). Two different solvent sets were used: 1. Solvent A was HPLC-grade DCM without stabilizer and solvent B was HPLC-grade methanol; 2. Solvent A was HPLC-grade hexane and solvent B HPLC-grade EtOAc.

Thin-layer chromatography (TLC)

TLC was conducted on aluminium sheets coated with silica gel 60 F_{254} (Merck) using UV fluorescence (254 and 366 nm). Analytical grade solvents were used.

Liquid chromatography - mass spectrometry (LC - MS)

Analytical reverse phase HPLC (RP-HPLC) was performed on a Dionex UHPLC, Ultimate 3000. Reprosil gold 120 C_{18} (150 x 4 mm, 5 µm) with a flow of 0.5 mL/min was used as the analytical column. Two different solvents were used. Solvent A was assigned to be pure acetonitrile and solvent B was a mixture of 1 % acetonitrile and 0.1 % TFA in Milli-Q pure water. The mass analysis was performed on an amaZone speed ion trap mass analyzer (Bruker, USA).

Nuclear magnetic resonance (NMR) spectroscopy

1D and 2D NMR spectra were recorded on 400 and 500 MHz Ultrashield spectrometers (Bruker, USA). NMR chemical shifts (δ_H and δ_C) are quoted in parts per million (ppm) and coupling constants (J) are quoted in Hertz (Hz). Abbreviations for NMR data are s (singlet), d (doublet), t (triplet), q (quartet), m (multiplet).

High-resolution mass spectrometry (HR-MS)

High-resolution electrospray ionization (HR-ESI) spectra were measured on a Bruker maXis spectrometer.

Computational modelling

Computational simulations were performed with the Schrodinger Maestro Suite (Program version 2020-2 for MacOS). A conformational search was carried out starting from a random input geometry of receptor **1**, using the Mixed-torsional/Low mode conformational sampling of MacroModel combined with OPLS3e force field minimization (GB/SA water solvation model). The conformational search was performed with 10000 steps and a 21kJ/mol (~ 5 kcal/mol) relative potential energy cut-off was applied (0.5 Å maximum atom deviation).

All resulting conformers within 21 kJ/mol (~ 5 kcal/mol) of the global minimum were clustered according to the RMSD of the heavy atoms. The structure closest to the centroid in each cluster was chosen as a representative conformation. Representative conformers in between ~ 2 kcal/mol were superimposed for structural comparison.

3. Characterisation of receptor 1 in $CDCl_3$, CD_3CN and DMSO-d_6.

The receptor was synthesised according to a procedure described in ref.23.

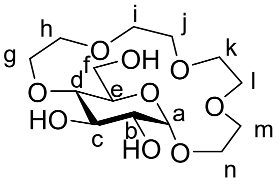

HR-ESI-MS: m/z: 361.1484 $[M + Na]^+$, calculated for $C_{14}H_{26}NaO_9^+$: 361.1485

$CDCl_3$:

1H NMR (600 MHz, $CDCl_3$) δ 4.92 (d, *J* = 3.5 Hz, 1H, H_a), 4.16 (m, 1H, H_e), 4.13 (m, 1H, H_c), 4.01 - 3.93 (m, 4H, $H_f + H_g + H_n$), 3.84 - 3.79 (m, 2H, $H_{f'} + H_{g'}$), 3.76 - 3.56 (m, 13H, $H_{h,i,j,k,l,m}$), 3.52 (d, *J* = 9.2 Hz, 1H, H_b), 3.38 (m, 1H, H_d).

^{13}C NMR (150 MHz, $CDCl_3$) δ 98.4 (C_a), 78.5 (C_d), 73.0 (C_b), 72.9 (C_c), 71.37 + 71.18 (2 ×C, $C_{h/i/j/k/l/m}$), 71.11 (C_e), 70.54 + 70.35 (double intensity, 4 × C, $C_{h/i/j/k/l/m}$), 68.61 (C_g), 67.47 (C_n), 62.84 (C_f).

DMSO-d_6:

^1H NMR (600 MHz, DMSO-d_6) δ 4.77 (d, *J* = 5.4 Hz, 1H, 3-OH), 4.76 (d, *J* = 5.4 Hz, 1H, 2-OH), 4.61 (d, *J* = 3.4 Hz, 1H, H_a), 4.34 (dd, *J* = 6.6, 4.8 Hz, 1H, 6-OH), 3.94 (ddd, *J* = 10.0, 4.0, 2.0 Hz, 1H, H_e), 3.76 - 3.67 (m, 4H, $H_c + H_f + H_g + H_n$), 3.63 - 3.40 (m, 15H, $H_{f'} + H_{g,h,i,j,k,l,m}$), 3.22 (m, 1H, H_d), 3.16 (m, 1H, H_b).

^{13}C NMR (150 MHz, DMSO-d6) δ 99.1 (C_a), 76.7 (C_d), 72.9 (C_b), 71.6 ($C_{h/i/j/k/l/m}$), 70.8 (2×C, $C_c + C_{h/i/j/k/l/m}$), 70.6, 70.4, 70.3, 70.2 (4×C, $C_{h/i/j/k/l/m}$), 70.1 (C_e), 67.1 (C_g), 67.1 (C_n), 61.0 (C_f).

CD_3CN:

^1H NMR (600 MHz, MeCN-d_3) δ 4.73 (d, *J* = 3.5 Hz, 1H, H_a), 4.11 - 4.07 (ddd, J = 10.0, 4.4, 2.7 Hz, 1H, H_e), 3.82 - 3.73 (M, 5H, $H_c + H_f + H_g + H_n$), 3.72 - 3.53 (m, 12H, $H_{f'} + H_{g'} + H_{h,i,j,k,l,m}$), 3.50 - 3.44 (m, 2H, $H_{h,i,j,k,l,m}$), 3.28 (d, *J* = 6.0 Hz, 1H, H_b), 3.21 (dd, *J* = 10.0, 8.8 Hz, 1H, H_d), 3.02 (bs, 1H, 2-OH), (m, 1H, 6-OH).

^{13}C NMR (150 MHz, MeCN-d_3) δ 98.6 (C_a), 77.3 (C_d), 72.8 (C_b), 71.7 (C_c), 71.2, 70.6, 70.4 (3×C, $C_{h/i/j/k/l/m}$), 70.4 (C_e), 70.5, 70.3, 70.2 (3×C, $C_{h/i/j/k/l/m}$), 67.5 (C_f), 67.4 (C_g), 61.9 (C_n).

4. Titration experiments monitored by ^{1}H NMR.

The titration experiments were carried out at room temperature. The concentrations of the host and the guest solutions were 6 mM and 300 mM respectively. The data was fitted to a single-site non-competitive binding equation using Prism ver.9.0.0 (GraphPad).

Injection table (valid for all experiments in $CDCl_3$):

Titration	Portion of guest solution added (µL)	Total volume of guest solution added (µL)	Equivalents of guest added	Concentration of guest (mM)
1	0	0	0	0
2	1	1	0.1	0.6
3	2	3	0.3	1.8
4	3	6	0.6	3.6
5	4	10	1.0	6.0
6	8	18	1.8	10.8
7	10	28	2.8	16.8
8	12	40	4.0	24

Injection table (valid for all experiments in CD_3CN and DMSO-d_6):

Titration	Portion of guest solution added (µL)	Total volume of guest solution added (µL)	Equivalents of guest added	Concentration of guest (mM)
1	0	0	0	0
2	1	1	0.1	0.6
3	3	4	0.4	2.4
4	4	8	0.8	4.8
5	4	12	1.2	7.2
6	12	24	2.4	14.4
7	12	36	3.6	21.6
8	16	52	5.2	31.2

4.1 Titrations in $CDCl_3$.

H-L-Ala-OMe x HCl to receptor 1:

^{1}H NMR spectra (top – full, bottom – enhanced view of the receptor signals in the spectra):

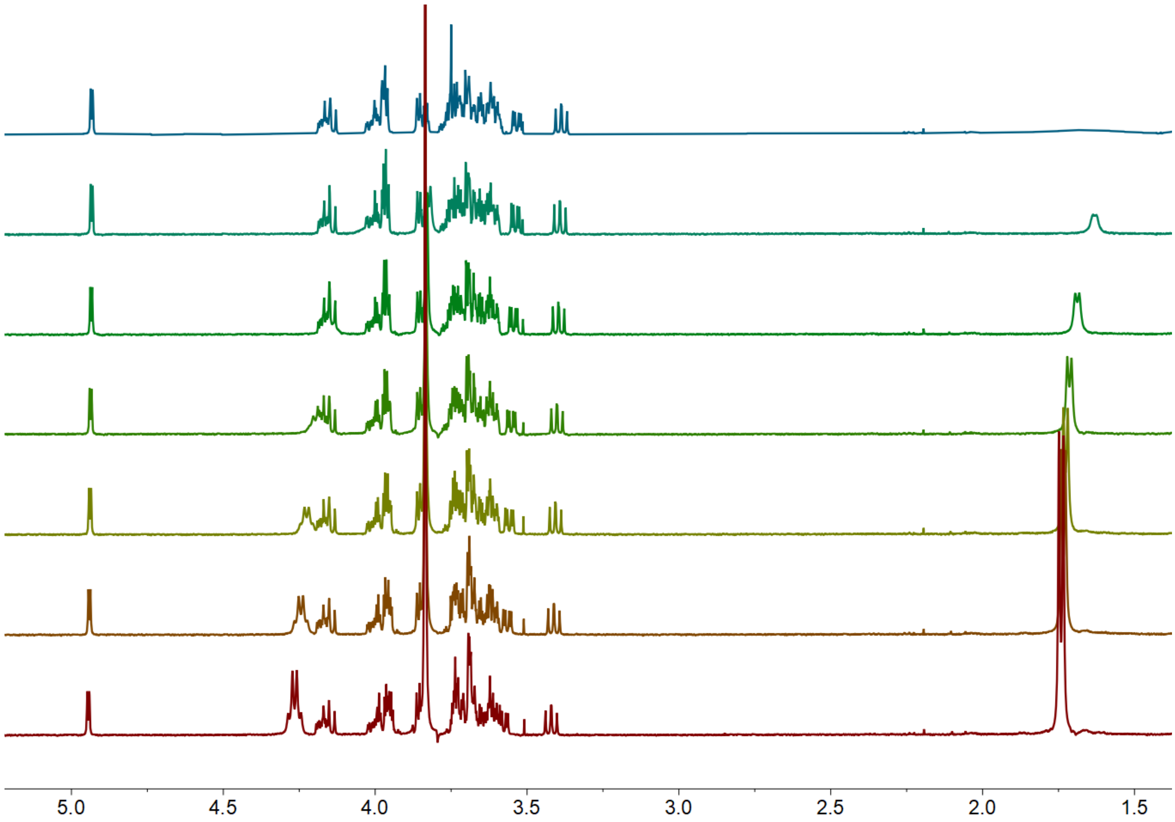

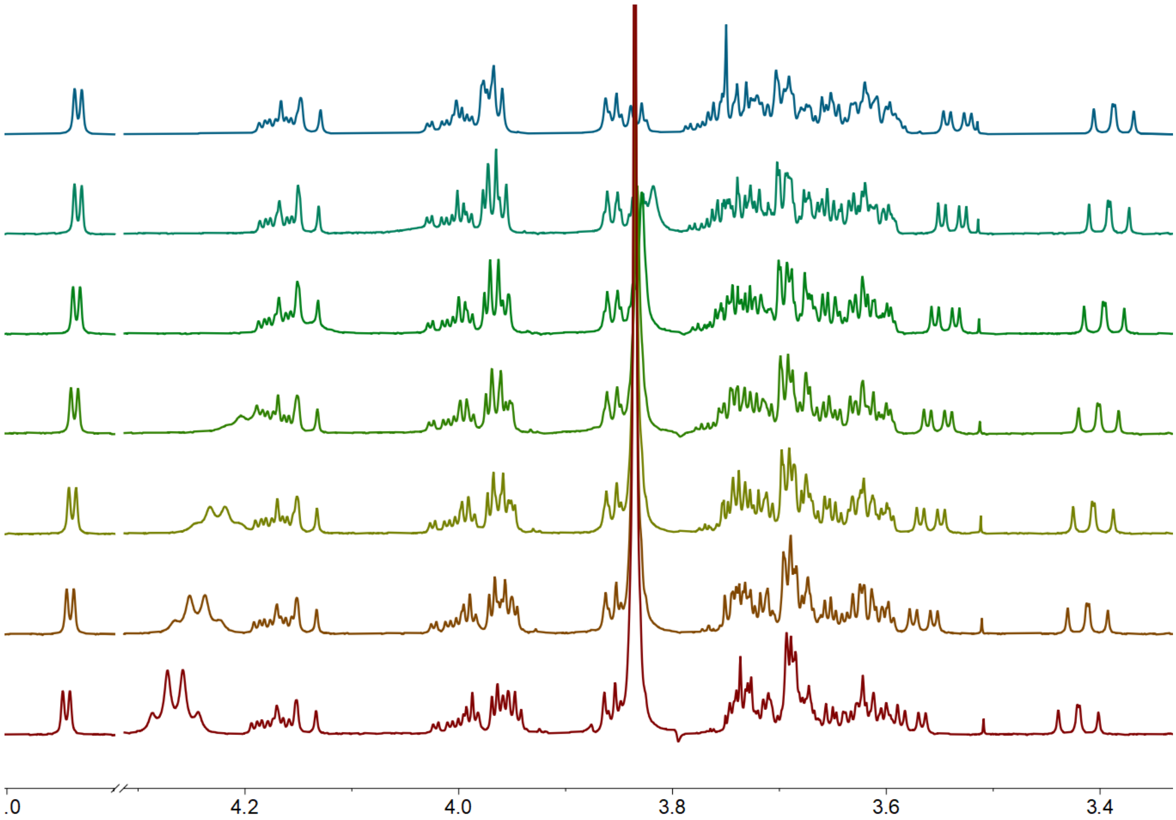

Summary of chemical shift changes:

Titration	4.14	Δppm	4	Δppm	3.87	Δppm	3.58	Δppm
0	4.1456	0.0000	4.0067	0.0000	3.871	0.0000	3.5865	0.0000
1	4.1479	0.0023	4.0023	0.0044	3.8629	0.0081	3.5898	0.0033
2	4.1495	0.0039	4.0012	0.0055	3.8612	0.0098	3.592	0.0055
3	4.1502	0.0046	3.9999	0.0068	3.861	0.0100	3.5933	0.0068
4	4.1509	0.0053	3.9984	0.0083	3.8605	0.0105	3.5941	0.0076
5	4.1512	0.0056	3.9977	0.0090	3.8602	0.0108	3.595	0.0085
6	4.1515	0.0059	3.9966	0.0101	3.8598	0.0112	3.5952	0.0087
7	4.1519	0.0063	3.9947	0.0120	3.8596	0.0114	3.5955	0.0090

Plot of chemical shift change vs guest concentration:

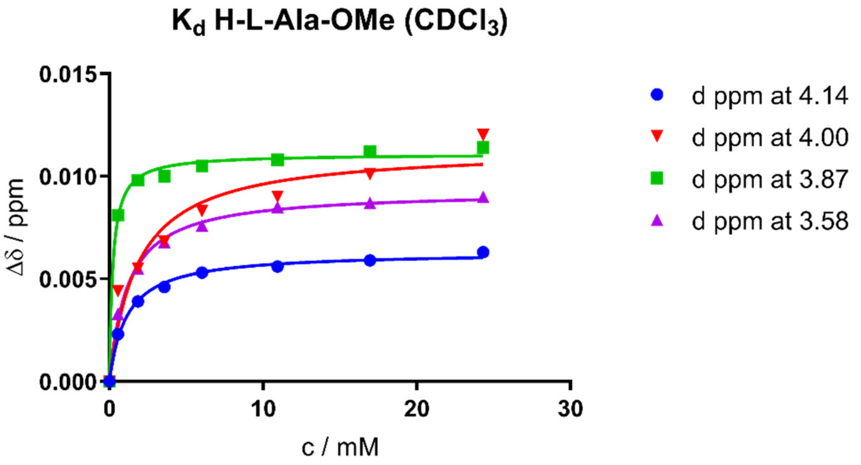

K_d values obtained by fitting the data for selected protons into the single-site specific binding model:

Kd	1.44	2.43	0.56	1.23
**R squared**	0.9917	0.9257	0.9938	0.9933

K_d = 1.42 ± 0.67 mM → K_a = 707 ± 308 M^{-1}

H-D-Ala-OMe x HCl to receptor 1:

^{1}H NMR spectra (top – full, bottom – enhanced view of the receptor signals in the spectra):

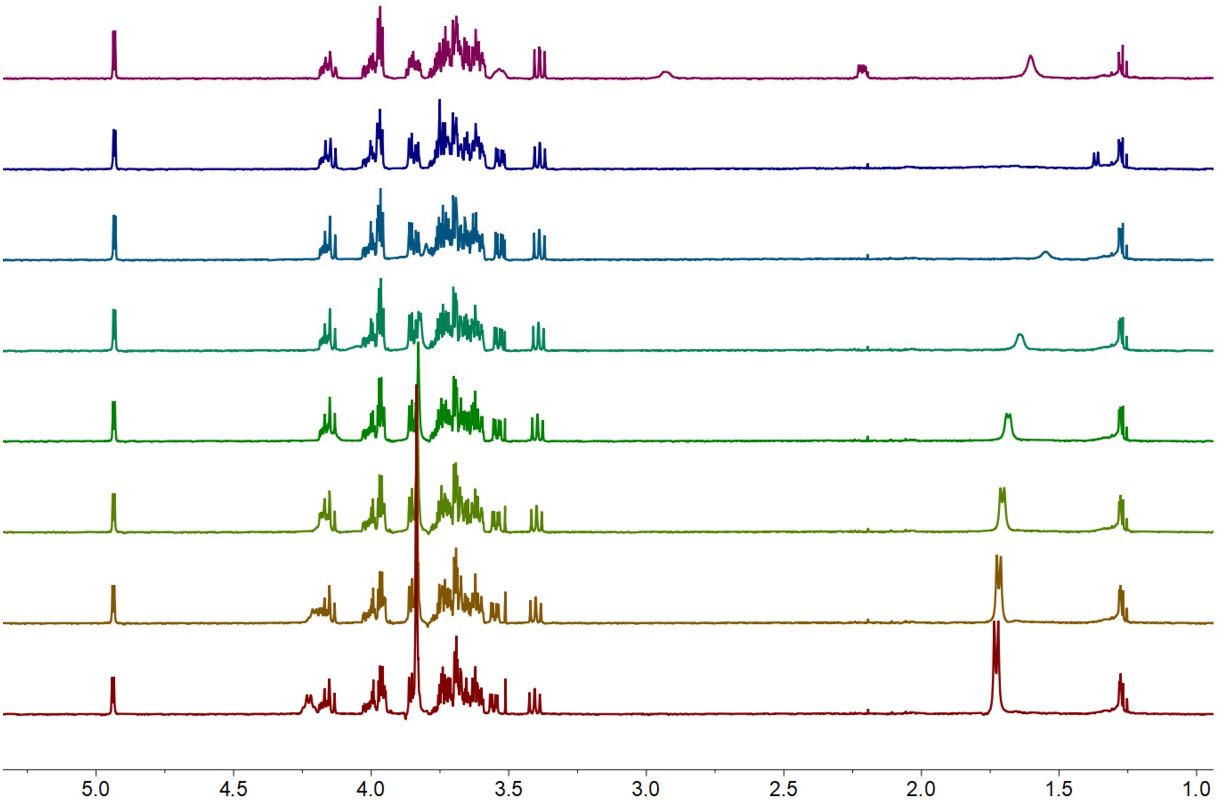

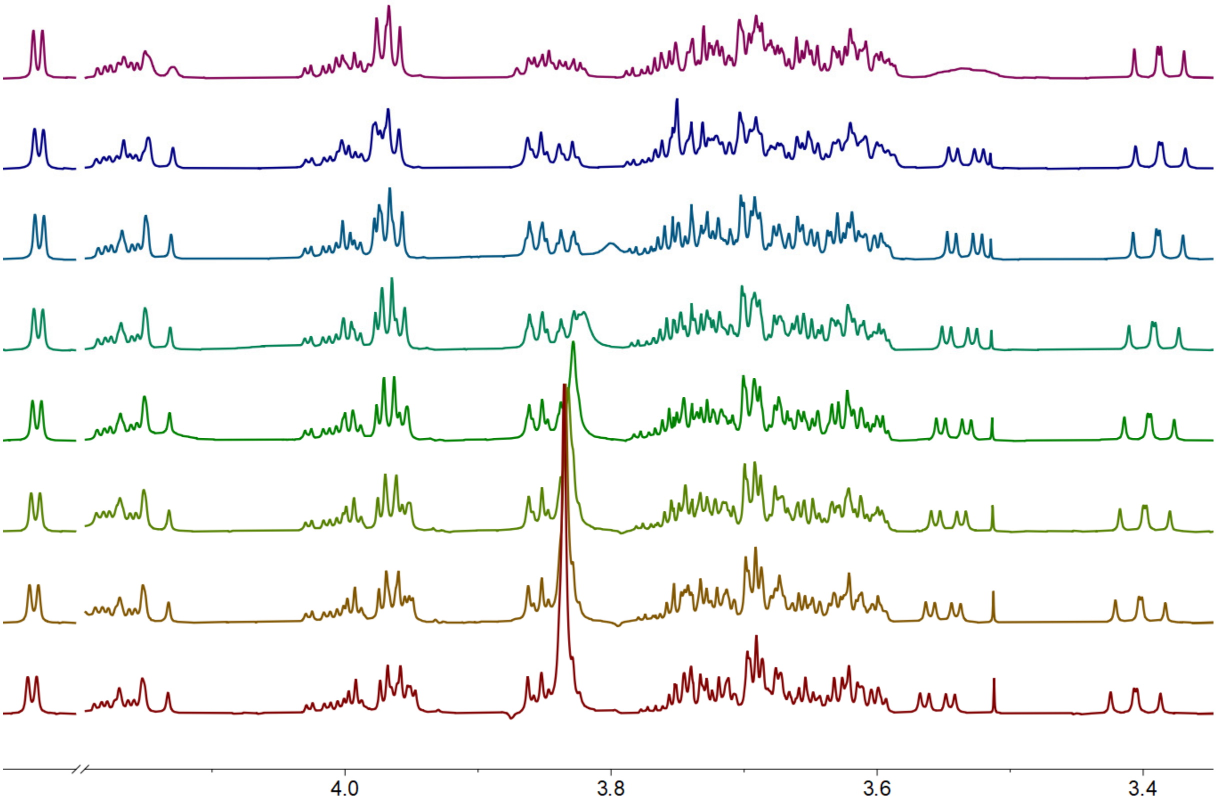

Summary of chemical shift changes:

Titration	4.14	Δppm	3.99	Δppm	3.98	Δppm	3.7	Δppm
0	4.1468	0.0000	3.9991	0.0000	3.9822	0.0000	3.7008	0.0000
1	4.148	0.0012	3.9975	0.0016	3.9789	0.0033	3.6963	0.0045
2	4.1485	0.0017	3.9963	0.0028	3.9779	0.0043	3.6945	0.0063
3	4.1495	0.0027	3.9952	0.0039	3.977	0.0052	3.6936	0.0072
4	4.1503	0.0035	3.9942	0.0049	3.9763	0.0059	3.6925	0.0083
5	4.1509	0.0041	3.9932	0.0059	3.9754	0.0068	3.6918	0.0090
6	4.1516	0.0048	3.9925	0.0066	3.9744	0.0078	3.6913	0.0095
7	4.1522	0.0054	3.9917	0.0074	3.9733	0.0089	3.6906	0.0102

Plot of chemical shift change vs guest concentration:

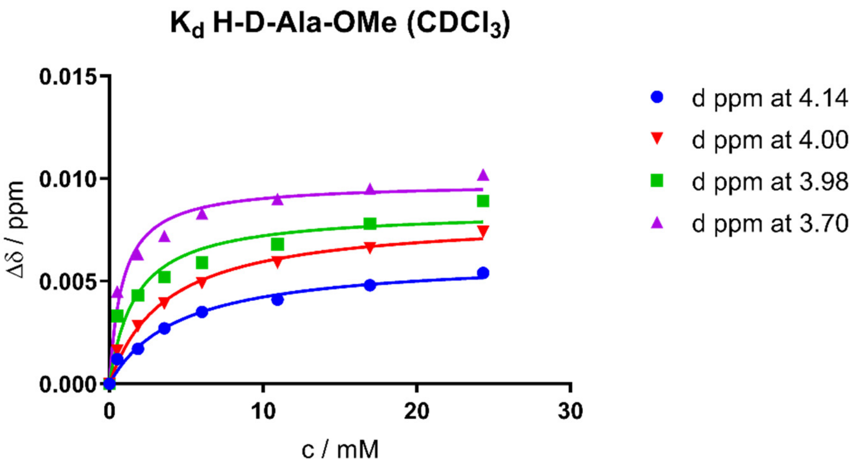

K_d values obtained by fitting the data for selected protons into the single-site specific binding model:

Kd	5.23	4.43	2.84	1.86
R squared	0.9788	0.9869	0.9276	0.9719

K_d = 3.59 ± 1.32 mM → K_a = 279 ± 103 M^{-1}

H-L-Phe-OMe x HCl to receptor 1:

^{1}H NMR spectra (top – full, bottom – enhanced view of the receptor signals in the spectra):

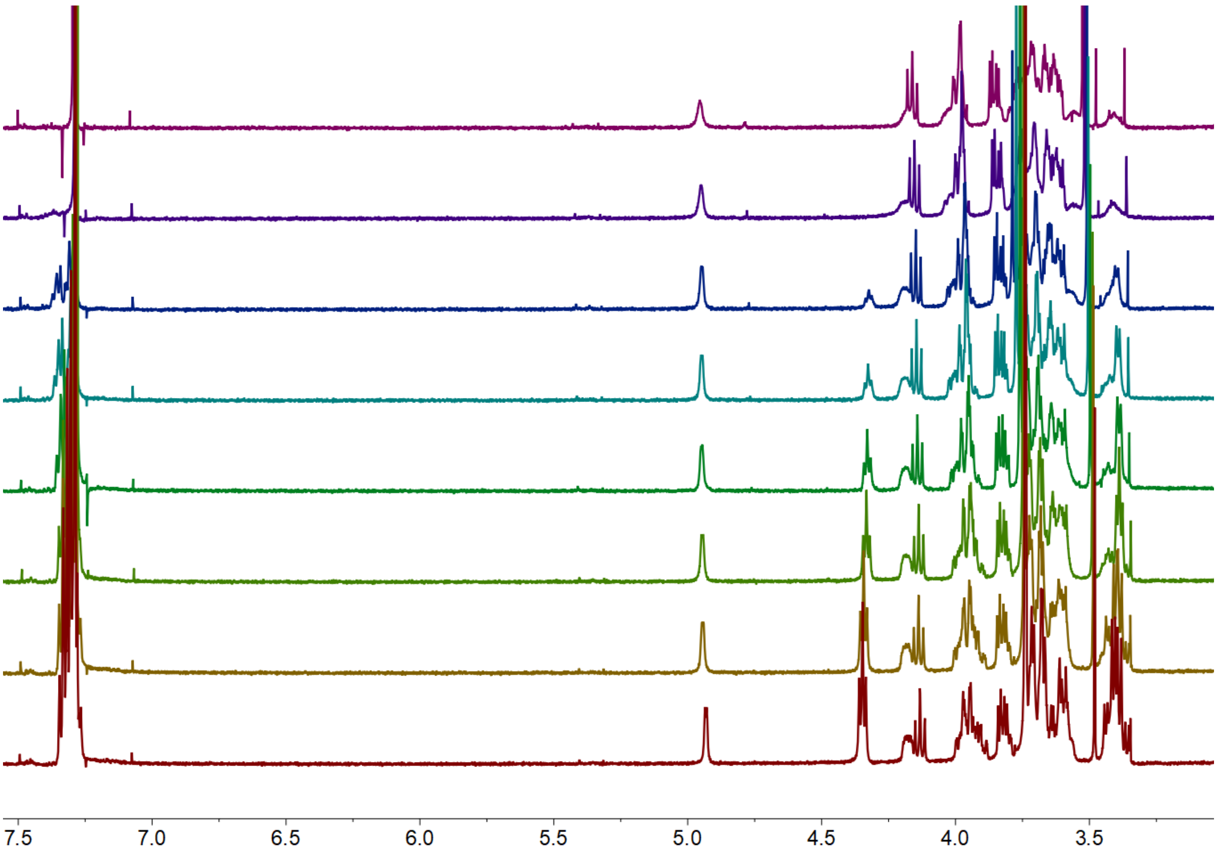

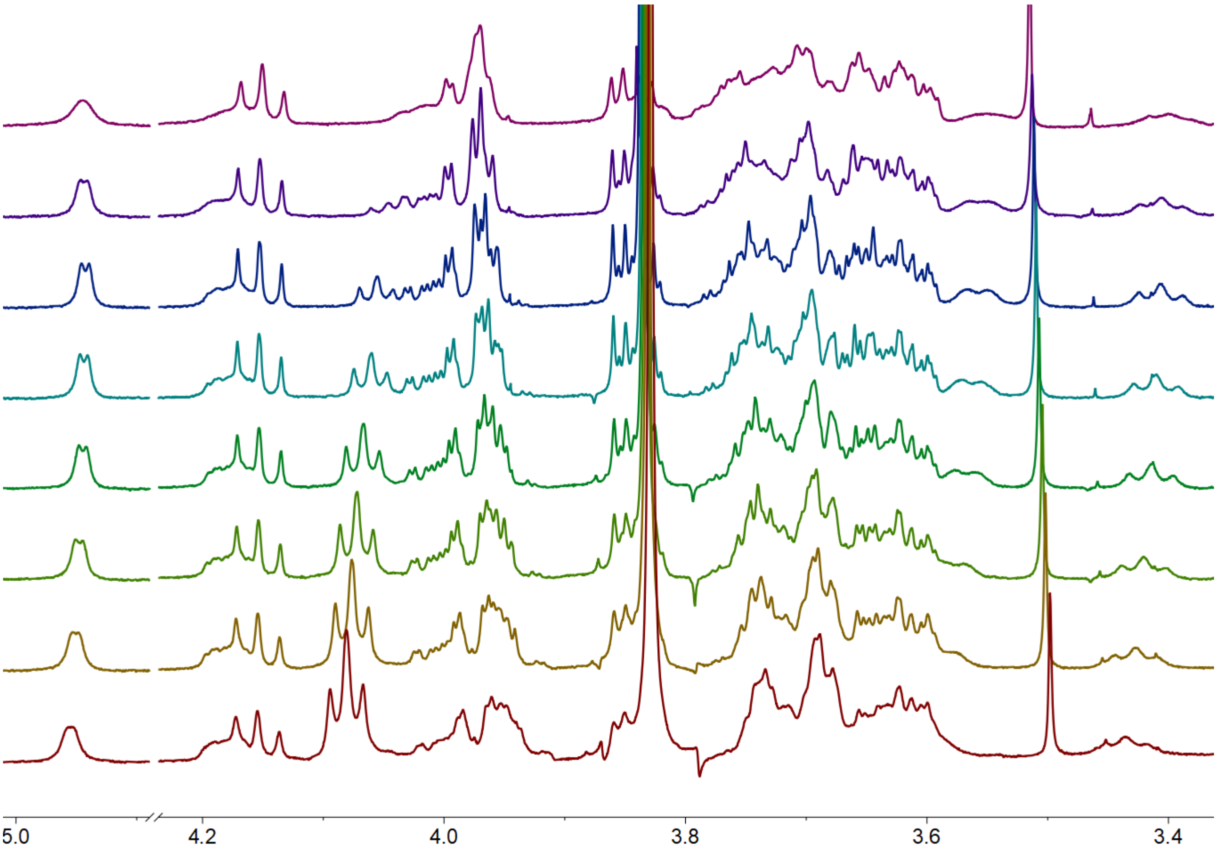

Summary of chemical shift changes:

Titration	4.16	Δppm	4	Δppm	3.96	Δppm	3.6	Δppm
0	4.1614	0.0000	4.0037	0.0000	3.9655	0.0000	3.6079	0.0000
1	4.1535	0.0079	3.9955	0.0082	3.958	0.0075	3.6022	0.0057
2	4.1481	0.0133	3.9895	0.0142	3.9535	0.0120	3.5992	0.0087
3	4.145	0.0164	3.9825	0.0212	3.9493	0.0162	3.5976	0.0103
4	4.1425	0.0189	3.977	0.0267	3.9451	0.0204	3.5956	0.0123
5	4.1398	0.0216	3.9719	0.0318	3.9417	0.0238	3.5912	0.0167
6	4.1372	0.0242	3.9672	0.0365	3.9385	0.0270	3.589	0.0189
7	4.133	0.0284	3.9653	0.0384	3.9371	0.0284	3.5876	0.0203

Plot of chemical shift change vs guest concentration:

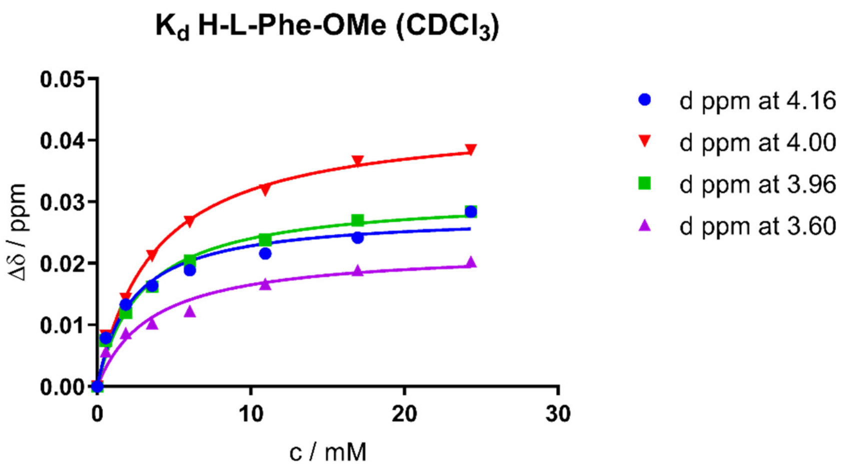

K_d values obtained by fitting the data for selected protons into the single-site specific binding model:

Kd	2.28	3.77	2.98	3.63
**R squared**	0.9632	0.9906	0.9833	0.9592

K_d = 3.17 ± 0.59 mM → K_a = 315 ± 59 M^{-1}

H-D-Phe-OMe x HCl to receptor 1:

^{1}H NMR spectra (top – full, bottom – enhanced view of the receptor signals in the spectra):

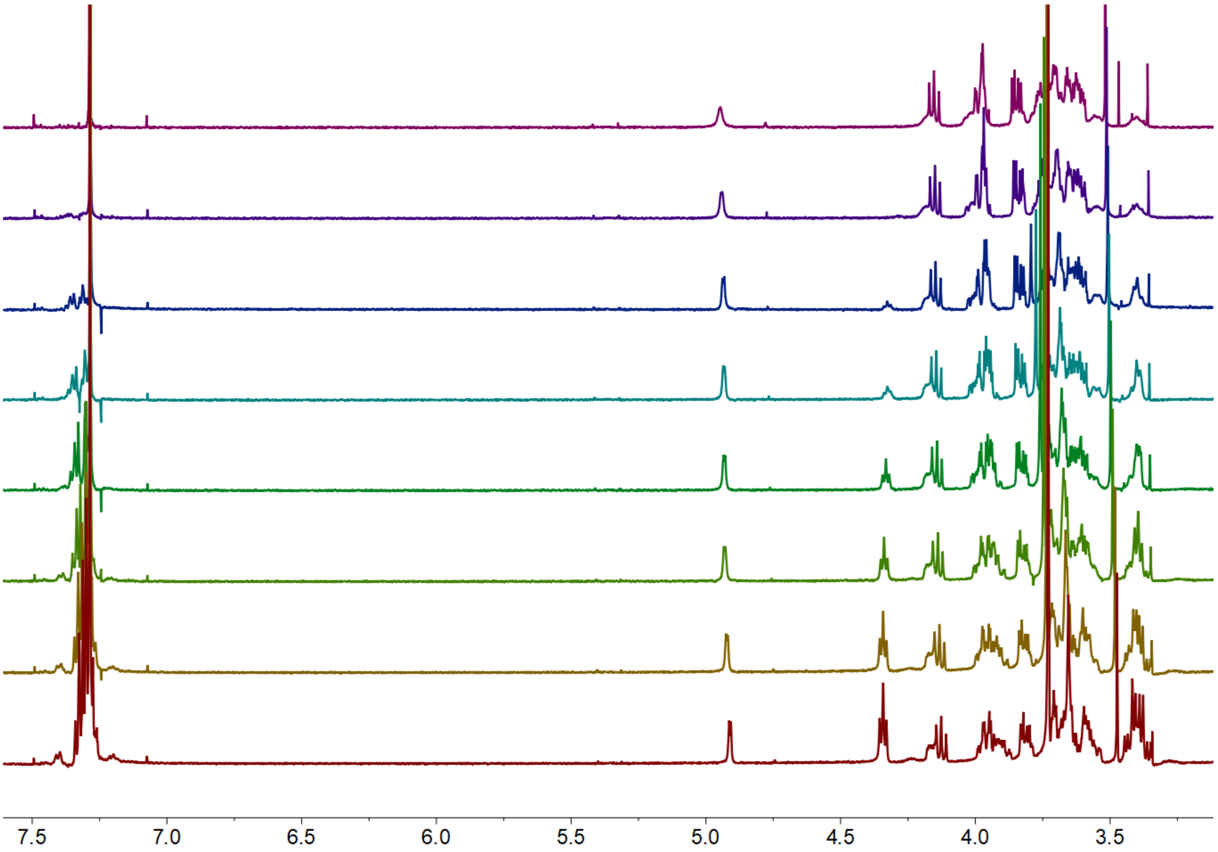

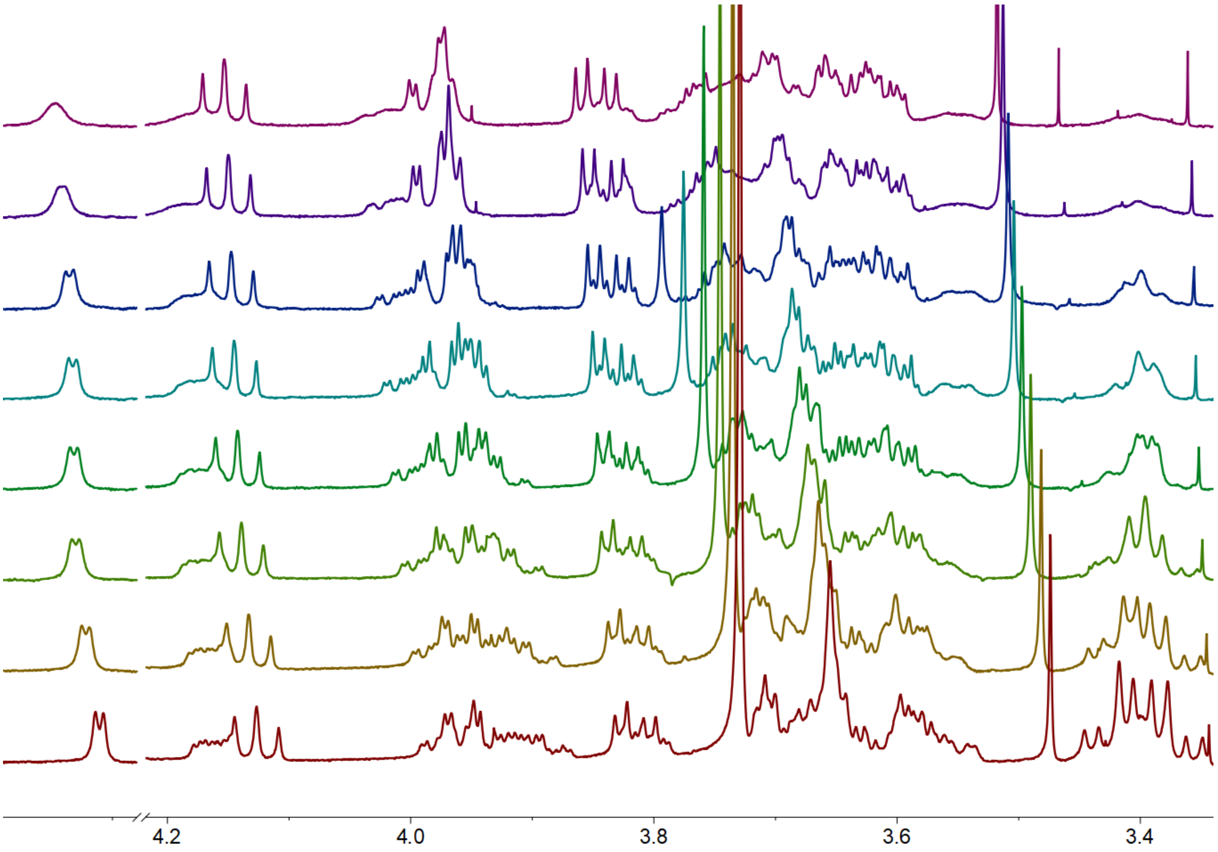

Summary of chemical shift changes:

Titration	4.94	Δppm	3.98	Δppm	3.96	Δppm	3.62	Δppm
0	4.9478	0.0000	3.9834	0.0000	3.9655	0.0000	3.6224	0.0000
1	4.941	0.0068	3.9759	0.0075	3.958	0.0075	3.6171	0.0053
2	4.9355	0.0123	3.9702	0.0132	3.9535	0.0120	3.6134	0.0090
3	4.9328	0.0150	3.9663	0.0171	3.9493	0.0162	3.6110	0.0114
4	4.9316	0.0162	3.9599	0.0235	3.9451	0.0204	3.6082	0.0142
5	4.9301	0.0177	3.9547	0.0287	3.9417	0.0238	3.6059	0.0165
6	4.9223	0.0255	3.9508	0.0326	3.9385	0.0270	3.6017	0.0207
7	4.9138	0.0340	3.9491	0.0343	3.9371	0.0284	3.5985	0.0239

Plot of chemical shift change vs guest concentration:

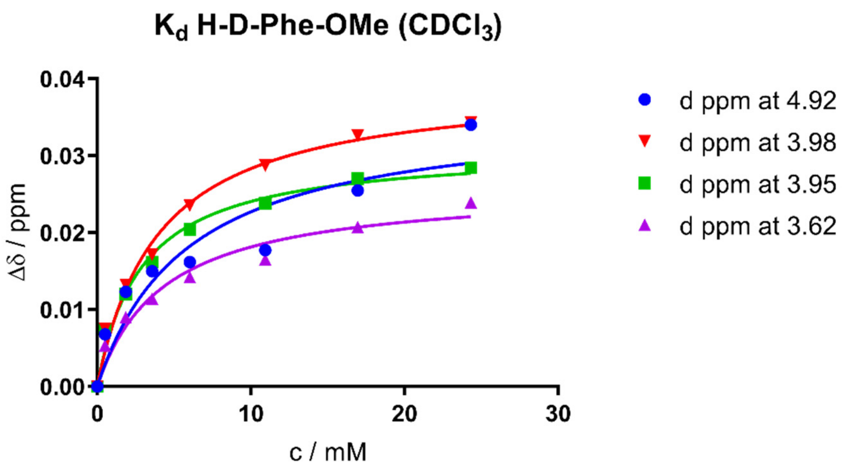

K_d values obtained by fitting the data for selected protons into the single-site specific binding model:

Kd	5.83	4.58	3.55	4.92
R squared	0.8759	0.9883	0.9833	0.9607

K_d = 4.72 ± 0.82 mM → K_a = 212 ± 37 M^{-1}

H-L-Pro-OMe x HCl to receptor 1:

^{1}H NMR spectra (top – full, bottom – enhanced view of the receptor signals in the spectra):

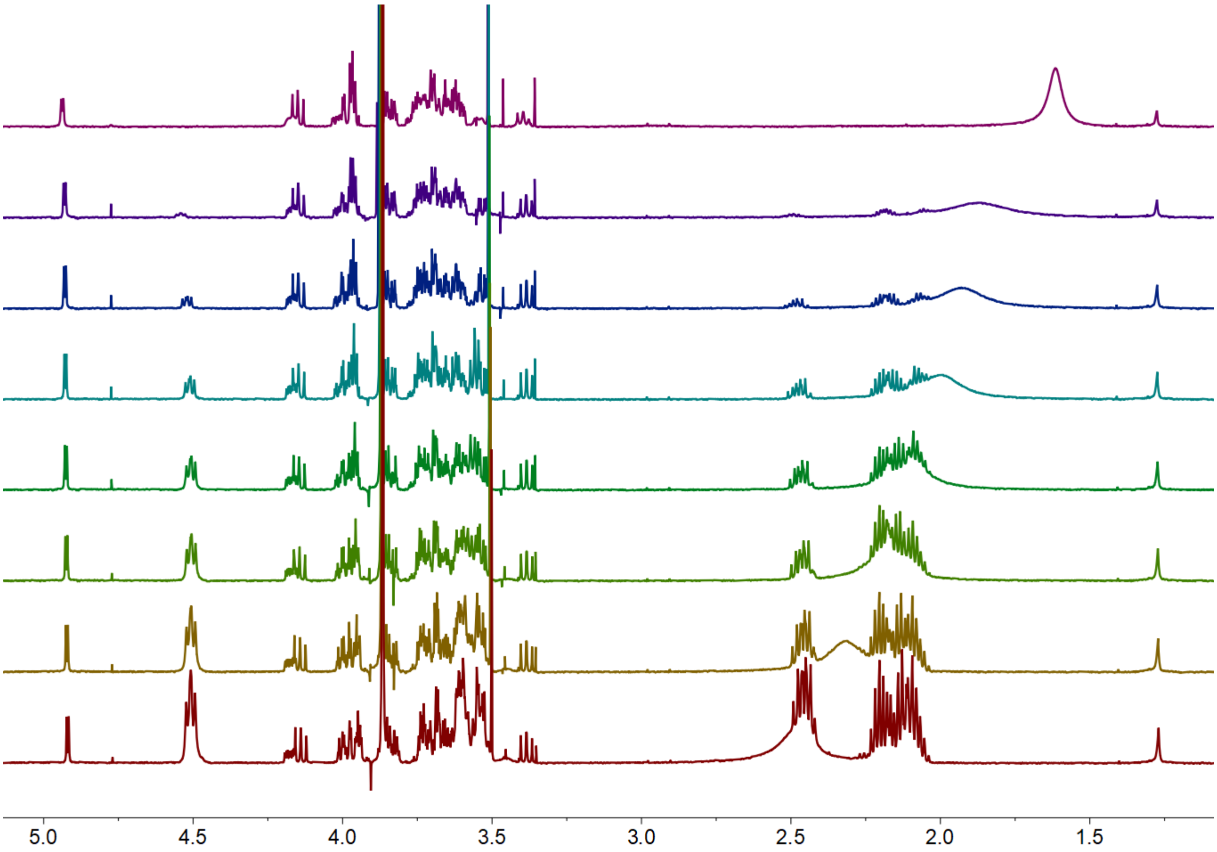

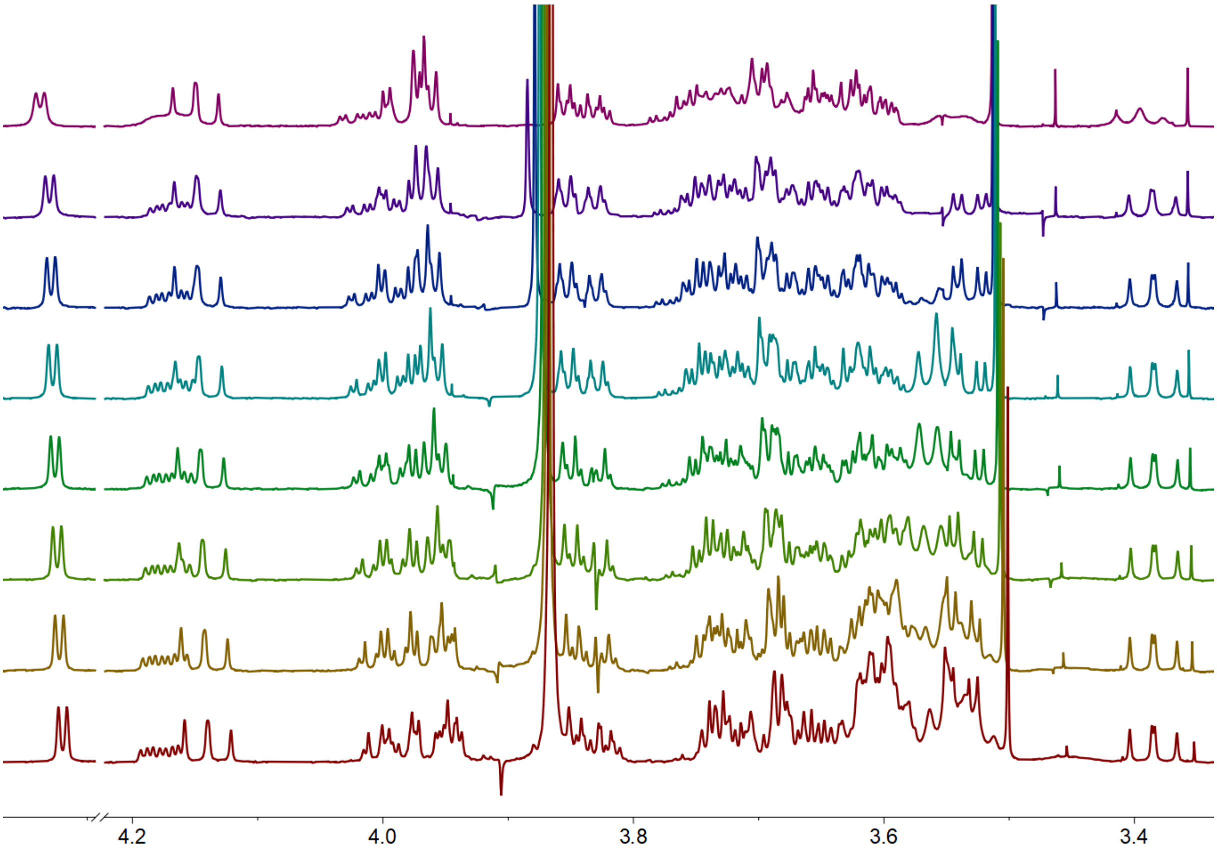

Summary of chemical shift changes:

Titration	4.93	Δppm	4	Δppm	3.84	Δppm	3.71	Δppm
0	4.9375	0.0000	3.9968	0.0000	3.8453	0.0000	3.7054	0.0000
1	4.9301	0.0074	4.0002	0.0034	3.8422	0.0031	3.7019	0.0035
2	4.9288	0.0087	4.0008	0.0040	3.8409	0.0044	3.7007	0.0047
3	4.9273	0.0102	4.001	0.0042	3.84	0.0053	3.6993	0.0061
4	4.9255	0.0120	4.0015	0.0047	3.8375	0.0078	3.697	0.0084
5	4.9238	0.0137	4.0019	0.0051	3.9362	0.0909	3.6949	0.0105
6	4.9215	0.0160	4.003	0.0062	3.8344	0.0109	3.6911	0.0143
7	4.9187	0.0188	4.0033	0.0065	3.8312	0.0141	3.6895	0.0159

Plot of chemical shift change vs guest concentration:

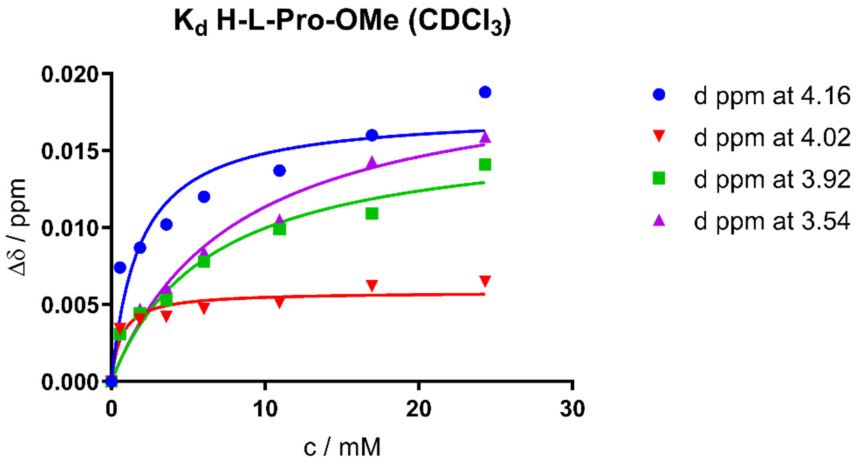

K_d values obtained by fitting the data for selected protons into the single-site specific binding model:

Kd	3.37	2.59	4.06	5.65
**R squared**	0.888	0.8916	0.9622	0.9546

K_d = 3.92 ± 1.13 mM → K_a = 255 ± 74 M^{-1}

H-D-Pro-OMe x HCl to receptor 1:

^{1}H NMR spectra (top – full, bottom – enhanced view of the receptor signals in the spectra):

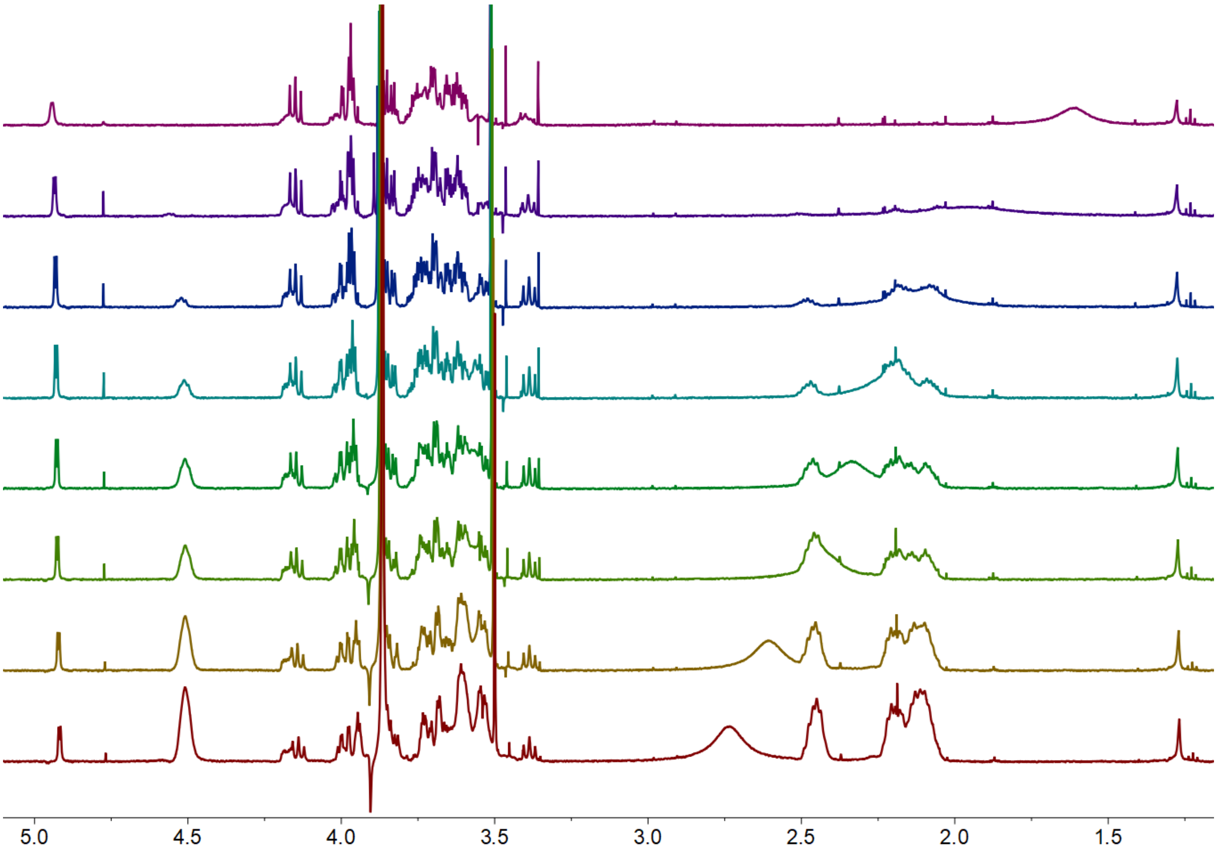

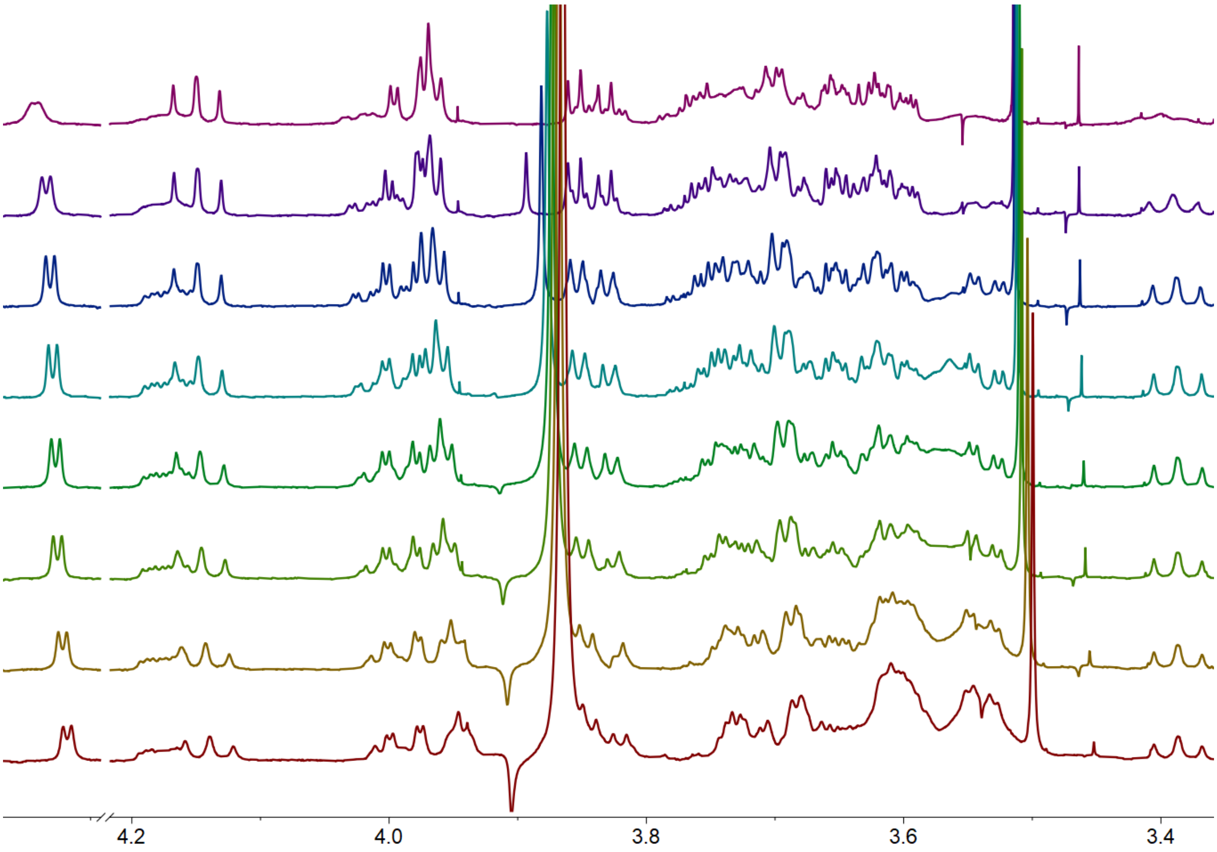

Summary of chemical shift changes:

Titration	4.94	Δppm	4.02	Δppm	3.84	Δppm	3.39	Δppm
0	4.9436	0.0000	4.0276	0.0000	3.8448	0.0000	3.3957	0.0000
1	4.9346	0.0090	4.0215	0.0061	3.8424	0.0024	3.3856	0.0101
2	4.9314	0.0122	4.0191	0.0085	3.8414	0.0034	3.3849	0.0108
3	4.9291	0.0145	4.0169	0.0107	3.84	0.0048	3.3845	0.0112
4	4.9271	0.0165	4.0153	0.0123	3.8392	0.0056	3.3844	0.0113
5	4.9256	0.0180	4.0135	0.0141	3.8369	0.0079	3.3842	0.0115
6	4.9219	0.0217	4.0106	0.0170	3.8339	0.0109	3.3841	0.0116
7	4.9177	0.0259	4.0075	0.0201	3.8323	0.0125	3.3840	0.0117

Plot of chemical shift change vs guest concentration:

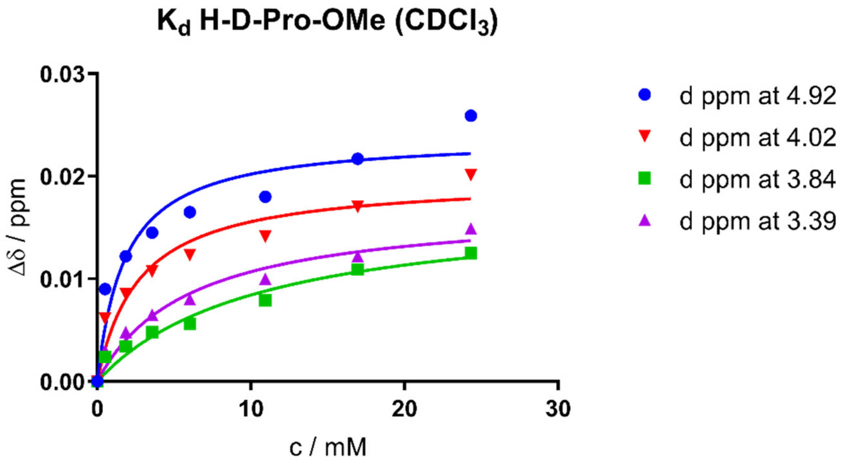

K_d values obtained by fitting the data for selected protons into the single-site specific binding model:

Kd	3.41	5.8	8.69	6.82
**R squared**	0.929	0.9592	0.9612	0.9984

K_d = 6.18 ± 1.91 mM → K_a = 162 ± 50 M^{-1}

H-L-Thr-OMe x HCl to receptor 1:

^{1}H NMR spectra (top – full, bottom – enhanced view of the receptor signals in the spectra):

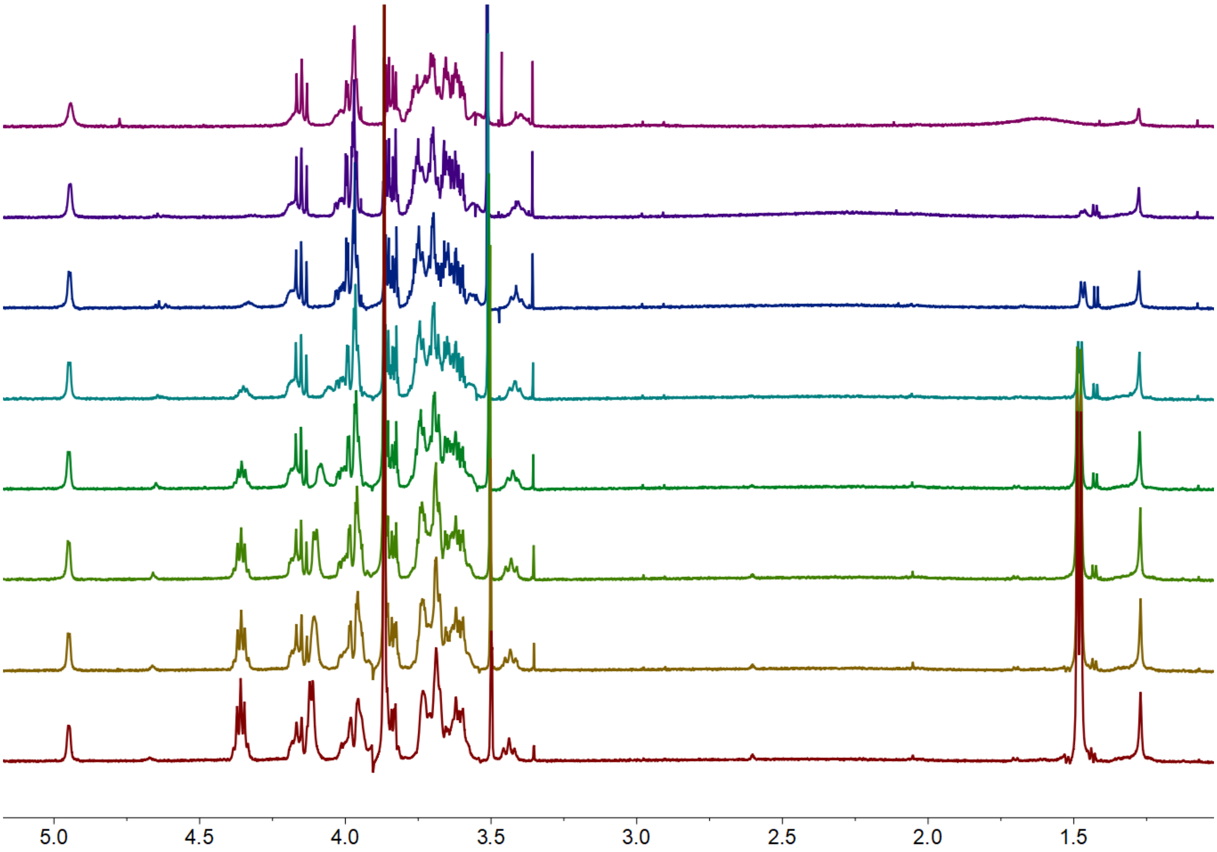

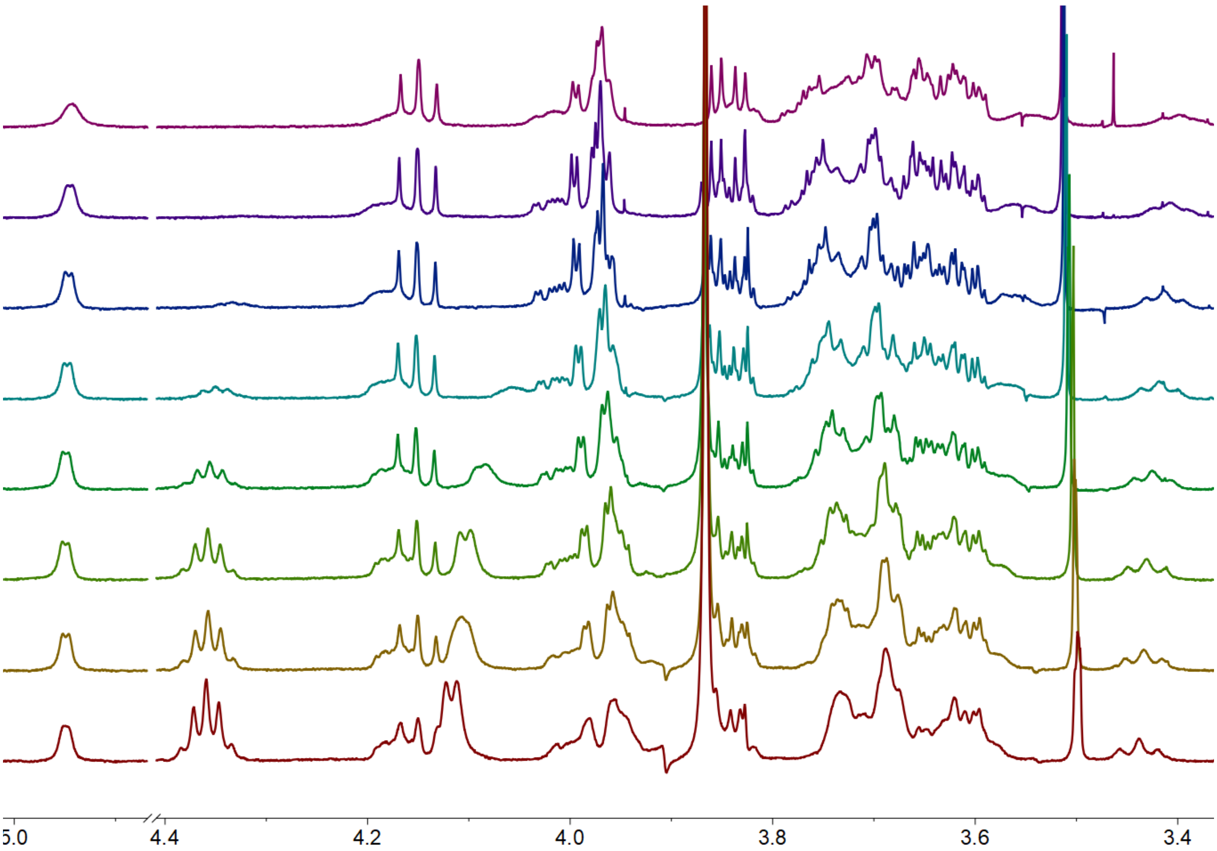

Summary of chemical shift changes:

Titration	4.94	Δppm	4.13	Δppm	3.82	Δppm	3.76	Δppm
0	4.9424	0.0000	4.1393	0.0000	3.8266	0.0000	3.7609	0.0000
1	4.9453	0.0029	4.1411	0.0018	3.8303	0.0037	3.7559	0.0050
2	4.9466	0.0042	4.1418	0.0025	3.8331	0.0065	3.7516	0.0093
3	4.948	0.0056	4.1423	0.0030	3.8351	0.0085	3.7485	0.0124
4	4.9488	0.0064	4.1426	0.0033	3.8362	0.0096	3.7447	0.0162
5	4.9492	0.0068	4.1428	0.0035	3.8369	0.0103	3.7404	0.0205
6	4.9495	0.0071	4.1429	0.0036	3.837	0.0104	3.7392	0.0217
7	4.9496	0.0072	4.1429	0.0036	3.8371	0.0105	3.7388	0.0221

Plot of chemical shift change vs guest concentration:

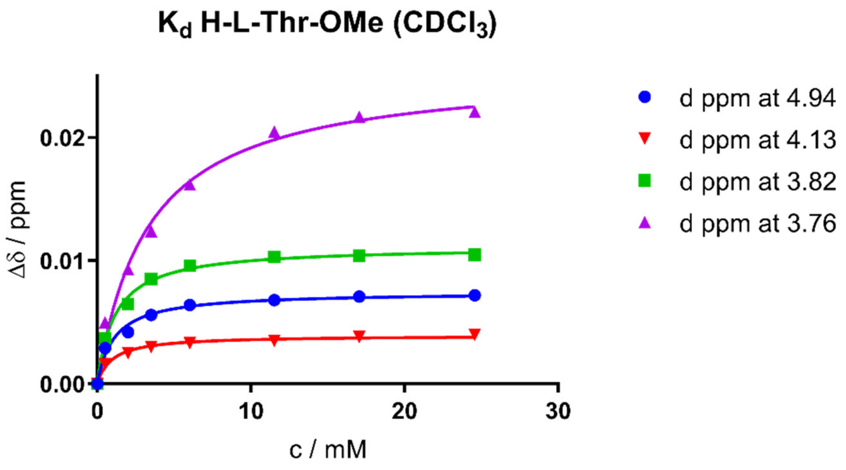

K_d values obtained by fitting the data for selected protons into the single-site specific binding model:

Kd	1.21	1.06	1.36	2.24
**R squared**	0.9836	0.986	0.9944	0.983

K_d = 1.47 ± 0.46 mM → K_a = 680 ± 213 M^{-1}

H-D-Thr-OMe x HCl to receptor 1:

^{1}H NMR spectra (top – full, bottom – enhanced view of the receptor signals in the spectra):

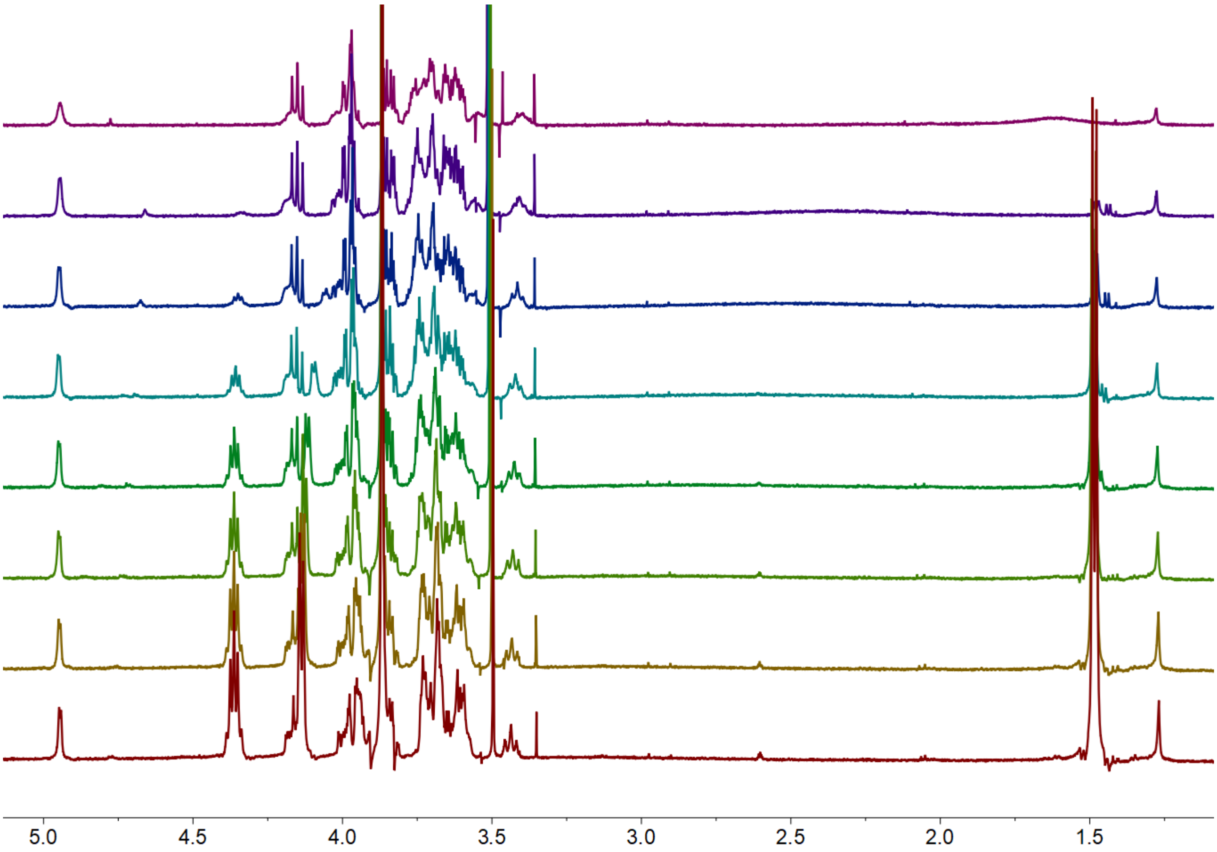

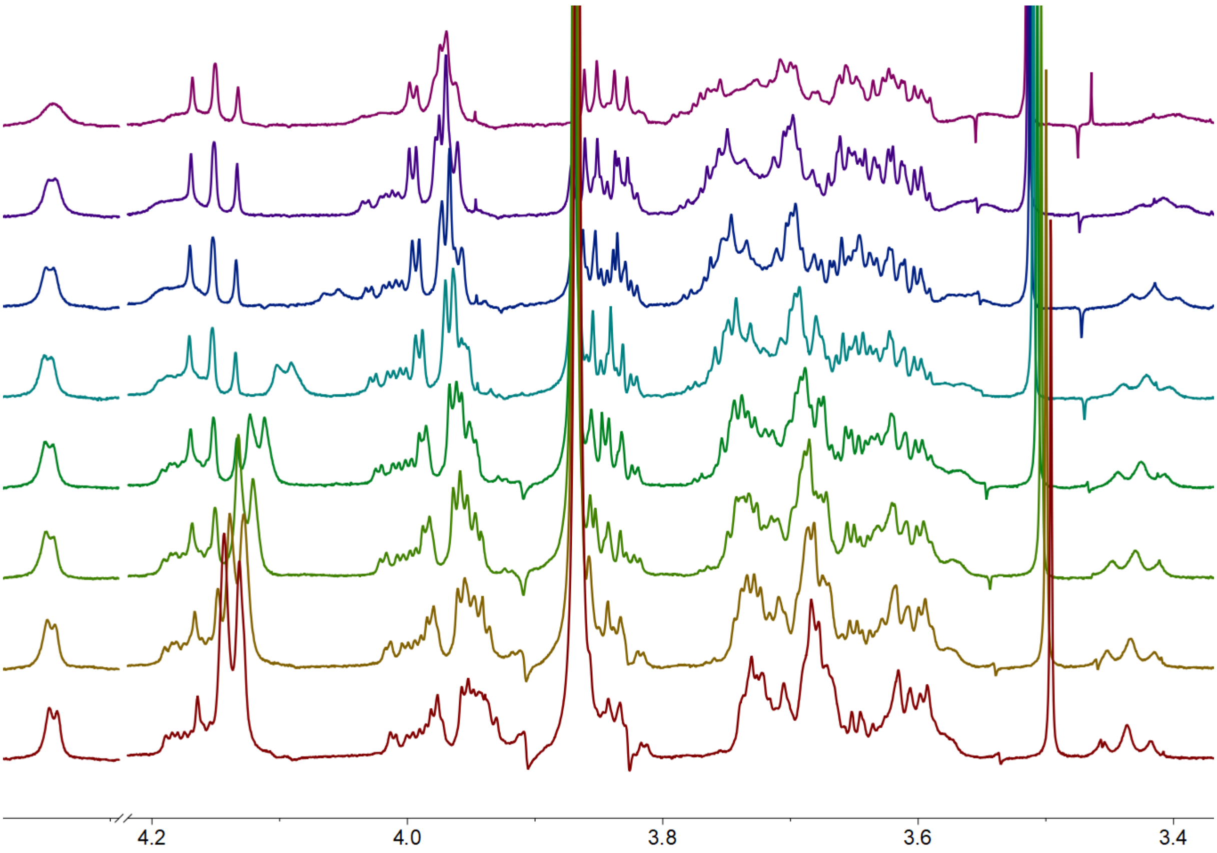

Summary of chemical shift changes:

Titration	4.19	Δppm	3.83	Δppm	3.7	Δppm	3.39	Δppm
0	4.1923	0.0000	3.8367	0.0000	3.7074	0.0000	3.3979	0.0000
1	4.1899	0.0024	3.8373	0.0006	3.7016	0.0058	3.4081	0.0102
2	4.1863	0.0060	3.8389	0.0022	3.6994	0.0080	3.4149	0.0170
3	4.1857	0.0066	3.8407	0.0040	3.6968	0.0106	3.4208	0.0229
4	4.1842	0.0081	3.8418	0.0051	3.6928	0.0146	3.4254	0.0275
5	4.1837	0.0086	3.8424	0.0057	3.6895	0.0179	3.4302	0.0323
6	4.1826	0.0097	3.8427	0.0060	3.6864	0.0210	3.4337	0.0358
7	4.182	0.0103	3.8428	0.0061	3.6843	0.0231	3.4358	0.0379

Plot of chemical shift change vs guest concentration:

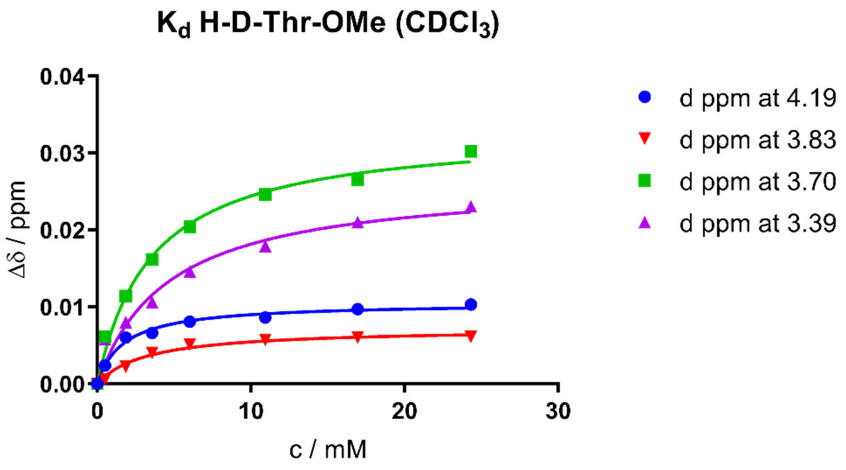

K_d values obtained by fitting the data for selected protons into the single-site specific binding model:

Kd	2	3.35	3.66	4.57
**R squared**	0.9906	0.9823	0.9904	0.9711

K_d = 3.40 ± 0.92 mM → K_a = 295 ± 80 M^{-1}

H-L-Val-OMe x HCl to receptor 1:

^{1}H NMR spectra (top – full, bottom – enhanced view of the receptor signals in the spectra):

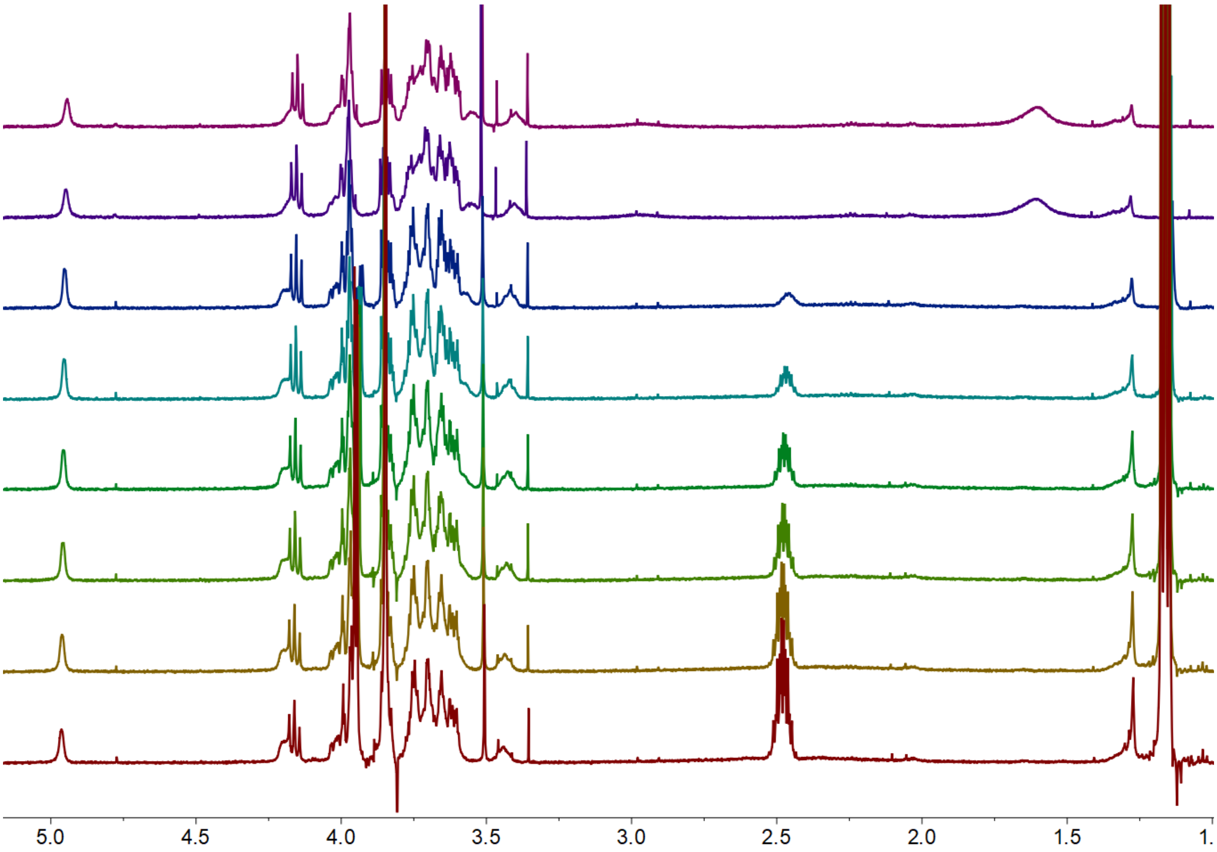

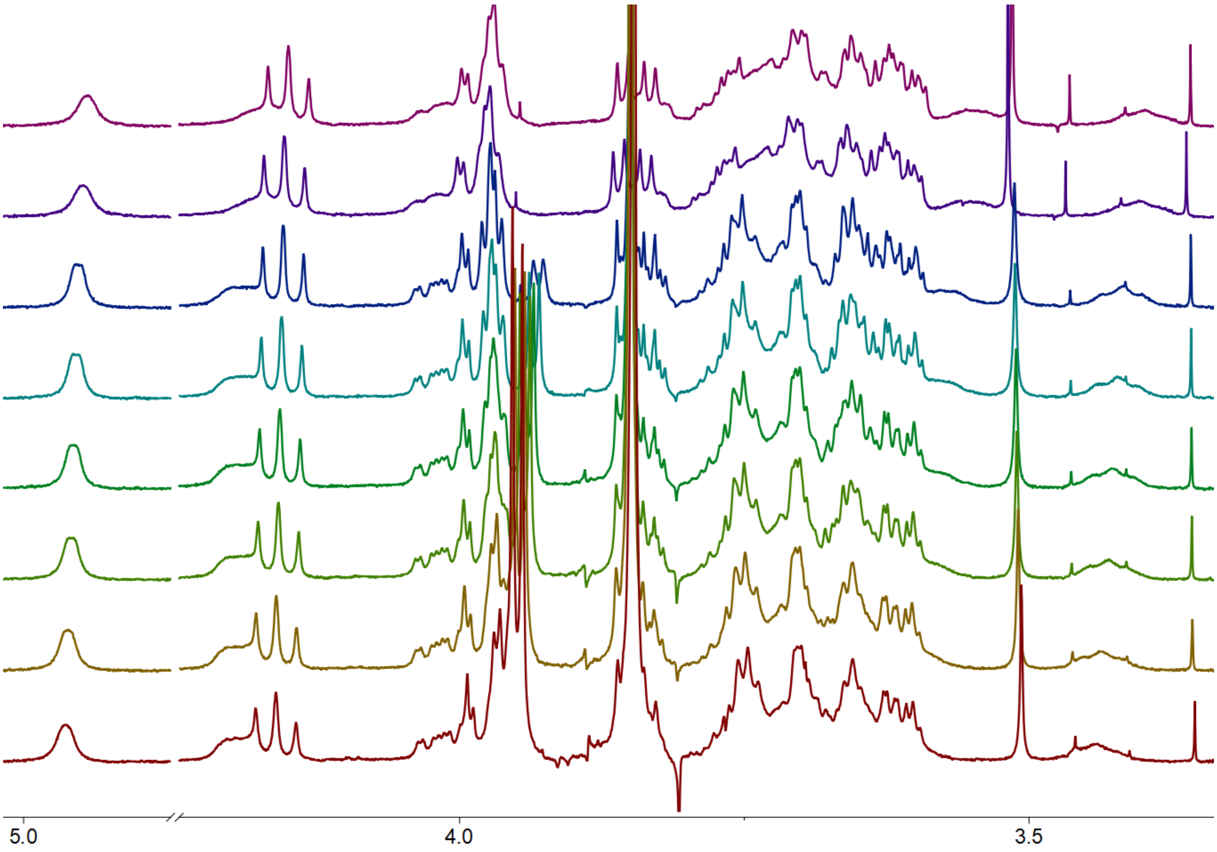

Summary of chemical shift changes:

Titration	4.15	Δppm	4.01	Δppm	3.86	Δppm	3.59	Δppm
0	4.1502	0.0000	4.0064	0.0000	3.8695	0.0000	3.59	0.0000
1	4.154	0.0038	4.0022	0.0042	3.8652	0.0043	3.5927	0.0027
2	4.1552	0.0050	3.998	0.0084	3.862	0.0075	3.5933	0.0033
3	4.1563	0.0061	3.9975	0.0089	3.8617	0.0078	3.5938	0.0038
4	4.1575	0.0073	3.9969	0.0095	3.8615	0.0080	3.5943	0.0043
5	4.1584	0.0082	3.9963	0.0101	3.8613	0.0082	3.5951	0.0051
6	4.1594	0.0092	3.9957	0.0107	3.8612	0.0083	3.5955	0.0055
7	4.1601	0.0099	3.9937	0.0127	3.8609	0.0086	3.5959	0.0059

Plot of chemical shift change vs guest concentration:

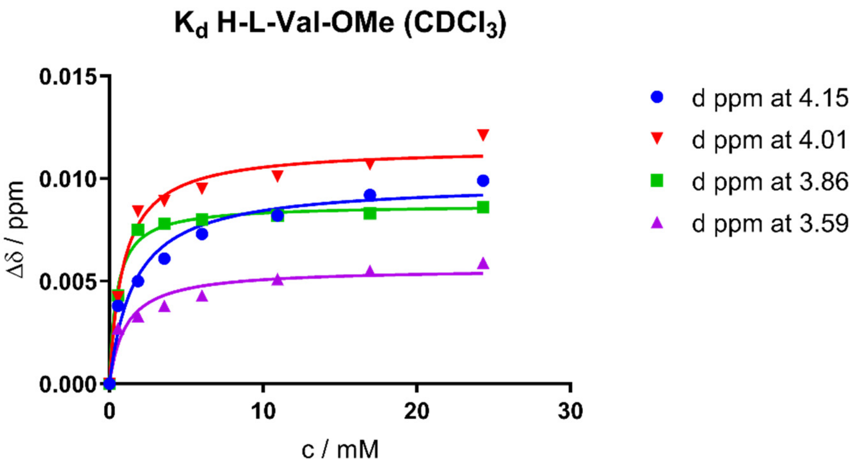

K_d values obtained by fitting the data for selected protons into the single-site specific binding model:

Kd	1.44	0.95	0.66	1.05
R squared	0.9503	0.9705	0.9951	0.9303

K_d = 1.03 ± 0.28 mM → K_a = 977 ± 266 M^{-1}

H-D-Val-OMe x HCl to receptor 1:

^{1}H NMR spectra (top – full, bottom – enhanced view of the receptor signals in the spectra):

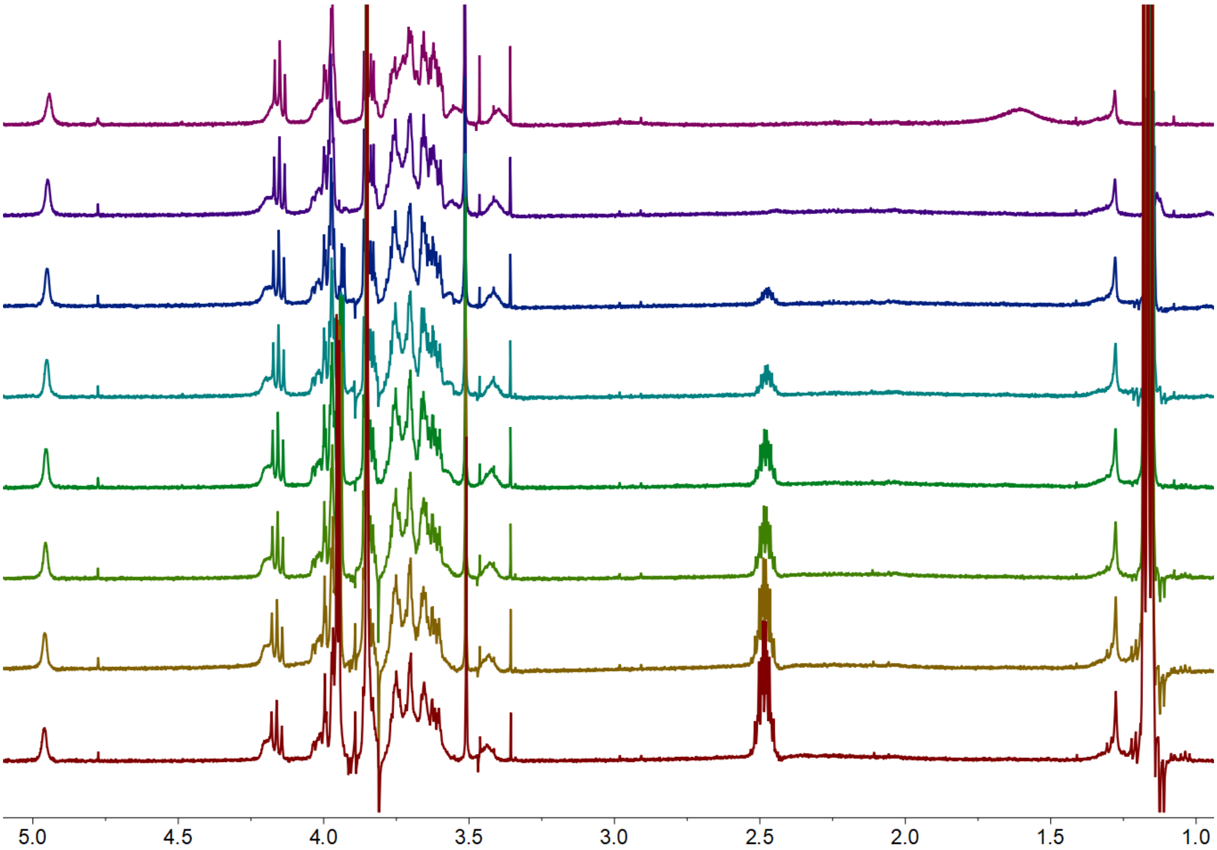

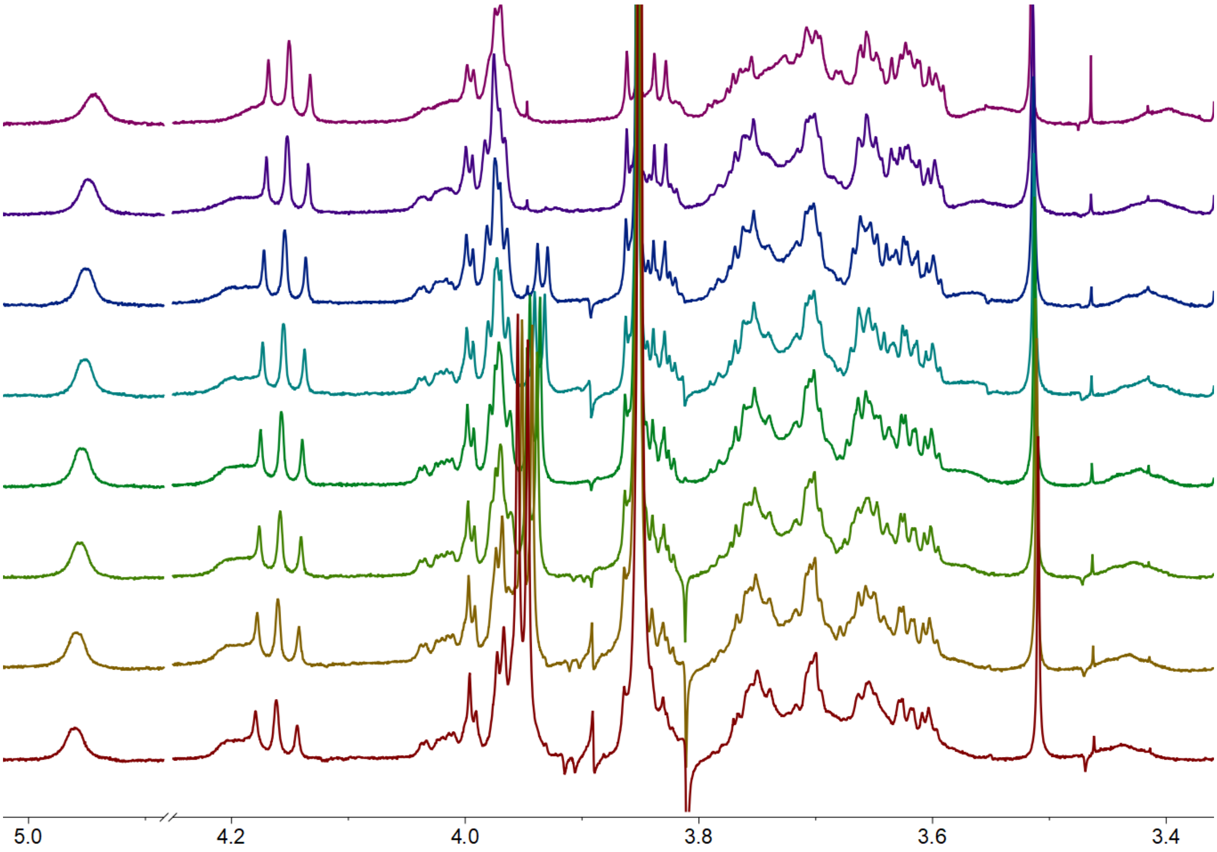

Summary of chemical shift changes:

Titration	4.15	Δppm	4	Δppm	3.86	Δppm	3.59	Δppm
0	4.1508	0.0000	4.0006	0.0000	3.8616	0.0000	3.5973	0.0000
1	4.1522	0.0014	3.9994	0.0012	3.8619	0.0003	3.5983	0.0010
2	4.154	0.0032	3.9988	0.0018	3.8624	0.0008	3.5992	0.0019
3	4.155	0.0042	3.9985	0.0021	3.8627	0.0011	3.5999	0.0026
4	4.1564	0.0056	3.9978	0.0028	3.8631	0.0015	3.6009	0.0036
5	4.1573	0.0065	3.9973	0.0033	3.8624	0.0008	3.6016	0.0043
6	4.1588	0.0080	3.9968	0.0038	3.8637	0.0021	3.6025	0.0052
7	4.1598	0.0090	3.9962	0.0044	3.8639	0.0023	3.6032	0.0059

Plot of chemical shift change vs guest concentration:

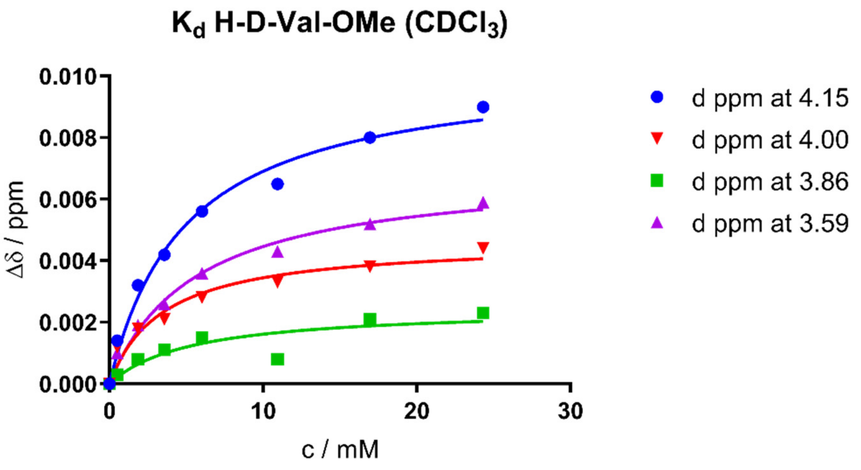

K_d values obtained by fitting the data for selected protons into the single-site specific binding model:

Kd	5.68	3.57	5.11	5.54
R squared	0.9756	0.9374	0.9867	0.9699

K_d = 4.98 ± 0.84 mM → K_a = 201 ± 34 M^{-1}

H-L-Asn-OMe x HCl to receptor 1:

^{1}H NMR spectra (top – full, bottom – enhanced view of the receptor signals in the spectra):

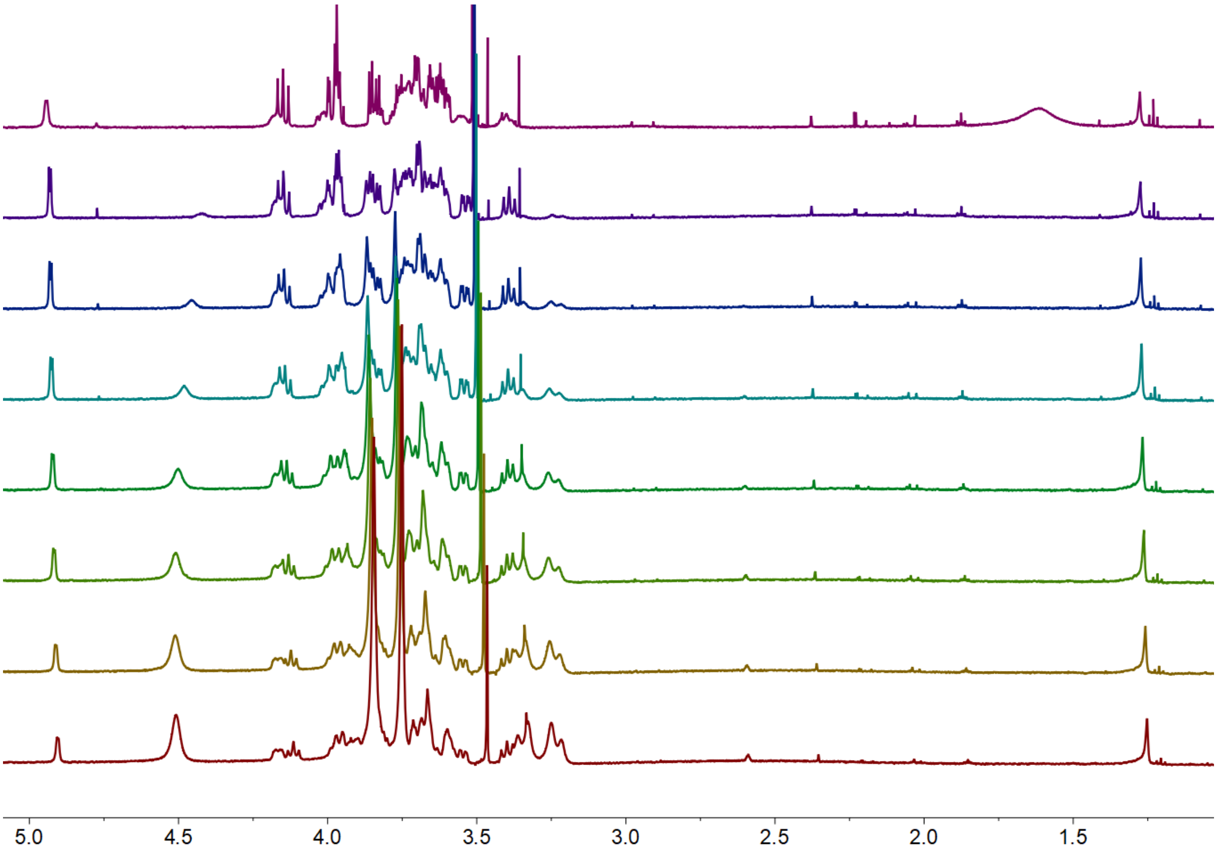

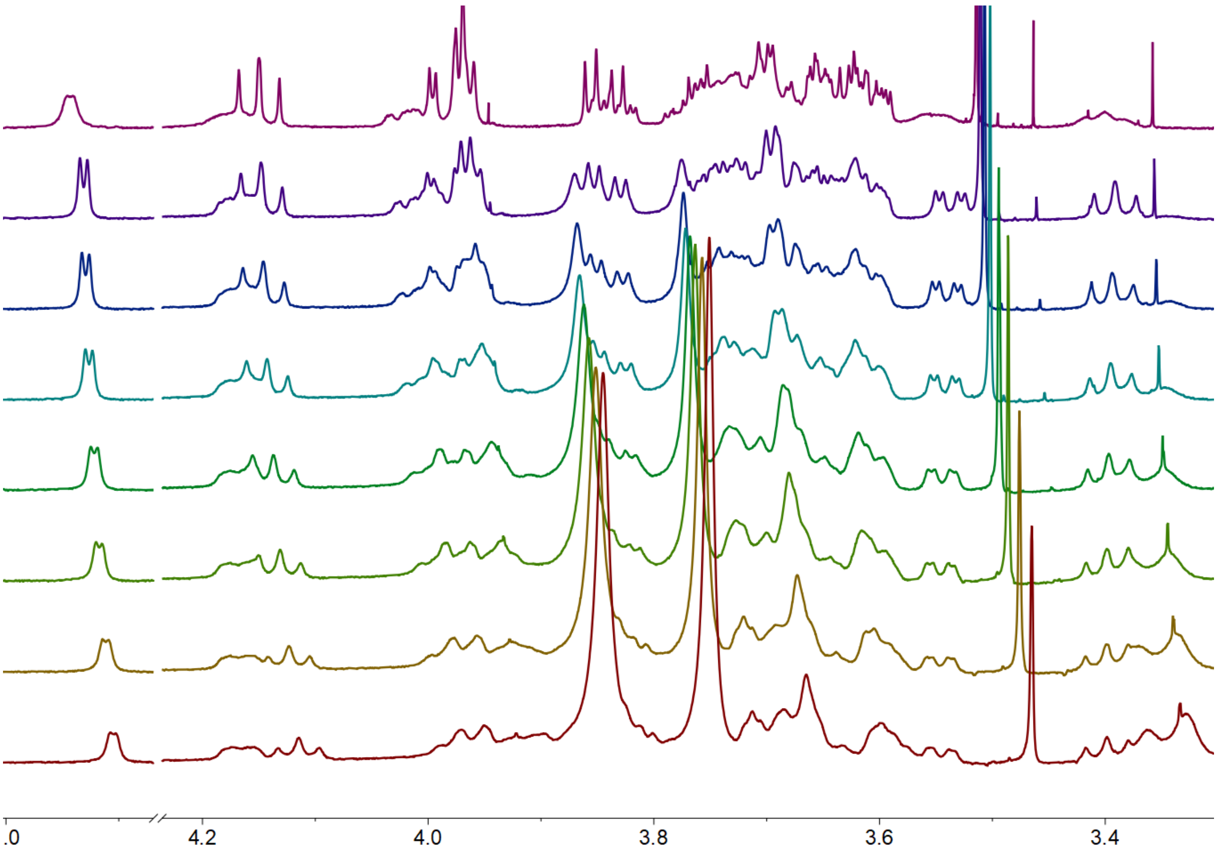

Summary of chemical shift changes:

Titration	4.94	Δppm	4.02	Δppm	3.53	Δppm	3.38	Δppm
0	4.9425	0.0000	4.0277	0.0000	3.5323	0.0000	3.3854	0.0000
1	4.9309	0.0116	4.0207	0.0070	3.5377	0.0054	3.3913	0.0059
2	4.9292	0.0133	4.0185	0.0092	3.5406	0.0083	3.3936	0.0082
3	4.9262	0.0163	4.0151	0.0126	3.5425	0.0102	3.3951	0.0097
4	4.9216	0.0209	4.0114	0.0163	3.545	0.0127	3.3970	0.0116
5	4.9161	0.0264	4.0075	0.0202	3.5458	0.0135	3.3977	0.0123
6	4.9123	0.0302	4.0029	0.0248	3.5461	0.0138	3.3982	0.0128
7	4.9079	0.0346	3.9975	0.0302	3.5468	0.0145	3.3987	0.0133

Plot of chemical shift change vs guest concentration:

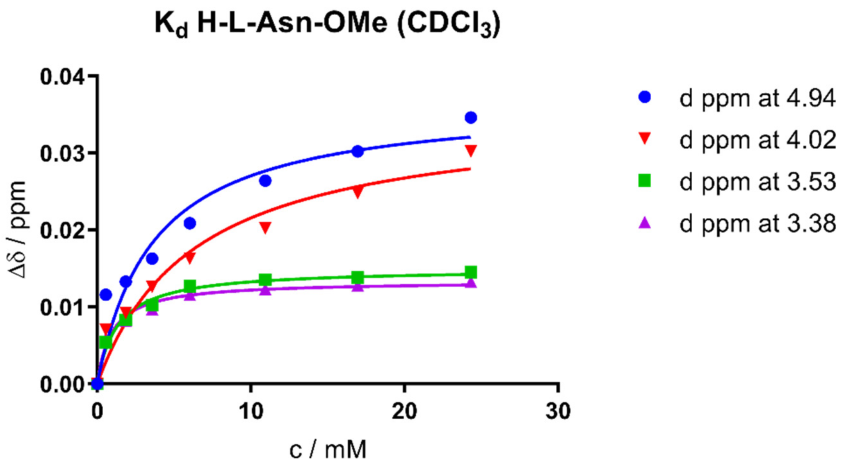

K_d values obtained by fitting the data for selected protons into the single-site specific binding model:

Kd	3.77	4.45	2.29	1.92
R squared	0.9237	0.9522	0.9835	0.9768

K_d = 3.11 ± 1.04 mM → K_a = 322 ± 108 M^{-1}

H-D-Asn-OMe x HCl to receptor 1:

^{1}H NMR spectra (top – full, bottom – enhanced view of the receptor signals in the spectra):

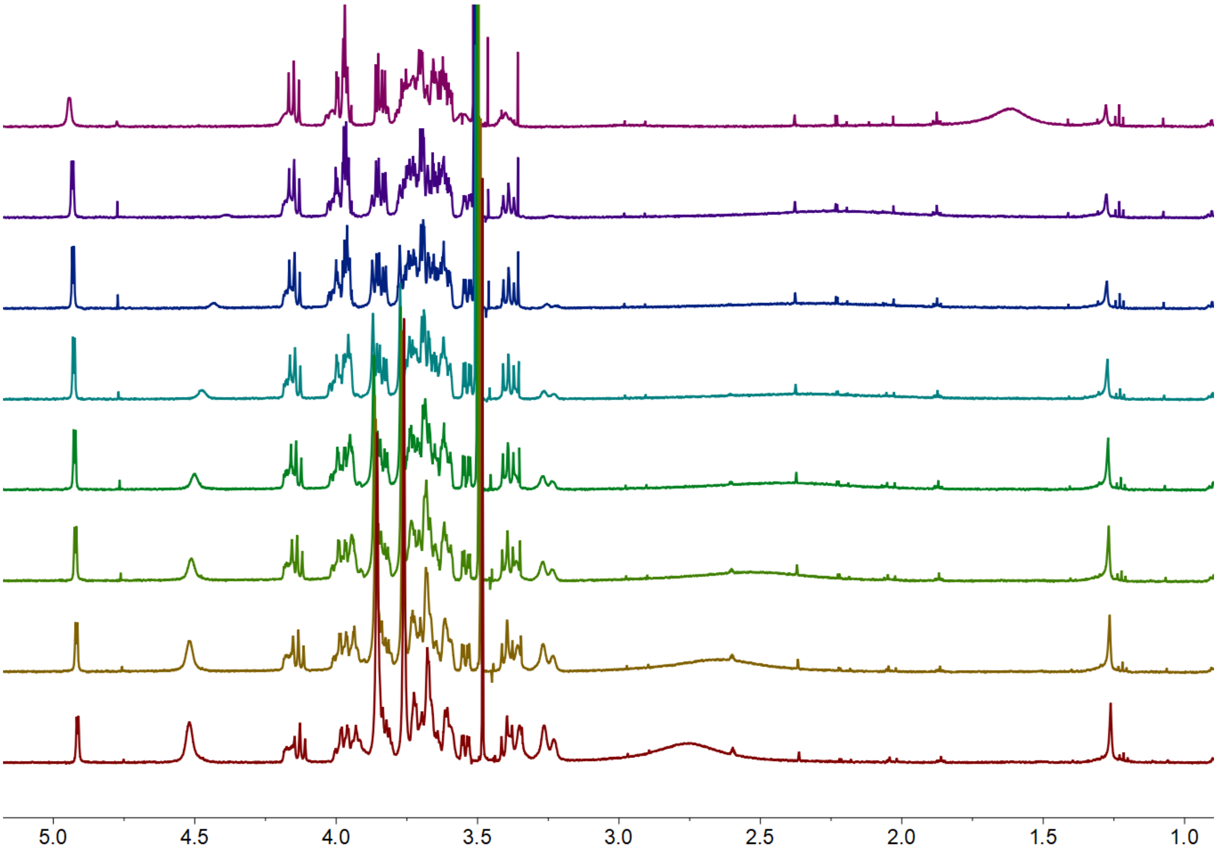

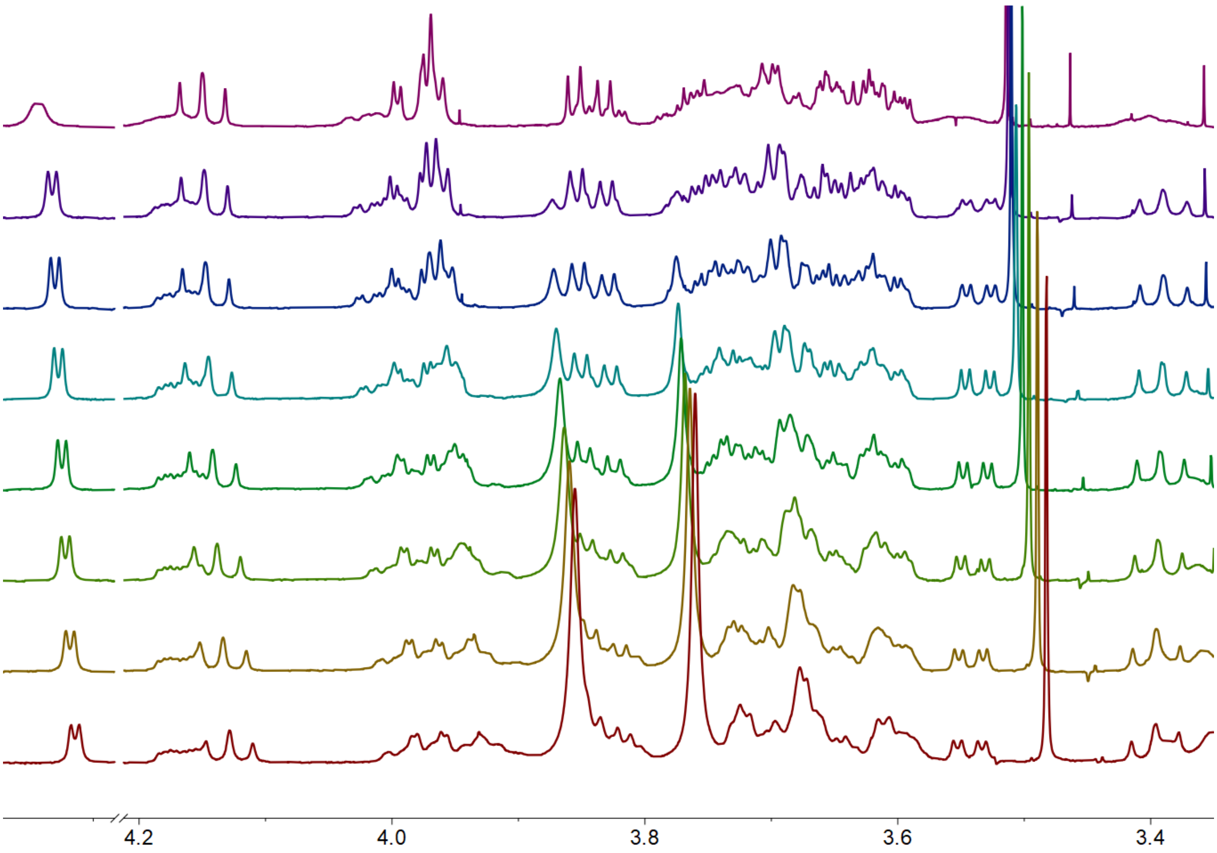

Summary of chemical shift changes:

Titration	4.94	Δppm	4.02	Δppm	3.53	Δppm	3.38	Δppm
0	4.944	0.0000	4.0277	0.0000	3.5312	0.0000	3.3826	0.0000
1	4.9324	0.0116	4.0207	0.0070	3.5354	0.0042	3.3881	0.0055
2	4.9303	0.0137	4.0185	0.0092	3.5363	0.0051	3.3897	0.0071
3	4.9273	0.0167	4.0151	0.0126	3.5374	0.0062	3.3905	0.0079
4	4.9245	0.0195	4.0114	0.0163	3.5396	0.0084	3.3922	0.0096
5	4.9222	0.0218	4.0075	0.0202	3.5409	0.0097	3.3941	0.0115
6	4.9183	0.0257	4.0029	0.0248	3.5423	0.0111	3.3954	0.0128
7	4.914	0.0300	3.9975	0.0302	3.5431	0.0119	3.3960	0.0134

Plot of chemical shift change vs guest concentration:

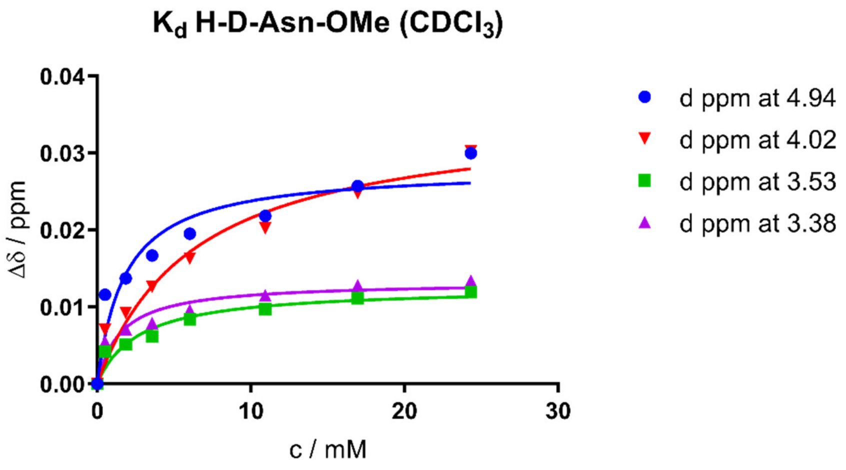

K_d values obtained by fitting the data for selected protons into the single-site specific binding model:

Kd	3.18	4.66	2.97	2.81
**R squared**	0.8999	0.9522	0.9408	0.9328

K_d = 3.41 ± 0.74 mM → K_a = 293 ± 64 M^{-1}

H-L-Leu-OMe x HCl to receptor 1:

^{1}H NMR spectra (top – full, bottom – enhanced view of the receptor signals in the spectra):

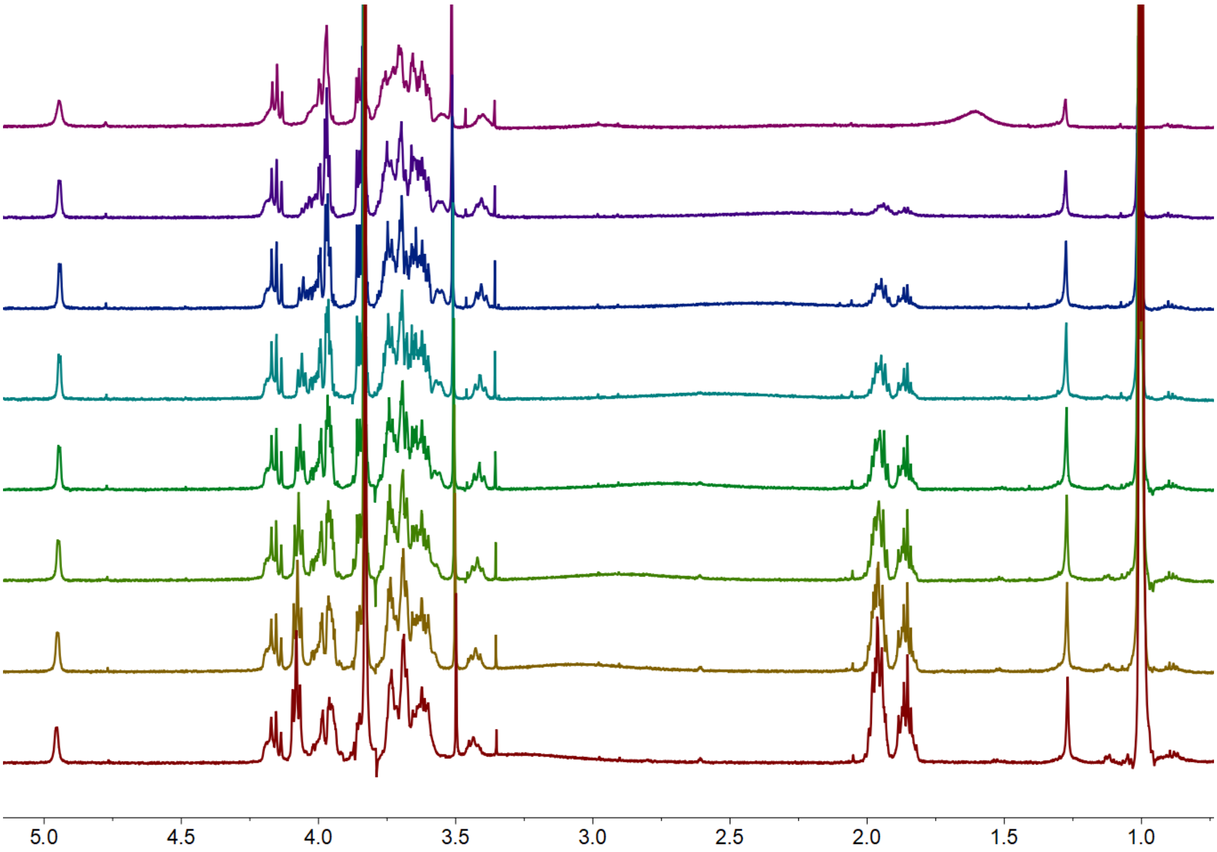

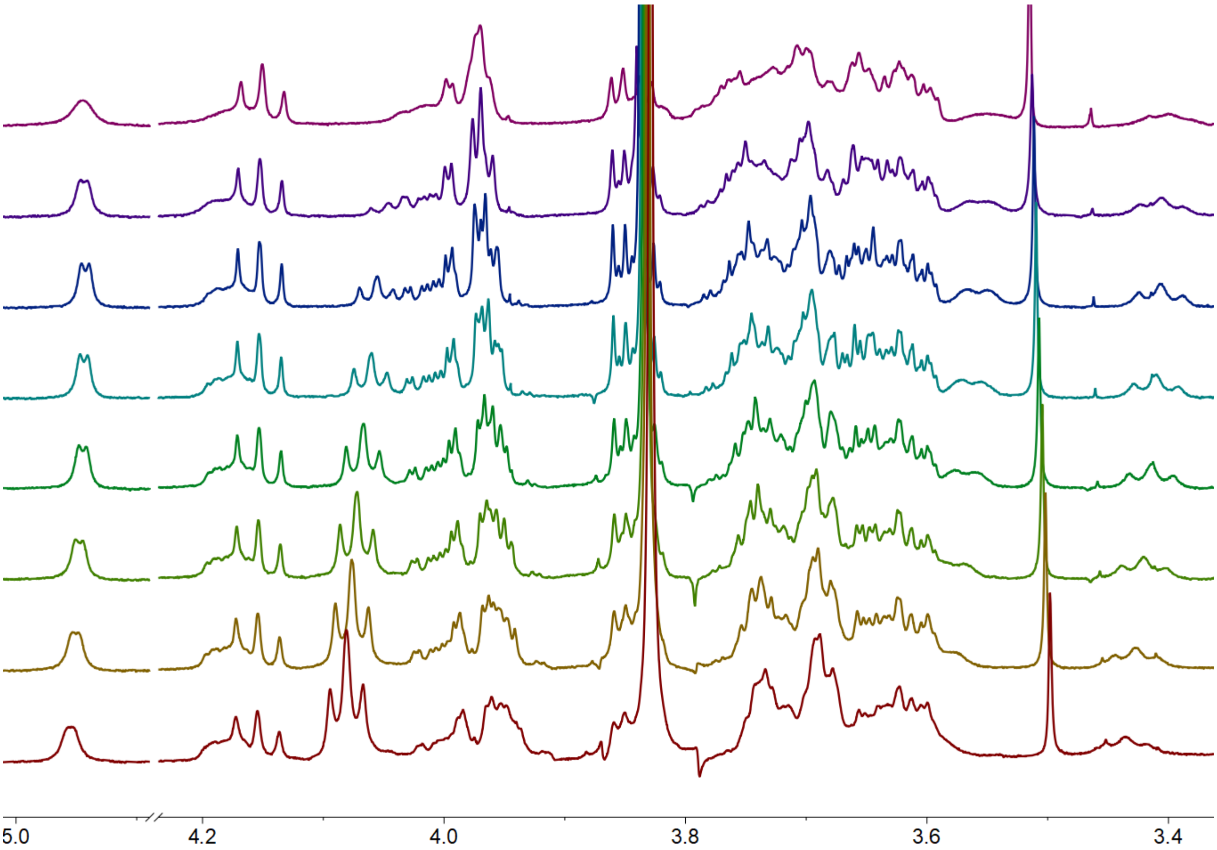

Summary of chemical shift changes:

Titration	4.15	Δppm	3.97	Δppm	3.77	Δppm	3.63	Δppm
0	4.1504	0.0000	3.9773	0.0000	3.771	0.0000	3.6256	0.0000
1	4.1524	0.0020	3.9724	0.0049	3.7661	0.0049	3.6300	0.0044
2	4.1528	0.0024	3.9706	0.0067	3.7633	0.0077	3.6311	0.0055
3	4.1532	0.0028	3.97	0.0073	3.7615	0.0095	3.6315	0.0059
4	4.1533	0.0029	3.9694	0.0079	3.7588	0.0122	3.6320	0.0064
5	4.1538	0.0034	3.9679	0.0094	3.7567	0.0143	3.6327	0.0071
6	4.1542	0.0038	3.9663	0.0110	3.7545	0.0165	3.6329	0.0073
7	4.1546	0.0042	3.964	0.0133	3.7522	0.0188	3.6330	0.0074

Plot of chemical shift change vs guest concentration:

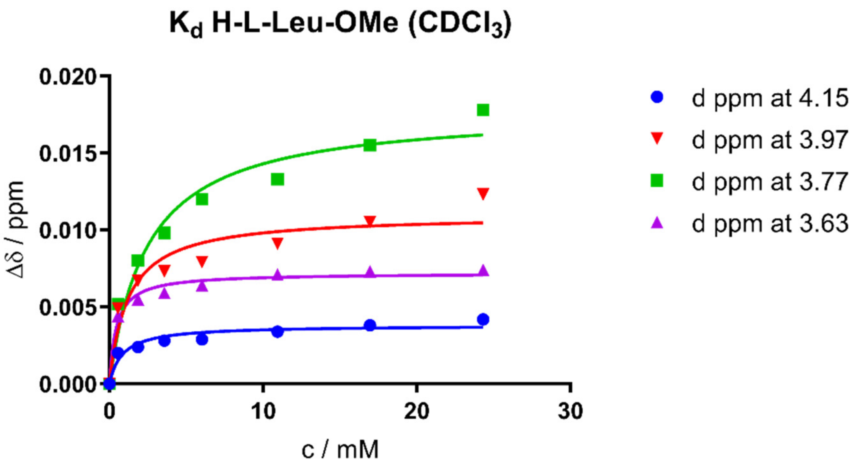

K_d values obtained by fitting the data for selected protons into the single-site specific binding model:

Kd	0.94	1.4	1.91	0.53
R squared	0.9155	0.8859	0.9665	0.9755

K_d = 1.20 ± 0.51 mM → K_a = 833 ± 354 M^{-1}

H-D-Leu-OMe x HCl to receptor 1:

^{1}H NMR spectra (top – full, bottom – enhanced view of the receptor signals in the spectra):

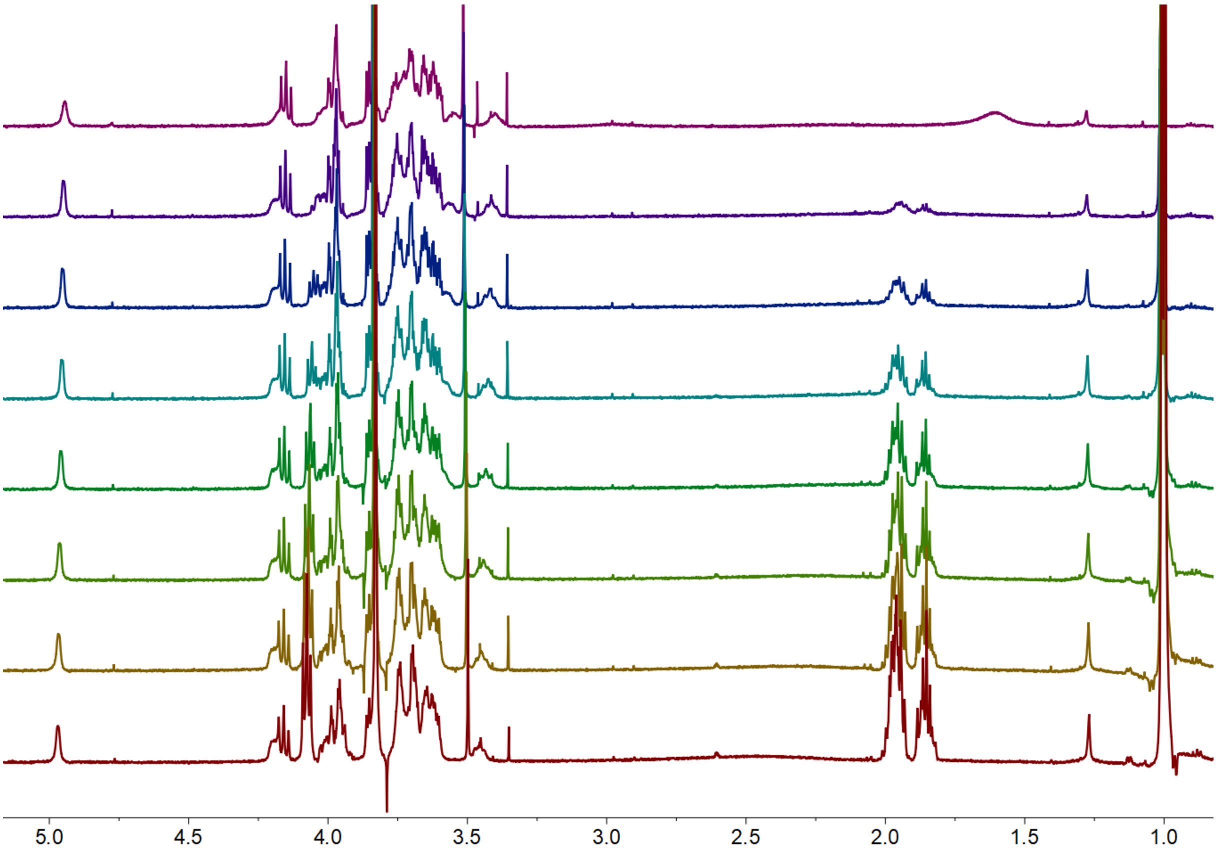

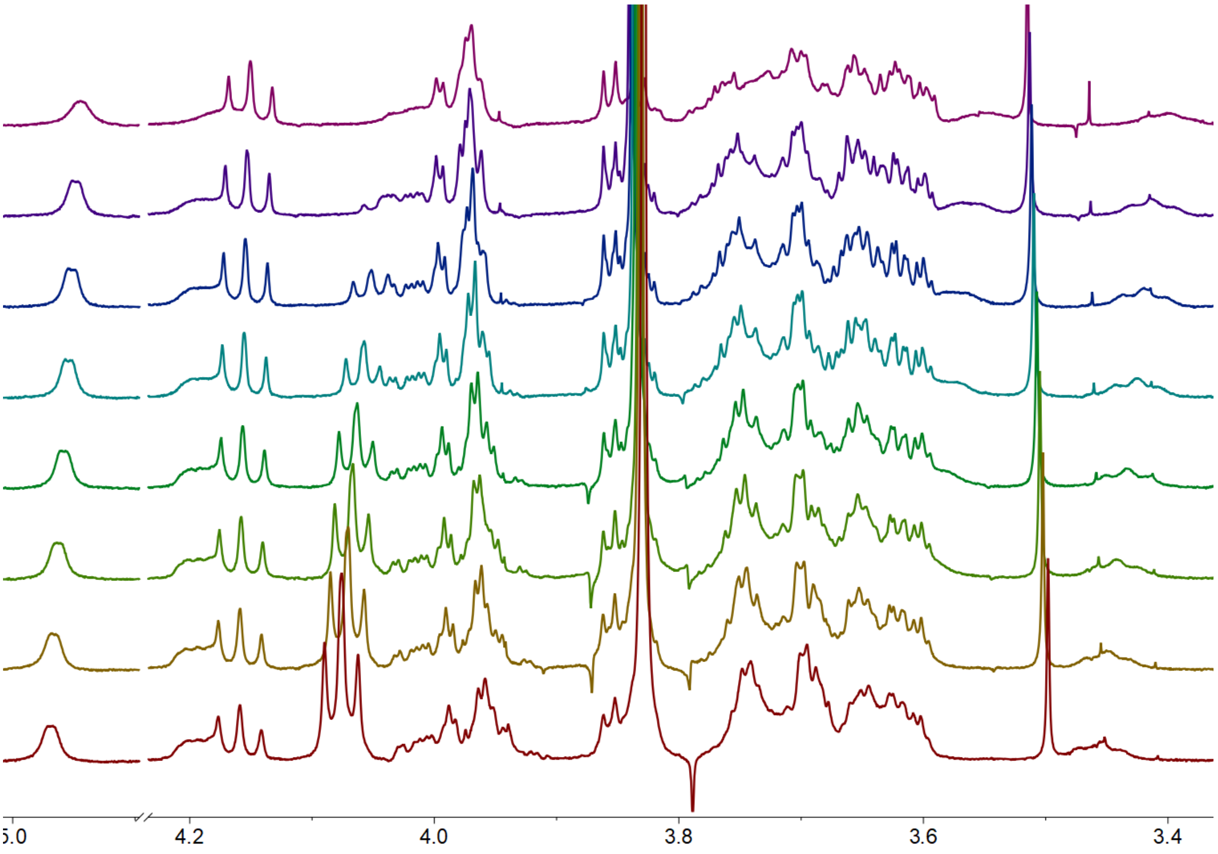

Summary of chemical shift changes:

Titration	4.94	Δppm	4.15	Δppm	3.97	Δppm	3.77	Δppm
0	4.9445	0.0000	4.1502	0.0000	3.9736	0.0000	3.7708	0.0000
1	4.949	0.0045	4.153	0.0028	3.9705	0.0031	3.7678	0.0030
2	4.9521	0.0076	4.1547	0.0045	3.9682	0.0054	3.7661	0.0047
3	4.955	0.0105	4.1556	0.0054	3.9666	0.0070	3.7649	0.0059
4	4.9582	0.0137	4.1566	0.0064	3.9644	0.0092	3.7632	0.0076
5	4.9631	0.0186	4.1578	0.0076	3.9625	0.0111	3.7614	0.0094
6	4.9661	0.0216	4.1588	0.0086	3.9617	0.0119	3.7599	0.0109
7	4.9677	0.0232	4.159	0.0088	3.9607	0.0129	3.7582	0.0126

Plot of chemical shift change vs guest concentration:

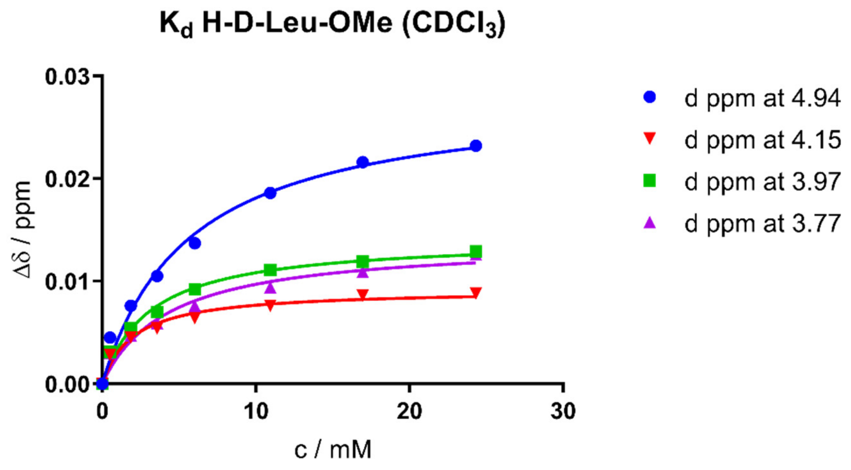

K_d values obtained by fitting the data for selected protons into the single-site specific binding model:

**Kd**	5.79	2.09	3.21	4.42
**R squared**	0.9875	0.9741	0.9882	0.9677

K_d = 3.88 ± 1.37 mM → K_a = 258 ± 91 M^{-1}

H-L-tLeu-OMe x HCl to receptor 1:

^{1}H NMR spectra (top – full, bottom – enhanced view of the receptor signals in the spectra):

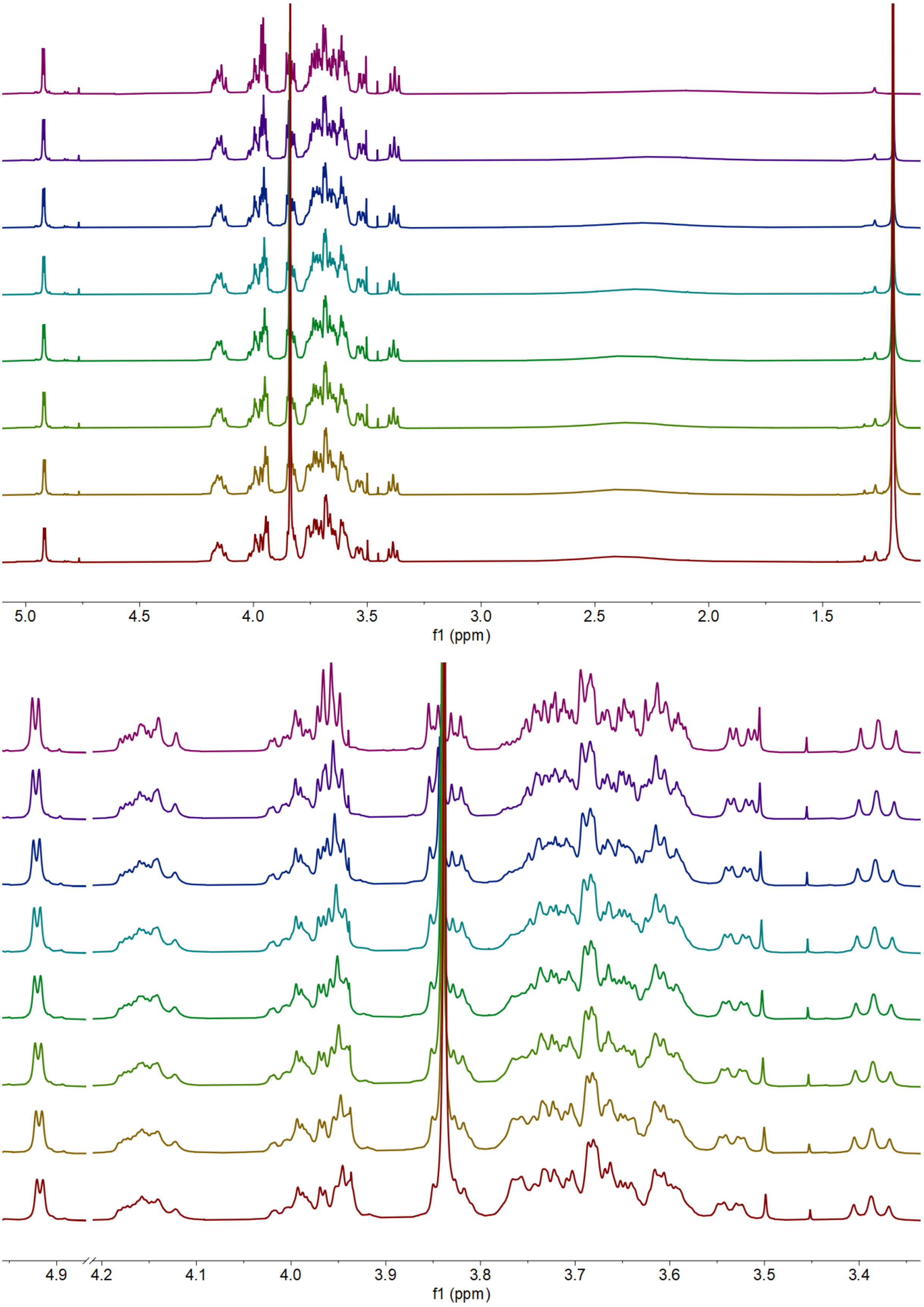

Summary of chemical shift changes:

Titration	4.11	Δppm	3.96	Δppm	3.74	Δppm	3.69	Δppm
0	4.1406	0.0000	3.9621	0.0000	3.7435	0.0000	3.6902	0.0000
1	4.1428	0.0022	3.9593	0.0028	3.7411	0.0024	3.6884	0.0018
2	4.1433	0.0027	3.9572	0.0049	3.7385	0.0050	3.6873	0.0029
3	4.1437	0.0031	3.9563	0.0058	3.7372	0.0063	3.6868	0.0034
4	4.1441	0.0035	3.9553	0.0068	3.7366	0.0069	3.6856	0.0046
5	4.1444	0.0038	3.9544	0.0077	3.7357	0.0078	3.6857	0.0045
6	4.1447	0.0041	3.9537	0.0084	3.735	0.0085	3.6851	0.0051
7	4.145	0.0044	3.953	0.0091	3.7342	0.0093	3.6846	0.0056

Plot of chemical shift change vs guest concentration:

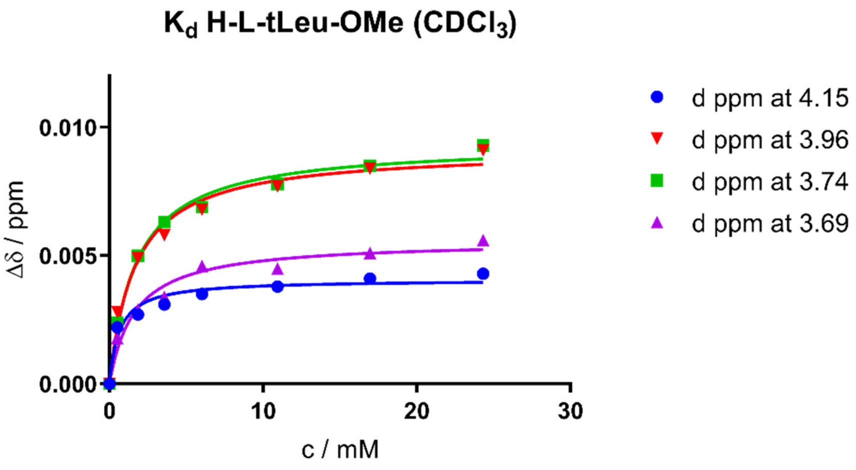

K_d values obtained by fitting the data for selected protons into the single-site specific binding model:

Kd	0.66	1.86	1.92	1.25
R squared	0.9546	0.9904	0.9908	0.9495

K_d = 1.43 ± 0.51 mM → K_a = 699 ± 249 M^{-1}

H-D-tLeu-OMe x HCl to receptor 1:

^{1}H NMR spectra (top – full, bottom – enhanced view of the receptor signals in the spectra):

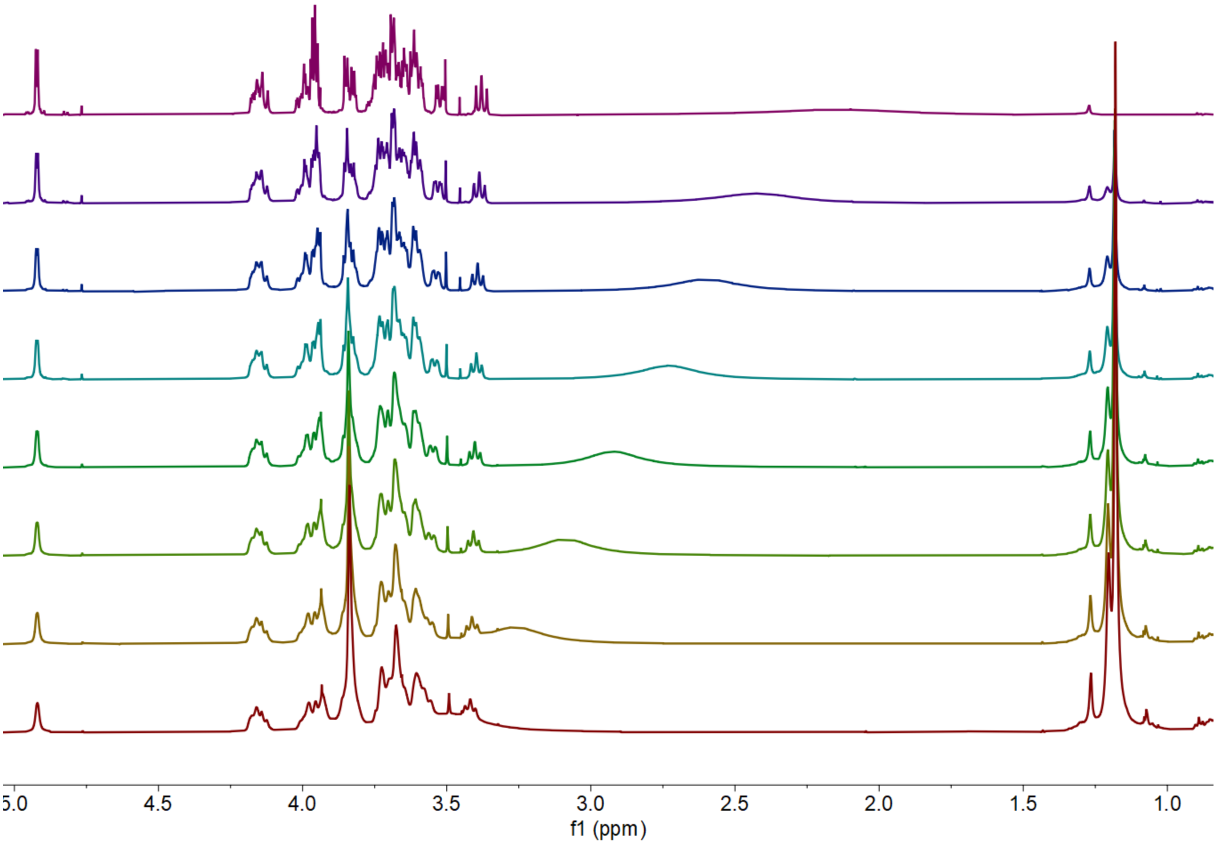

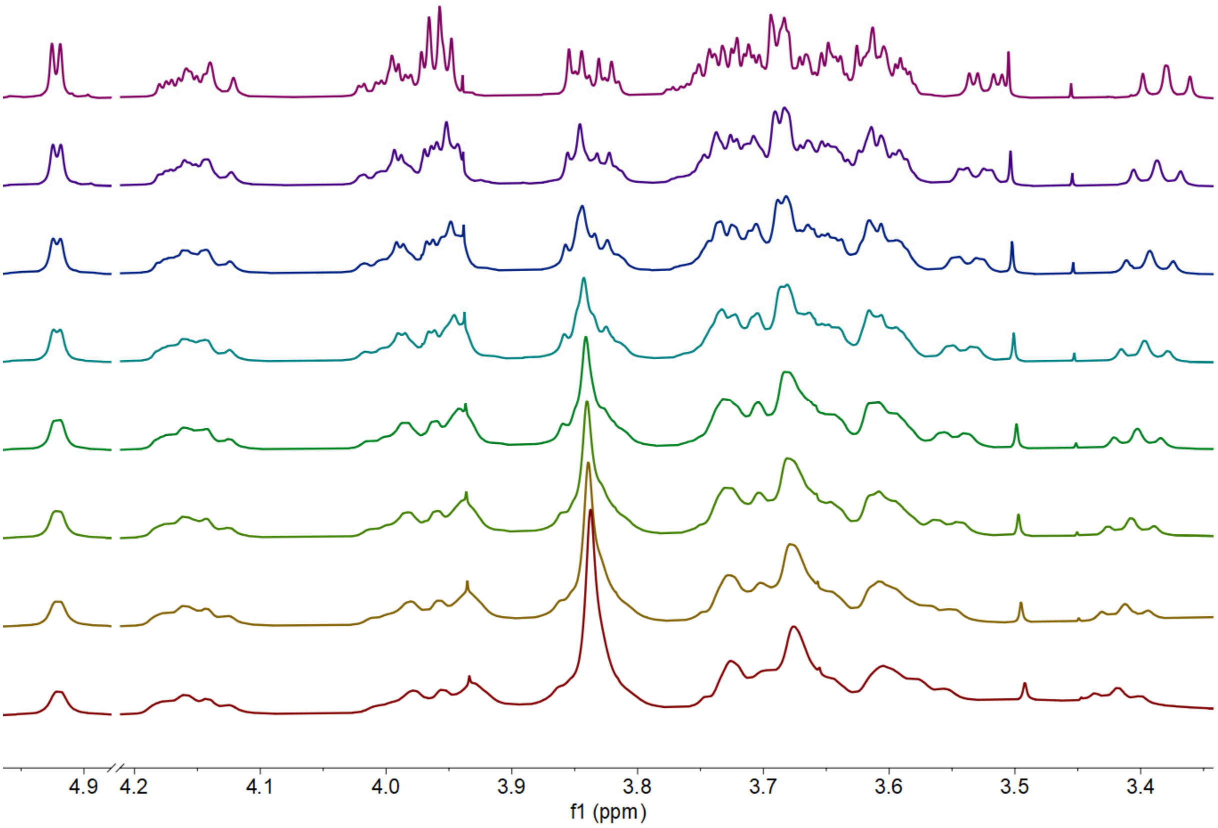

Summary of chemical shift changes:

Titration	4.14	Δppm	3.96	Δppm	3.74	Δppm	3.69	Δppm
0	4.1428	0.0000	3.9766	0.0000	3.7401	0.0000	3.6893	0.0000
1	4.1436	0.0008	3.9747	0.0019	3.7376	0.0025	3.6873	0.0020
2	4.1445	0.0017	3.974	0.0026	3.7355	0.0046	3.6852	0.0041
3	4.1456	0.0028	3.9729	0.0037	3.7332	0.0069	3.6834	0.0059
4	4.1466	0.0038	3.971	0.0056	3.7318	0.0083	3.6815	0.0078
5	4.1475	0.0047	3.9695	0.0071	3.7299	0.0102	3.6795	0.0098
6	4.1483	0.0055	3.9678	0.0088	3.7277	0.0124	3.6775	0.0118
7	4.1495	0.0067	3.9651	0.0115	3.7257	0.0144	3.6754	0.0139

Plot of chemical shift change vs guest concentration:

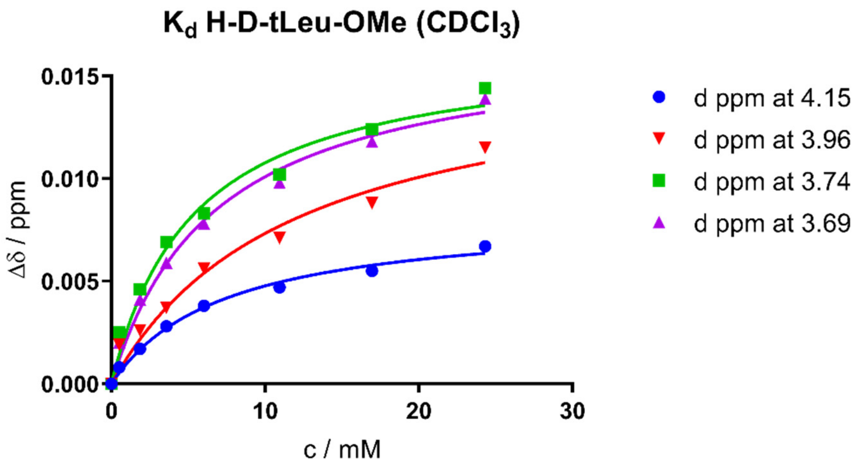

K_d values obtained by fitting the data for selected protons into the single-site specific binding model:

Kd	5.77	11.11	7.79	7.71
R squared	0.9799	0.9689	0.9893	0.9876

K_d = 8.10 ± 1.92 mM → K_a = 123 ± 29 M^{-1}

4.2 Titrations in CD_3CN.

H-L-Ala-OMe x HCl to receptor 1:

^{1}H NMR spectra:

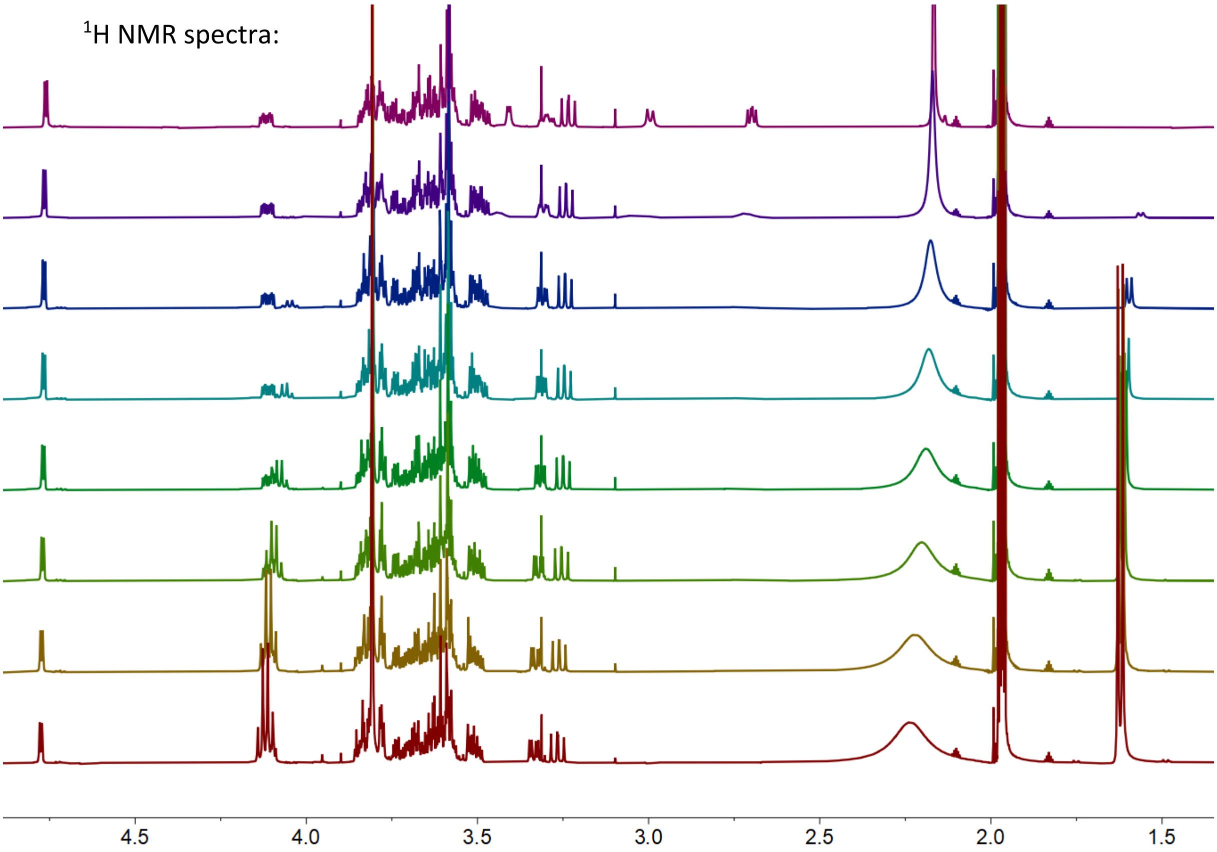

Summary of chemical shift changes:

Titration	4.76	Δppm	4.12	Δppm	3.74	Δppm	3.72	Δppm
0	4.7606	0.0000	4.1003	0.0000	3.7449	0.0000	3.7221	0.0000
1	4.7651	0.0045	4.0968	0.0035	3.7423	0.0026	3.72	0.0021
2	4.7661	0.0055	4.095	0.0053	3.741	0.0039	3.7185	0.0036
3	4.7671	0.0065	4.0937	0.0066	3.7405	0.0044	3.7173	0.0048
4	4.7682	0.0076	4.0923	0.0080	3.7395	0.0054	3.7166	0.0055
5	4.7704	0.0098	4.0912	0.0091	3.7388	0.0061	3.7157	0.0064
6	4.7732	0.0126	4.0898	0.0105	3.7381	0.0068	3.7152	0.0069
7	4.7749	0.0143	4.089	0.0113	3.7377	0.0072	3.7149	0.0072

Plot of chemical shift change vs guest concentration:

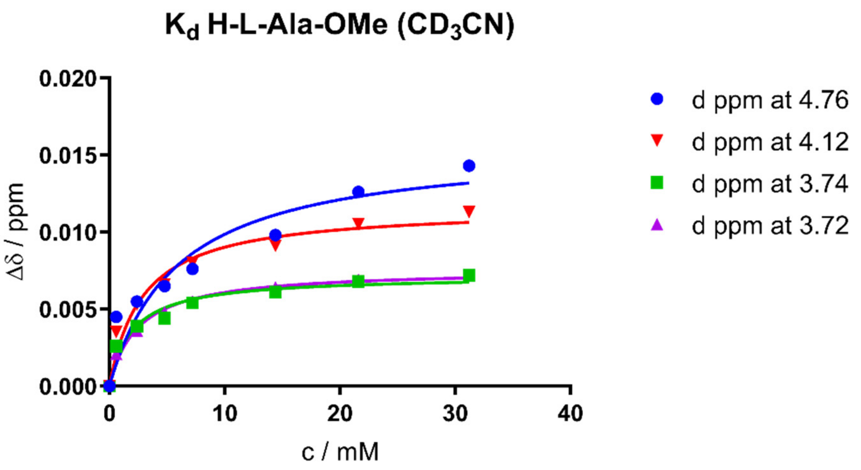

K_d values obtained by fitting the data for selected protons into the single-site specific binding model:

**Kd**	5.05	3.24	2.24	2.81
**R squared**	0.9038	0.9679	0.9639	0.9897

K_d = 3.34 ± 1.05 mM → K_a = 299 ± 94 M^{-1}

H-D-Ala-OMe x HCl to receptor 1:

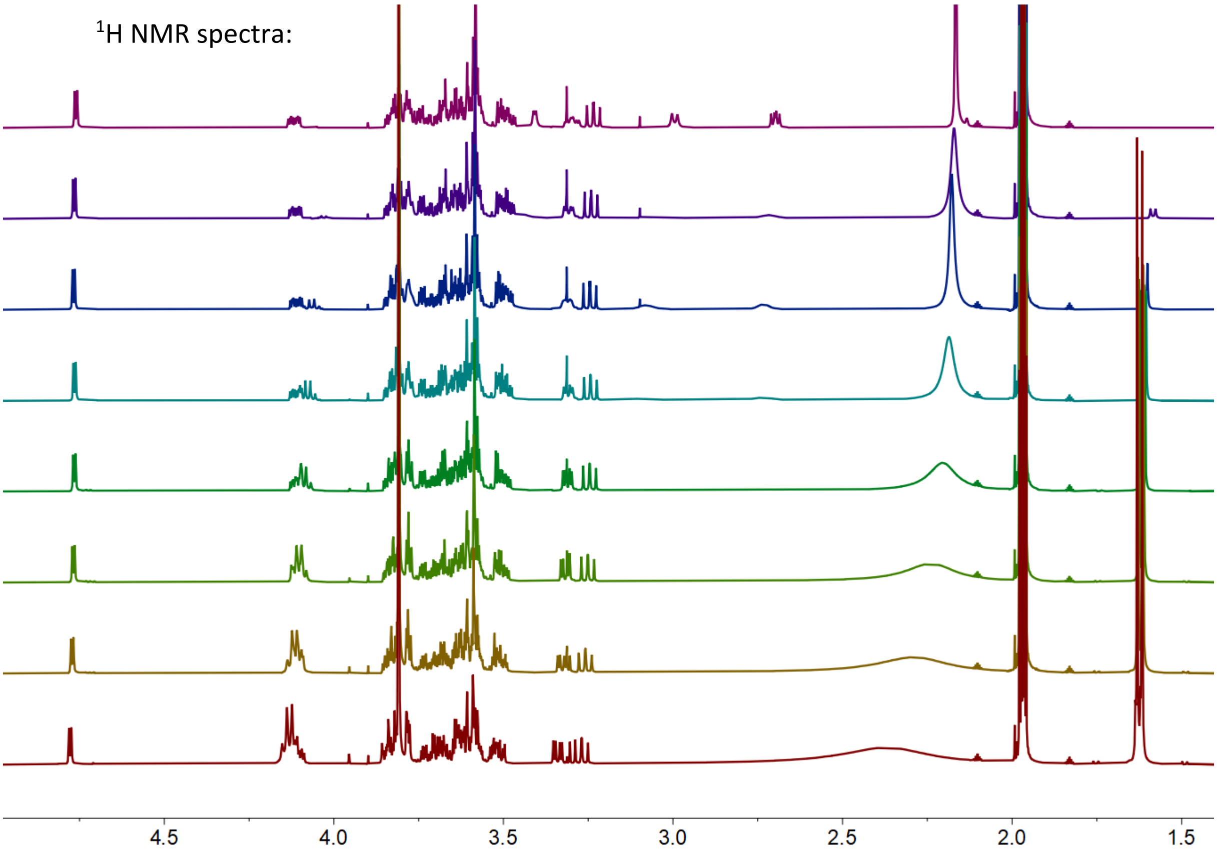

Summary of chemical shift changes:

Titration	4.76	Δppm	4.12	Δppm	3.74	Δppm	3.72	Δppm
0	4.7607	0.0000	4.1004	0.0000	3.7448	0.0000	3.7224	0.0000
1	4.7654	0.0047	4.0961	0.0043	3.7418	0.0030	3.7194	0.0030
2	4.7668	0.0061	4.0938	0.0066	3.7403	0.0045	3.7171	0.0053
3	4.7645	0.0038	4.0941	0.0063	3.74	0.0048	3.7168	0.0056
4	4.7653	0.0046	4.0934	0.0070	3.7389	0.0059	3.7157	0.0067
5	4.7681	0.0074	4.0921	0.0083	3.7369	0.0079	3.7143	0.0081
6	4.7714	0.0107	4.09	0.0104	3.7359	0.0089	3.7128	0.0096
7	4.777	0.0163	4.0877	0.0127	3.7343	0.0105	3.7115	0.0109

Plot of chemical shift change vs guest concentration:

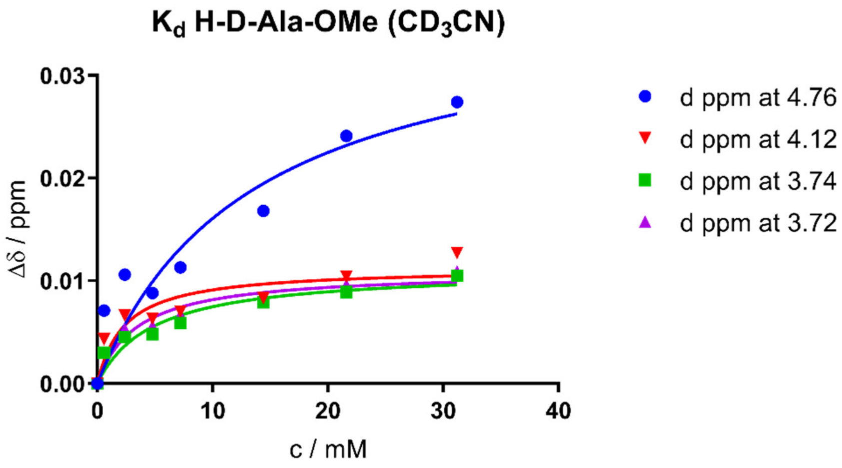

K_d values obtained by fitting the data for selected protons into the single-site specific binding model:

**Kd**	11.65	4.09	7.52	5.6
**R squared**	0.8853	0.8553	0.9321	0.945

K_d = 7.20 ± 2.83 mM → K_a = 139 ± 55 M^{-1}

H-L-Phe-OMe x HCl to receptor 1:

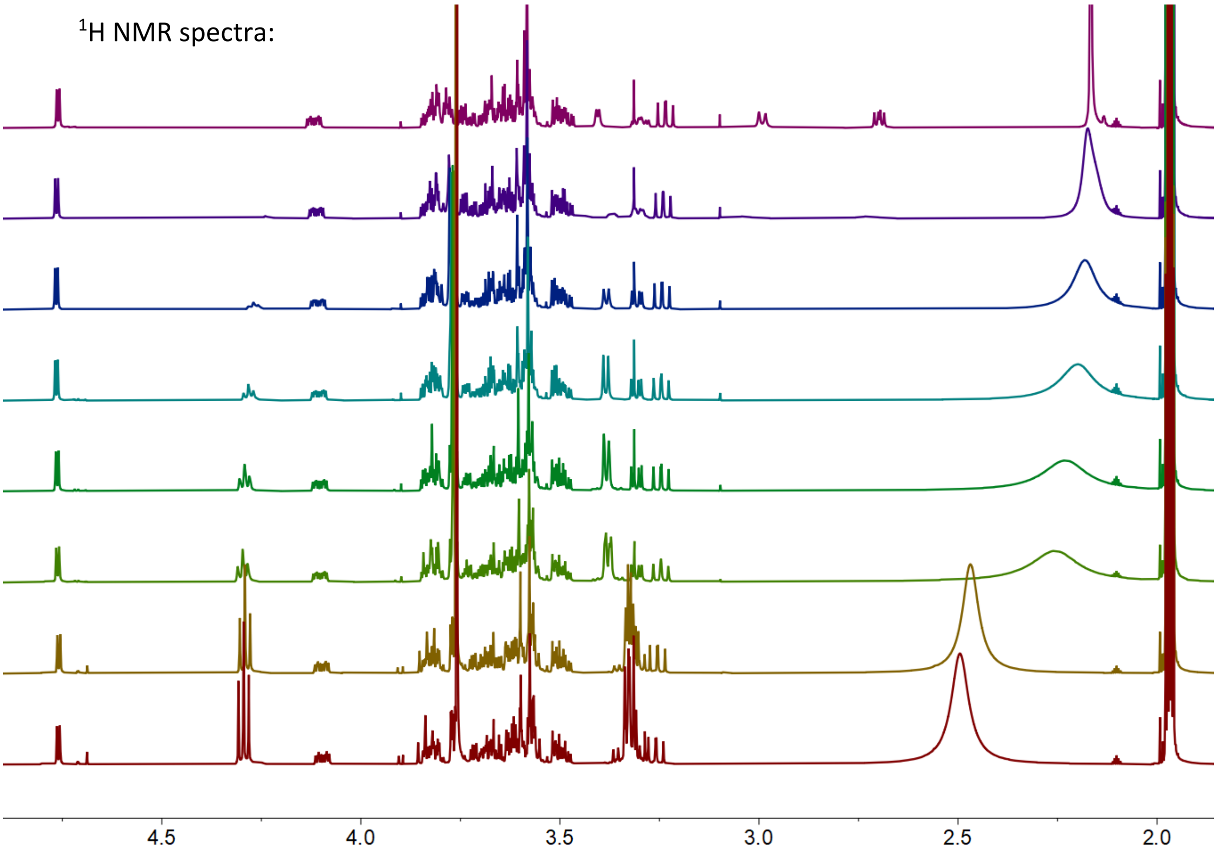

Summary of chemical shift changes:

Titration	4.12	Δppm	3.72	Δppm	3.47	Δppm	3.29	Δppm
0	4.118	0.0000	3.7217	0.0000	3.4924	0.0000	3.2983	0.0000
1	4.1117	0.0063	3.7183	0.0034	3.4951	0.0027	3.3038	0.0055
2	4.1074	0.0106	3.7156	0.0061	3.497	0.0046	3.3072	0.0089
3	4.1055	0.0125	3.713	0.0087	3.4977	0.0053	3.3077	0.0094
4	4.1037	0.0143	3.7105	0.0112	3.4985	0.0061	3.3081	0.0098
5	4.1023	0.0157	3.7083	0.0134	3.4986	0.0062	3.3079	0.0096
6	4.1001	0.0179	3.7072	0.0145	3.499	0.0066	3.3087	0.0104
7	4.0983	0.0197	3.7065	0.0152	3.4994	0.0070	3.3089	0.0106

Plot of chemical shift change vs guest concentration:

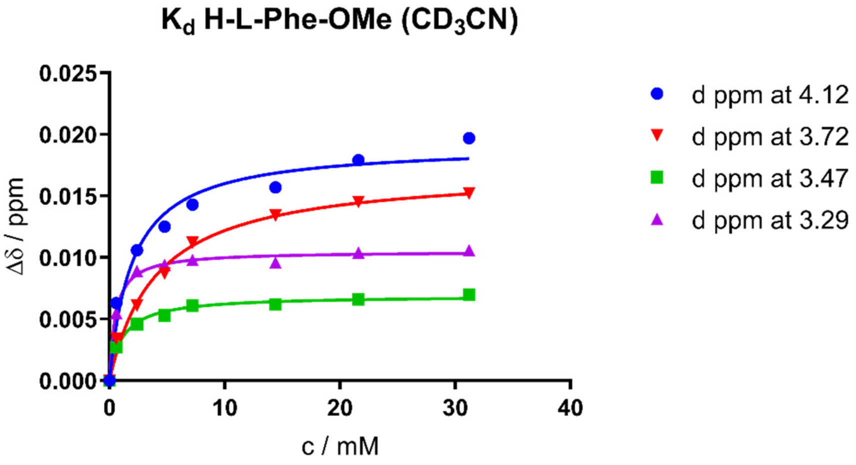

K_d values obtained by fitting the data for selected protons into the single-site specific binding model:

Kd	2.32	3.78	1.31	2.01
R squared	0.9712	0.9931	0.9929	0.9949

K_d = 2.36 ± 0.90 mM → K_a = 424 ± 161 M^{-1}

H-D-Phe-OMe x HCl to receptor 1:

^{1}H NMR spectra:

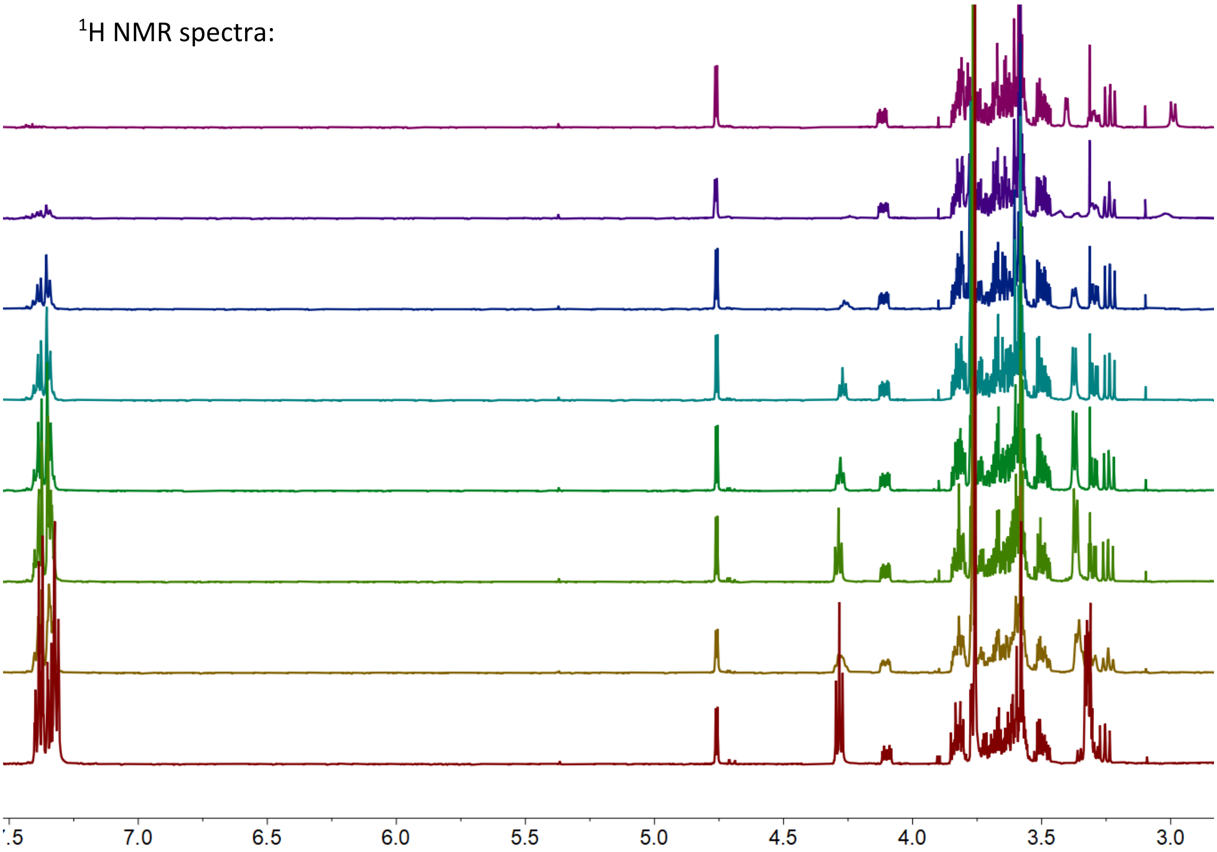

Summary of chemical shift changes:

Titration	4.12	Δppm	3.72	Δppm	3.47	Δppm	3.29	Δppm
0	4.1181	0.0000	3.7222	0.0000	3.4756	0.0000	3.2924	0.0000
1	4.1137	0.0044	3.7203	0.0019	3.4762	0.0006	3.2950	0.0026
2	4.1116	0.0065	3.7186	0.0036	3.4769	0.0013	3.2953	0.0029
3	4.1095	0.0086	3.7159	0.0063	3.4771	0.0015	3.2966	0.0042
4	4.1076	0.0105	3.7136	0.0086	3.4772	0.0016	3.2989	0.0065
5	4.1056	0.0125	3.7107	0.0115	3.4776	0.0020	3.2992	0.0068
6	4.105	0.0131	3.7101	0.0121	3.4778	0.0022	3.3015	0.0091
7	4.1004	0.0177	3.7093	0.0129	3.478	0.0024	3.3028	0.0104

Plot of chemical shift change vs guest concentration:

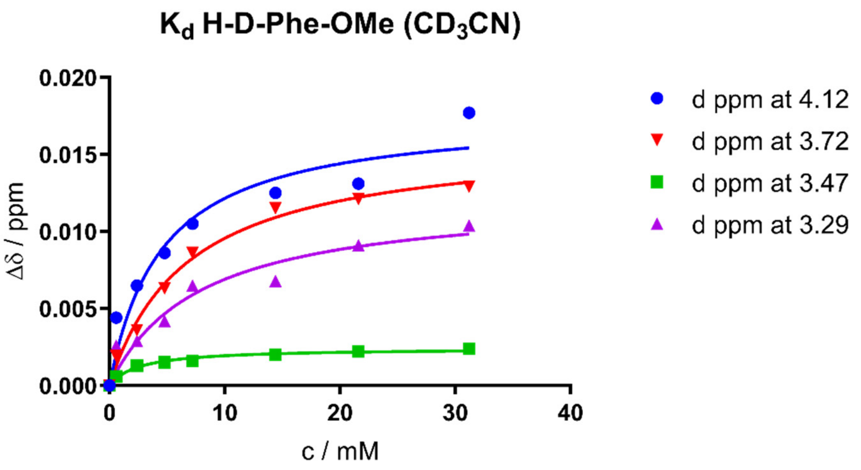

K_d values obtained by fitting the data for selected protons into the single-site specific binding model:

**Kd**	5.24	7.18	7.7	8.18
**R squared**	0.9363	0.9926	0.9785	0.9439

K_d = 7.08 ± 1.1 mM → K_a = 141 ± 22 M^{-1}

H-L-Pro-OMe x HCl to receptor 1:

^{1}H NMR spectra:

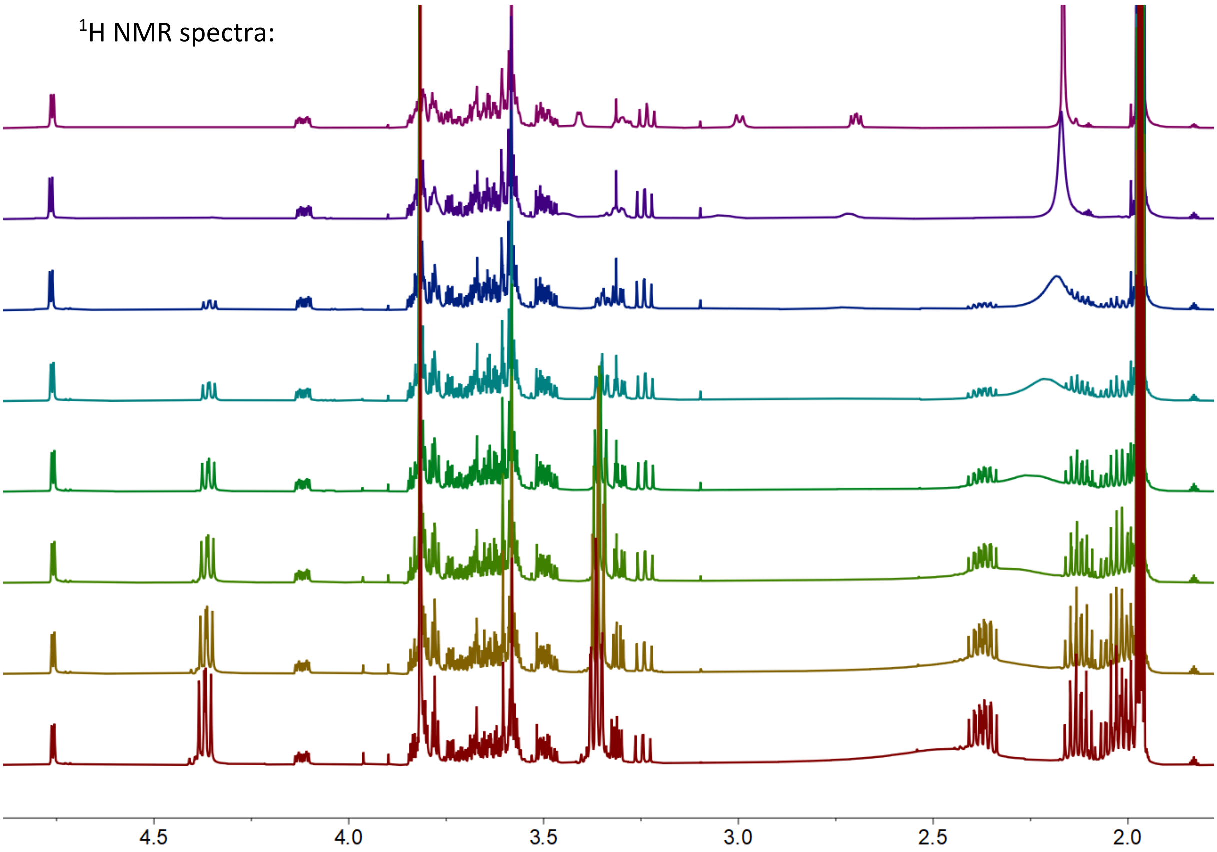

Summary of chemical shift changes:

Titration	4.76	Δppm	4.12	Δppm	3.74	Δppm	3.72	Δppm
0	4.7647	0.0000	4.1137	0.0000	3.7453	0.0000	3.7223	0.0000
1	4.764	0.0007	4.1147	0.0010	3.7429	0.0024	3.7195	0.0028
2	4.761	0.0037	4.1148	0.0011	3.7414	0.0039	3.7191	0.0032
3	4.7594	0.0053	4.1172	0.0035	3.7417	0.0036	3.7194	0.0029
4	4.7589	0.0058	4.1185	0.0048	3.7412	0.0041	3.7188	0.0035
5	4.7585	0.0062	4.1192	0.0055	3.7408	0.0045	3.7183	0.0040
6	4.7582	0.0065	4.1195	0.0058	3.74	0.0053	3.7174	0.0049
7	4.7575	0.0072	4.1203	0.0066	3.737	0.0083	3.7158	0.0065

Plot of chemical shift change vs guest concentration:

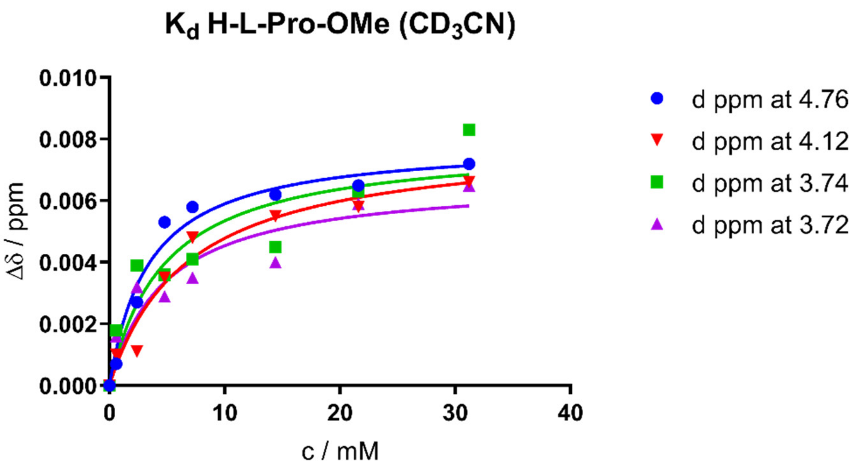

K_d values obtained by fitting the data for selected protons into the single-site specific binding model:

Kd	7.21	7.13	9.67	4.99
R squared	0.9685	0.9602	0.8456	0.8831

K_d = 7.25 ± 1.7 mM → K_a = 138 ± 32 M^{-1}

H-D-Pro-OMe x HCl to receptor 1:

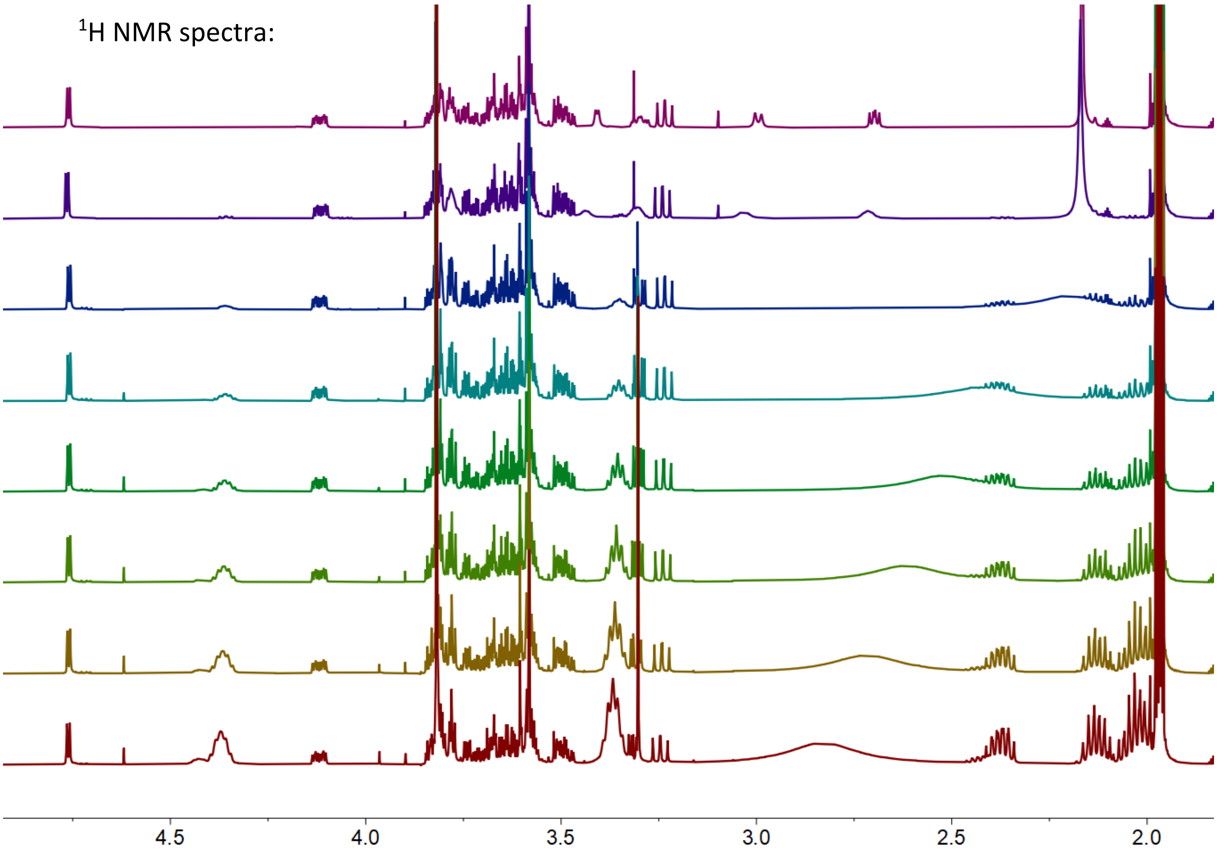

Summary of chemical shift changes:

Titration	4.76	Δppm	4.12	Δppm	3.74	Δppm	3.72	Δppm
0	4.758	0.0000	4.1174	0.0000	3.7452	0.0000	3.7223	0.0000
1	4.7584	0.0004	4.1176	0.0002	3.7434	0.0018	3.721	0.0013
2	4.7589	0.0009	4.1182	0.0008	3.7439	0.0013	3.7212	0.0011
3	4.7592	0.0012	4.1186	0.0012	3.7433	0.0019	3.7207	0.0016
4	4.7594	0.0014	4.1188	0.0014	3.7429	0.0023	3.7203	0.0020
5	4.7599	0.0019	4.1189	0.0015	3.742	0.0032	3.7193	0.0030
6	4.7604	0.0024	4.1191	0.0017	3.7411	0.0041	3.7185	0.0038
7	4.7613	0.0033	4.1195	0.0021	3.74	0.0052	3.7172	0.0051

Plot of chemical shift change vs guest concentration:

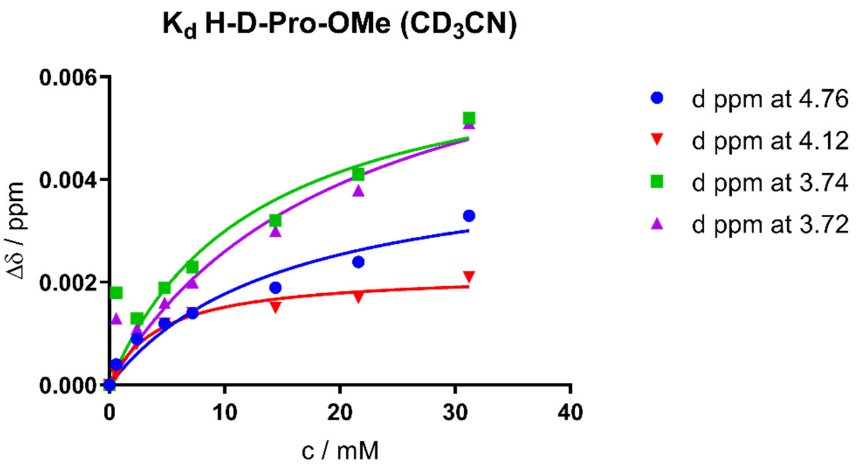

K_d values obtained by fitting the data for selected protons into the single-site specific binding model:

**Kd**	15.01	6.67	12.98	20.75
**R squared**	0.9529	0.9743	0.8603	0.9208

K_d = 13.85 ± 5.0 mM → K_a = 72 ± 26 M^{-1}

H-L-Thr-OMe x HCl to receptor 1:

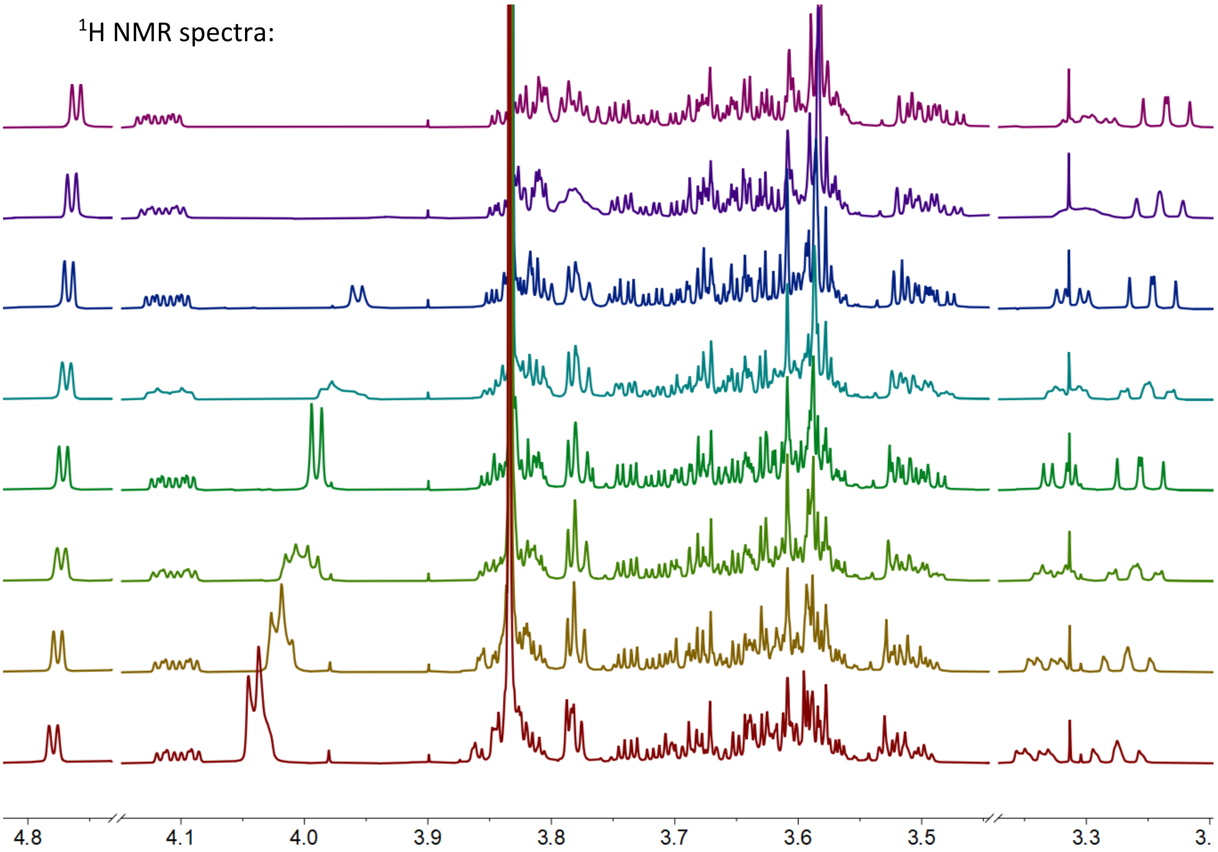

Summary of chemical shift changes:

Titration	4.76	Δppm	4.12	Δppm	3.74	Δppm	3.72	Δppm
0	4.7609	0.0000	4.1181	0.0000	3.7447	0.0000	3.7219	0.0000
1	4.7646	0.0037	4.115	0.0031	3.7425	0.0022	3.72	0.0019
2	4.7671	0.0062	4.1111	0.0070	3.7408	0.0039	3.7177	0.0042
3	4.769	0.0081	4.1091	0.0090	3.7398	0.0049	3.7168	0.0051
4	4.7711	0.0102	4.1065	0.0116	3.7391	0.0056	3.7162	0.0057
5	4.7732	0.0123	4.1055	0.0126	3.7384	0.0063	3.7155	0.0064
6	4.7751	0.0142	4.1037	0.0144	3.738	0.0067	3.715	0.0069
7	4.7759	0.0150	4.103	0.0151	3.7378	0.0069	3.7148	0.0071

Plot of chemical shift change vs guest concentration:

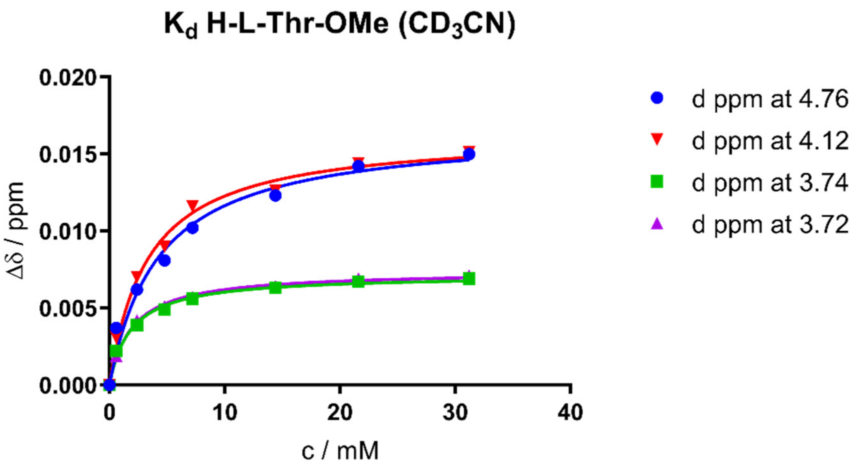

K_d values obtained by fitting the data for selected protons into the single-site specific binding model:

**Kd**	4.47	3.54	3	2.07
**R squared**	0.983	0.9949	0.9935	0.998

K_d = 3.27 ± 0.87 mM → K_a = 306 ± 81 M^{-1}

H-D-Thr-OMe x HCl to receptor 1:

^{1}H NMR spectra:

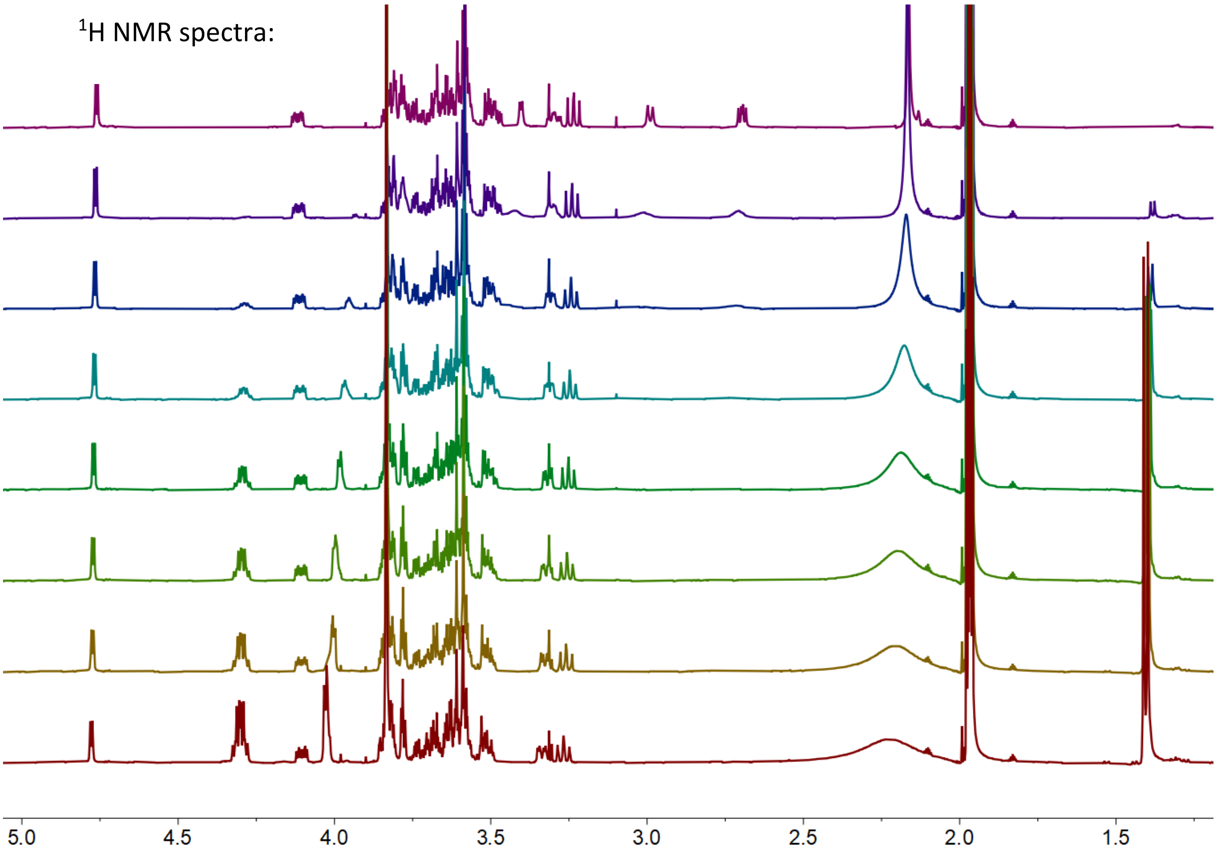

Summary of chemical shift changes:

Titration	4.76	Δppm	4.12	Δppm	3.74	Δppm	3.72	Δppm
0	4.7608	0.0000	4.118	0.0000	3.7448	0.0000	3.7222	0.0000
1	4.7646	0.0038	4.1148	0.0032	3.7427	0.0021	3.72	0.0022
2	4.7661	0.0053	4.1127	0.0053	3.7412	0.0036	3.7183	0.0039
3	4.768	0.0072	4.1102	0.0078	3.7399	0.0049	3.7169	0.0053
4	4.7701	0.0093	4.1083	0.0097	3.7387	0.0061	3.7158	0.0064
5	4.7723	0.0115	4.1069	0.0111	3.7381	0.0067	3.715	0.0072
6	4.774	0.0132	4.106	0.0120	3.7374	0.0074	3.7147	0.0075
7	4.7769	0.0161	4.1043	0.0137	3.737	0.0078	3.714	0.0082

Plot of chemical shift change vs guest concentration:

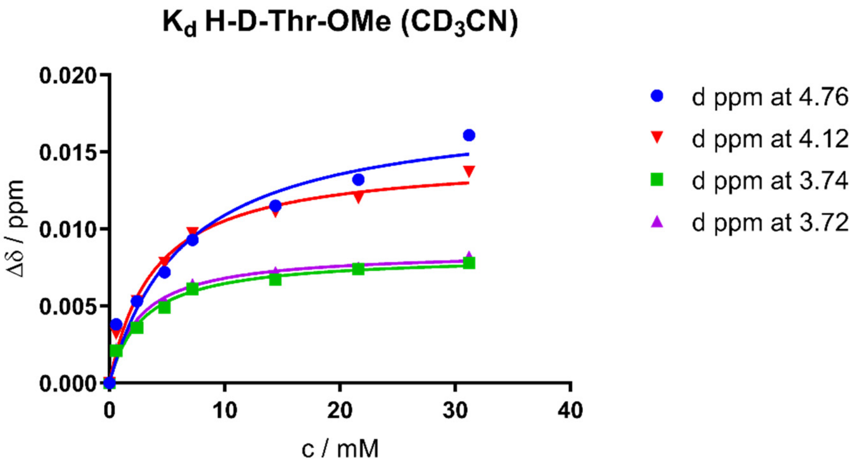

K_d values obtained by fitting the data for selected protons into the single-site specific binding model:

**Kd**	6.72	4.07	4.04	5.1
**R squared**	0.96	0.9847	0.9893	0.9914

K_d = 4.98 ± 1.09 mM → K_a = 201 ± 44 M^{-1}

H-L-Val-OMe x HCl to receptor 1:

^{1}H NMR spectra:

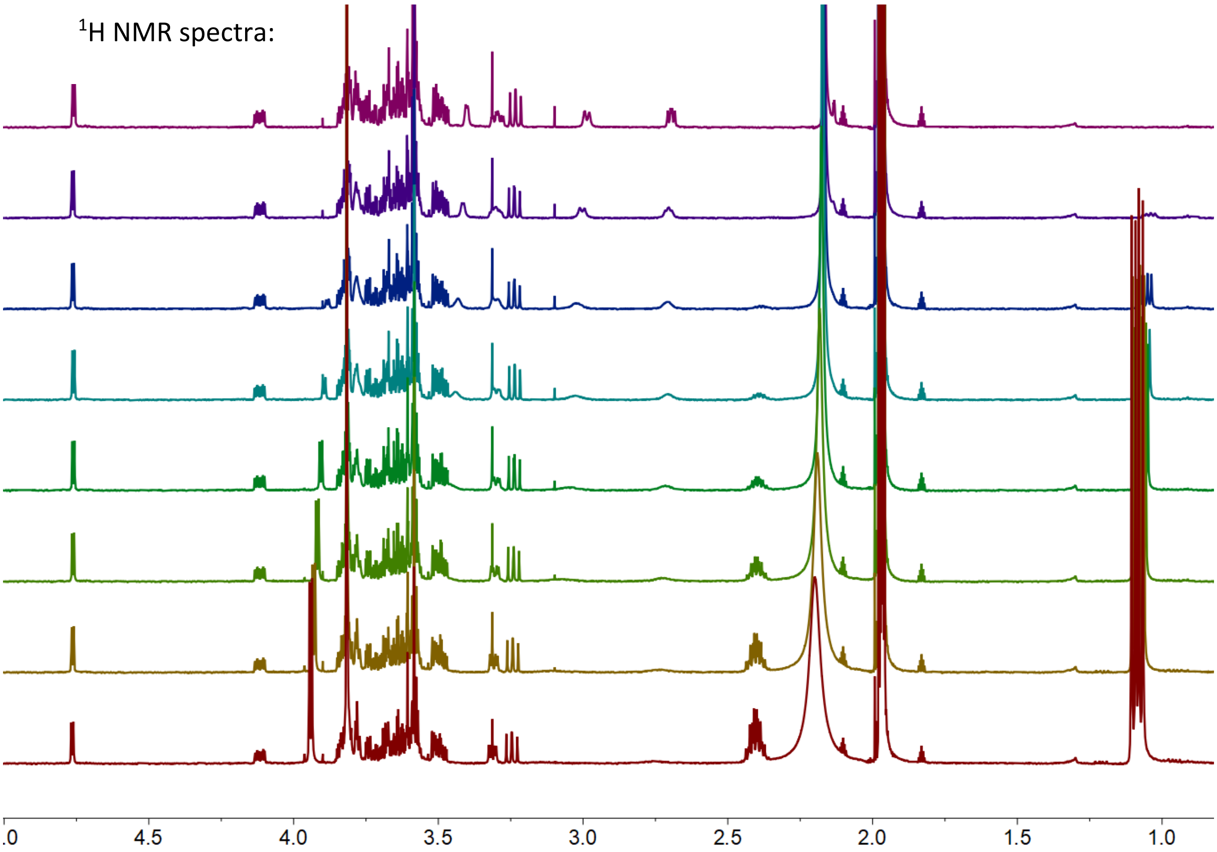

Summary of chemical shift changes:

Titration	4.12	Δppm	3.74	Δppm	3.72	Δppm	3.48	Δppm
0	4.1181	0.0000	3.7455	0.0000	3.7222	0.0000	3.4867	0.0000
1	4.116	0.0021	3.7441	0.0014	3.7211	0.0011	3.4882	0.0015
2	4.1159	0.0022	3.7438	0.0017	3.721	0.0012	3.4888	0.0021
3	4.1143	0.0038	3.7437	0.0018	3.7208	0.0014	3.4892	0.0025
4	4.1141	0.0040	3.7432	0.0023	3.7205	0.0017	3.4898	0.0031
5	4.1136	0.0045	3.7428	0.0027	3.72	0.0022	3.4904	0.0037
6	4.1134	0.0047	3.7424	0.0031	3.7197	0.0025	3.4907	0.0040
7	4.1132	0.0049	3.7423	0.0032	3.7195	0.0027	3.4909	0.0042

Plot of chemical shift change vs guest concentration:

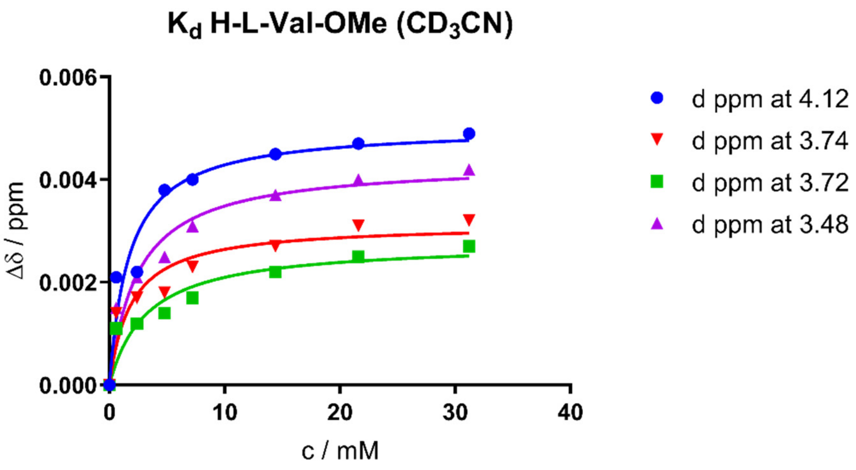

K_d values obtained by fitting the data for selected protons into the single-site specific binding model:

Kd	1.82	2.06	3.38	2.61
**R squared**	0.9385	0.9097	0.8954	0.962

K_d = 2.47 ± 0.60 mM → K_a = 405 ± 98 M^{-1}

H-D-Val-OMe x HCl to receptor 1:

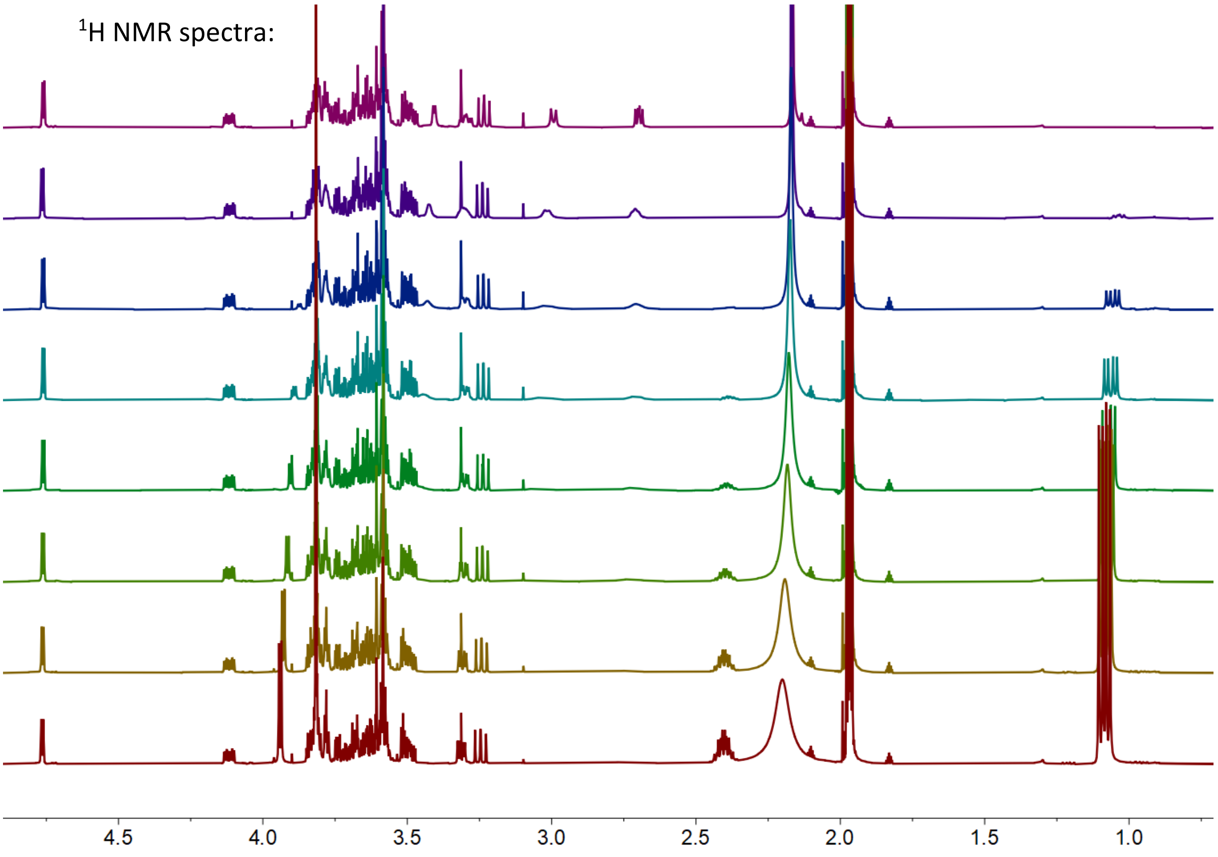

Summary of chemical shift changes:

Titration	4.12	Δppm	3.74	Δppm	3.72	Δppm	3.48	Δppm
0	4.1176	0.0000	3.7449	0.0000	3.7224	0.0000	3.4849	0.0000
1	4.1169	0.0007	3.7442	0.0007	3.7215	0.0009	3.4861	0.0012
2	4.1171	0.0005	3.744	0.0009	3.7212	0.0012	3.4863	0.0014
3	4.117	0.0006	3.7441	0.0008	3.7209	0.0015	3.4867	0.0018
4	4.1168	0.0008	3.7435	0.0014	3.7203	0.0021	3.4876	0.0027
5	4.1166	0.0010	3.743	0.0019	3.7198	0.0026	3.4882	0.0033
6	4.1165	0.0011	3.7423	0.0026	3.7195	0.0029	3.4888	0.0039
7	4.1163	0.0013	3.742	0.0029	3.7193	0.0031	3.4892	0.0043

Plot of chemical shift change vs guest concentration:

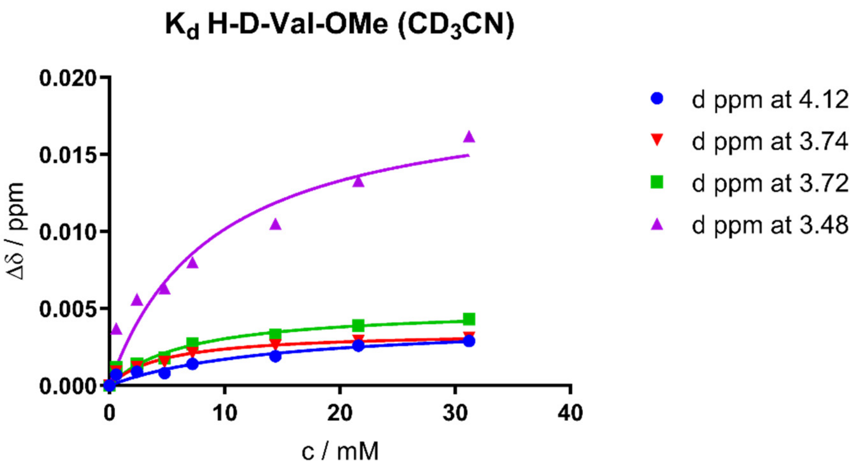

K_d values obtained by fitting the data for selected protons into the single-site specific binding model:

Kd	6.02	7.97	9.03	11.25
R squared	0.9377	0.9653	0.9559	0.9372

$$\mathbf{K_d = 8.57 \pm 1.89\ mM \rightarrow K_a = 117 \pm 26\ M^{-1}}$$

4.3 Titrations in DMSO-d_6.

H-L-Ala-OMe x HCl to receptor 1:

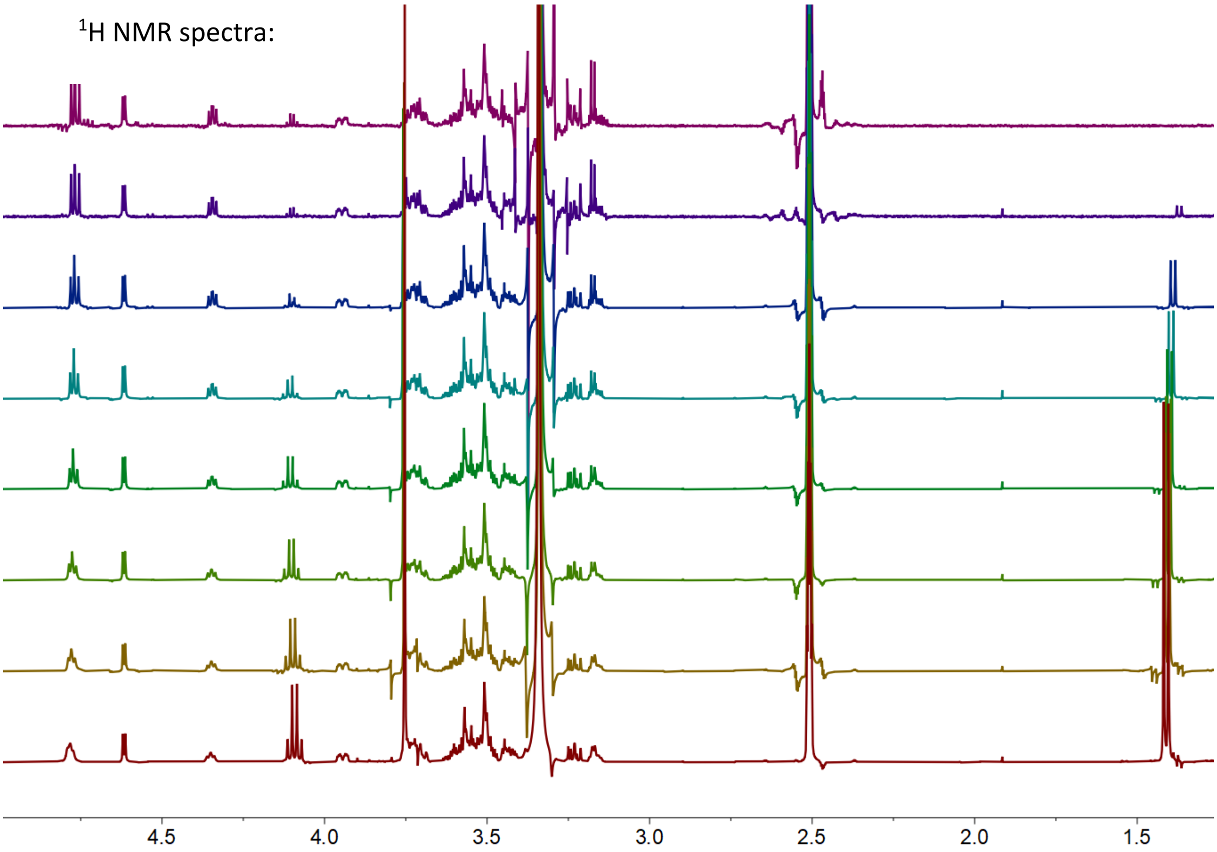

Summary of chemical shift changes:

Titration	4.76	Δppm	4.34	Δppm	3.94	Δppm	3.71	Δppm
0	4.7676	0.0000	4.3444	0.0000	3.9467	0.0000	3.7076	0.0000
1	4.7684	0.0008	4.345	0.0006	3.9463	0.0004	3.7071	0.0005
2	4.7698	0.0022	4.3456	0.0012	3.9457	0.0010	3.7069	0.0007
3	4.7715	0.0039	4.346	0.0016	3.9456	0.0011	3.7066	0.0010
4	4.7732	0.0056	4.3465	0.0021	3.9454	0.0013	3.7063	0.0013
5	4.7757	0.0081	4.3475	0.0031	3.945	0.0017	3.7061	0.0015
6	4.7771	0.0095	4.3485	0.0041	3.9445	0.0022	3.7058	0.0018
7	4.7779	0.0103	4.3493	0.0049	3.9442	0.0025	3.7053	0.0023

Plot of chemical shift change vs guest concentration:

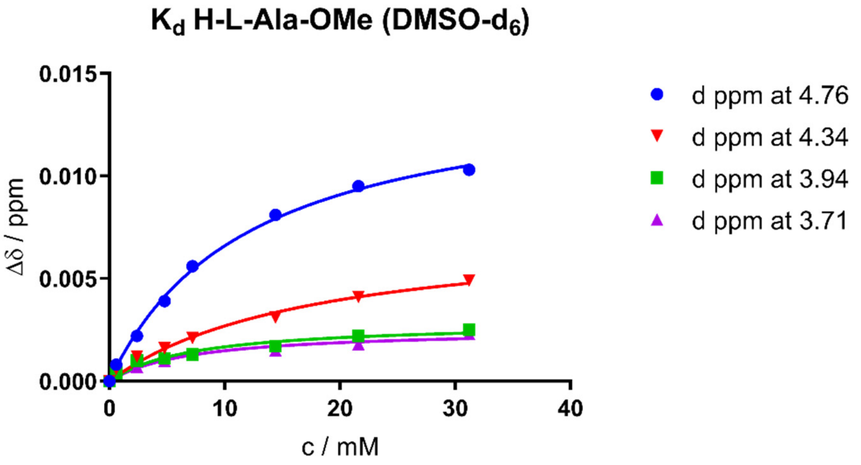

K_d values obtained by fitting the data for selected protons into the single-site specific binding model:

**Kd**	12.8	13.21	7.53	7.45
**R squared**	0.9983	0.9848	0.96	0.9493

K_d = 10.25 ± 2.76 mM → K_a = 98 ± 26 M^{-1}

H-D-Ala-OMe x HCl to receptor 1:

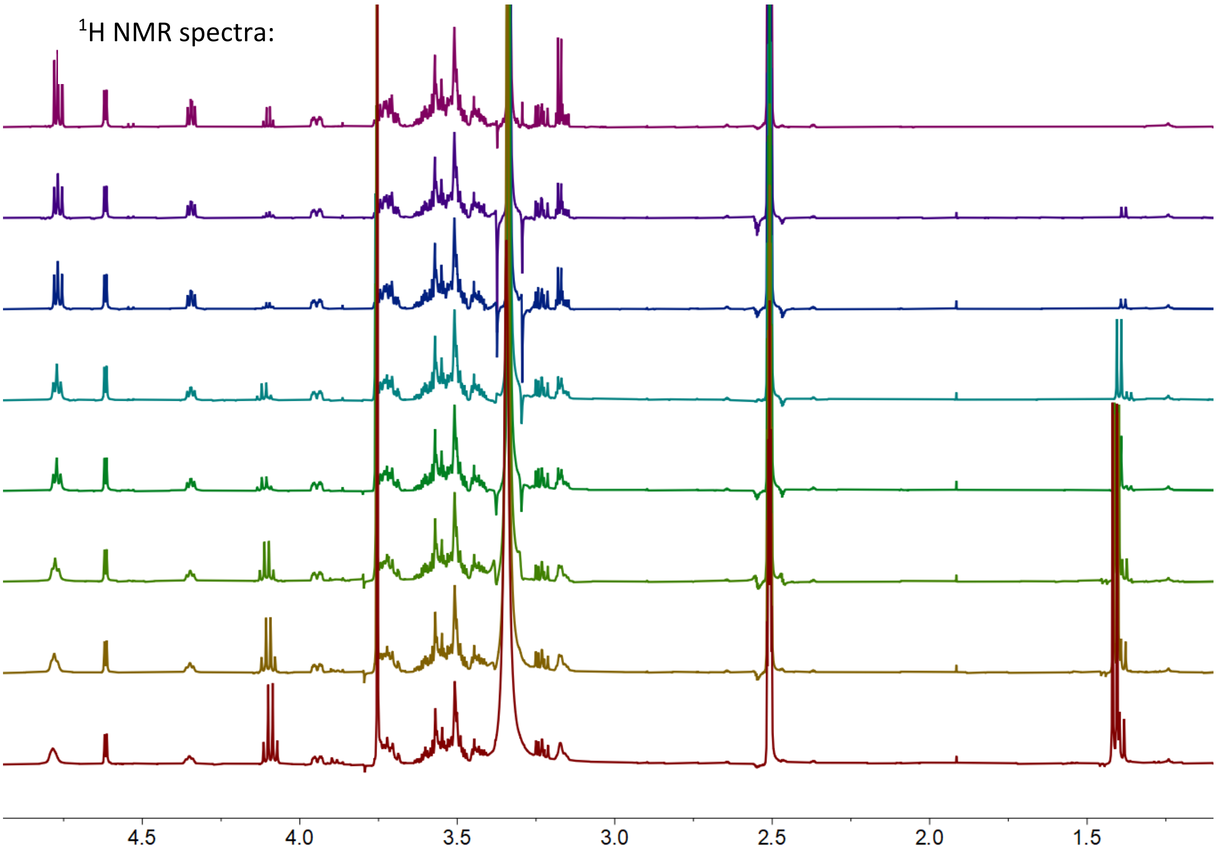

Summary of chemical shift changes:

Titration	4.76	Δppm	4.34	Δppm	3.94	Δppm	3.57	Δppm
0	4.7678	0.0000	4.3444	0.0000	3.9467	0.0000	3.5712	0.0000
1	4.7685	0.0007	4.3448	0.0004	3.9464	0.0003	3.5710	0.0002
2	4.7702	0.0024	4.3453	0.0009	3.946	0.0007	3.5707	0.0005
3	4.7725	0.0047	4.3458	0.0014	3.9458	0.0009	3.5705	0.0007
4	4.7733	0.0055	4.347	0.0026	3.9455	0.0012	3.5703	0.0009
5	4.7763	0.0085	4.3478	0.0034	3.945	0.0017	3.5701	0.0011
6	4.7792	0.0114	4.3486	0.0042	3.9446	0.0021	3.5697	0.0015
7	4.7805	0.0127	4.3501	0.0057	3.9442	0.0025	3.5692	0.0020

Plot of chemical shift change vs guest concentration:

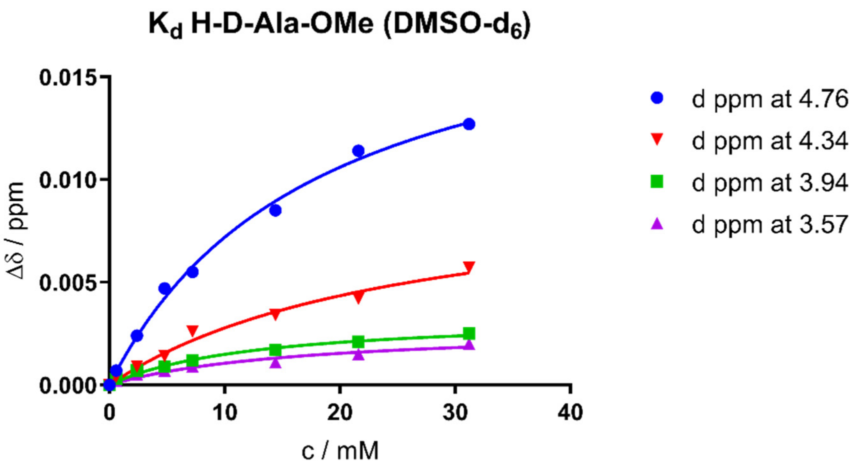

K_d values obtained by fitting the data for selected protons into the single-site specific binding model:

**Kd**	19.17	27.76	13.33	17.64
**R squared**	0.9925	0.9874	0.9878	0.9617

K_d = 19.48 ± 5.24 mM → K_a = 51 ± 14 M^{-1}

H-L-Phe-OMe x HCl to receptor 1:

^{1}H NMR spectra:

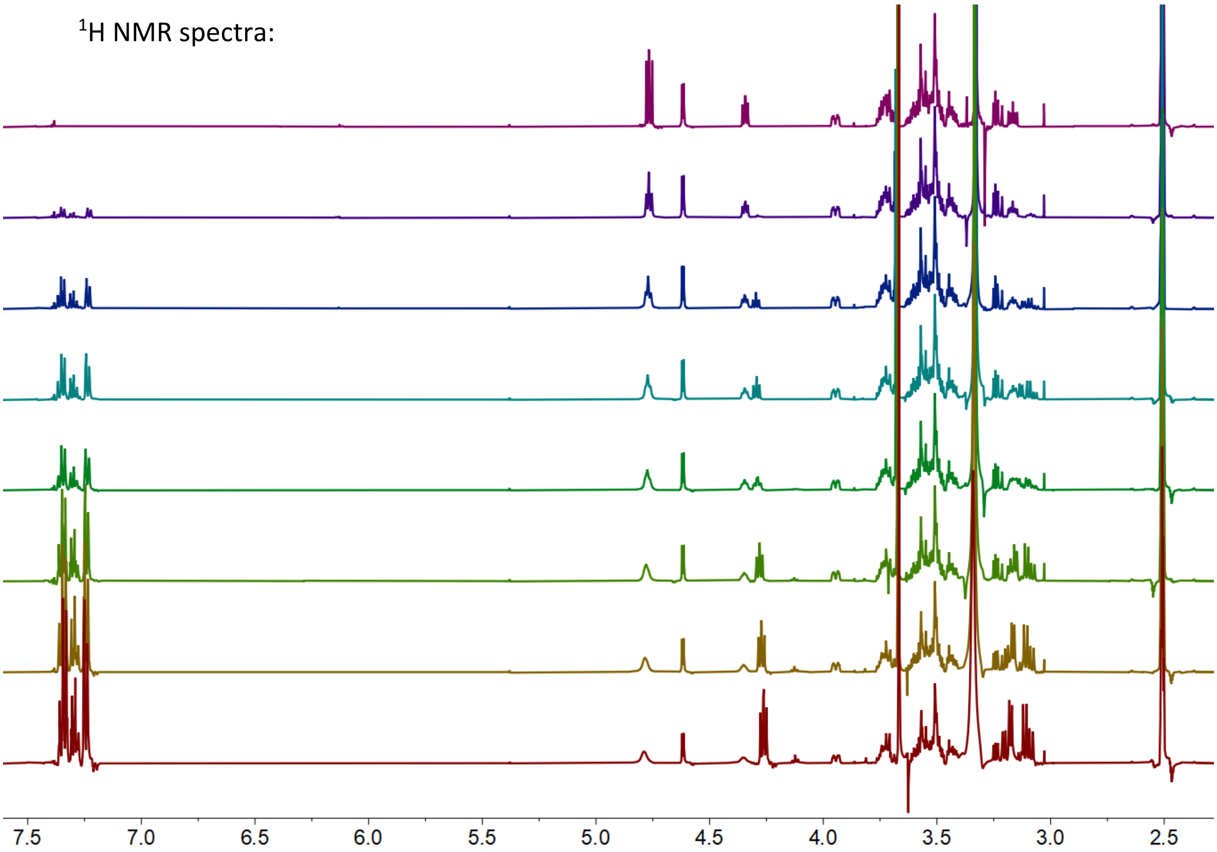

Summary of chemical shift changes:

Titration	4.34	Δppm	3.94	Δppm	3.71	Δppm	3.57	Δppm
0	4.3428	0.0000	3.9467	0.0000	3.7076	0.0000	3.5714	0.0000
1	4.3438	0.0010	3.9461	0.0006	3.707	0.0006	3.5710	0.0004
2	4.3453	0.0025	3.9455	0.0012	3.7066	0.0010	3.5707	0.0007
3	4.3457	0.0029	3.9454	0.0013	3.7065	0.0011	3.5706	0.0008
4	4.3462	0.0034	3.9452	0.0015	3.7063	0.0013	3.5705	0.0009
5	4.3471	0.0043	3.9449	0.0018	3.7059	0.0017	3.5700	0.0014
6	4.3485	0.0057	3.9446	0.0021	3.7055	0.0021	3.5699	0.0015
7	4.3489	0.0061	3.9442	0.0025	3.7052	0.0024	3.5695	0.0019

Plot of chemical shift change vs guest concentration:

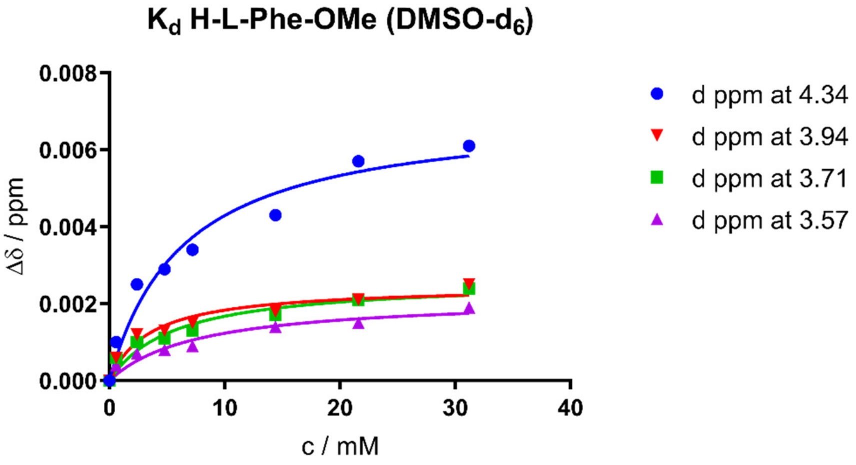

K_d values obtained by fitting the data for selected protons into the single-site specific binding model:

**Kd**	6.63	3.66	5.83	7.91
**R squared**	0.9666	0.9483	0.9396	0.9448

K_d = 6.00 ± 1.54 mM → K_a = 167 ± 43 M^{-1}

H-D-Phe-OMe x HCl to receptor 1:

^{1}H NMR spectra:

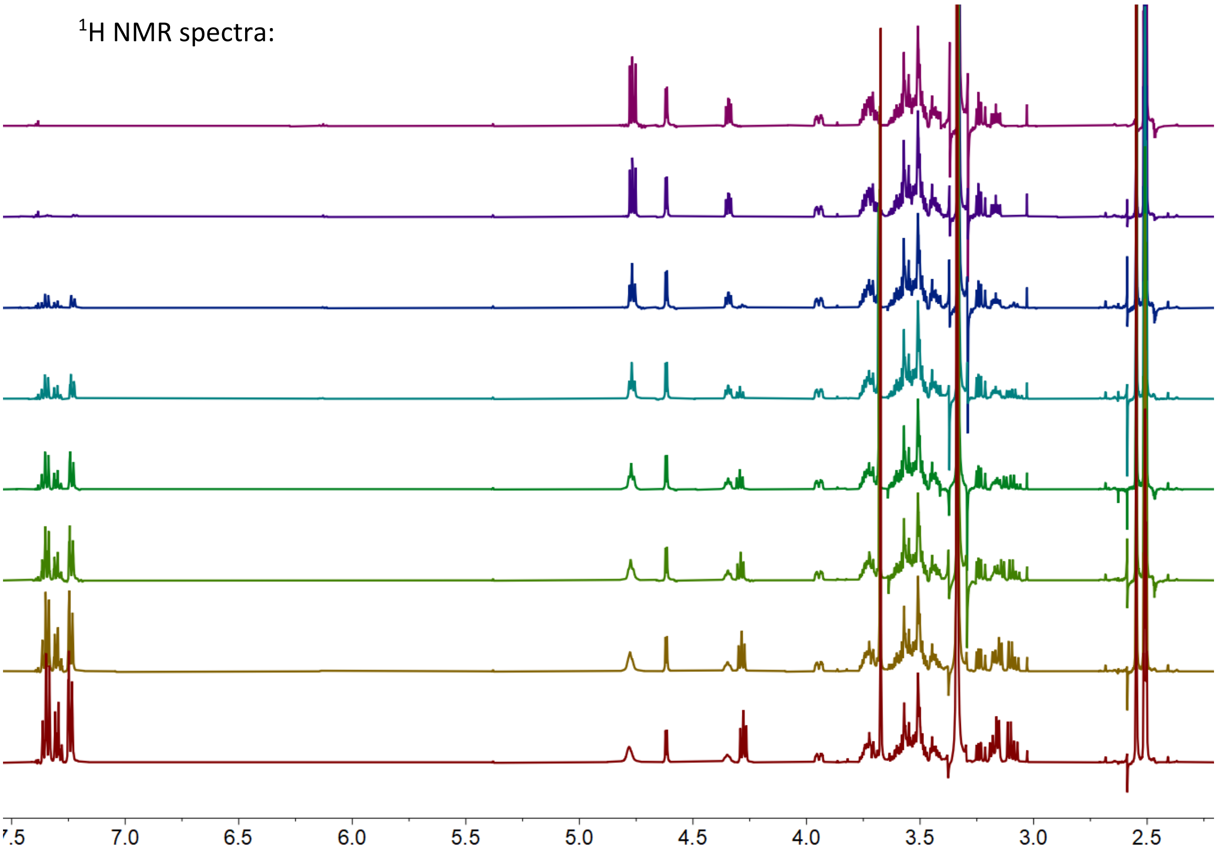

Summary of chemical shift changes:

Titration	4.34	Δppm	3.94	Δppm	3.71	Δppm	3.57	Δppm
0	4.3429	0.0000	3.9464	0.0000	3.7078	0.0000	3.5713	0.0000
1	4.3434	0.0005	3.9461	0.0003	3.7075	0.0003	3.5711	0.0002
2	4.344	0.0011	3.9459	0.0005	3.7072	0.0006	3.5709	0.0004
3	4.3447	0.0018	3.9458	0.0006	3.7069	0.0009	3.5708	0.0005
4	4.3455	0.0026	3.9454	0.0010	3.7067	0.0011	3.5706	0.0007
5	4.3463	0.0034	3.9453	0.0011	3.7063	0.0015	3.5703	0.0010
6	4.3471	0.0042	3.945	0.0014	3.706	0.0018	3.5700	0.0013
7	4.3487	0.0058	3.9447	0.0017	3.7056	0.0022	3.5696	0.0017

Plot of chemical shift change vs guest concentration:

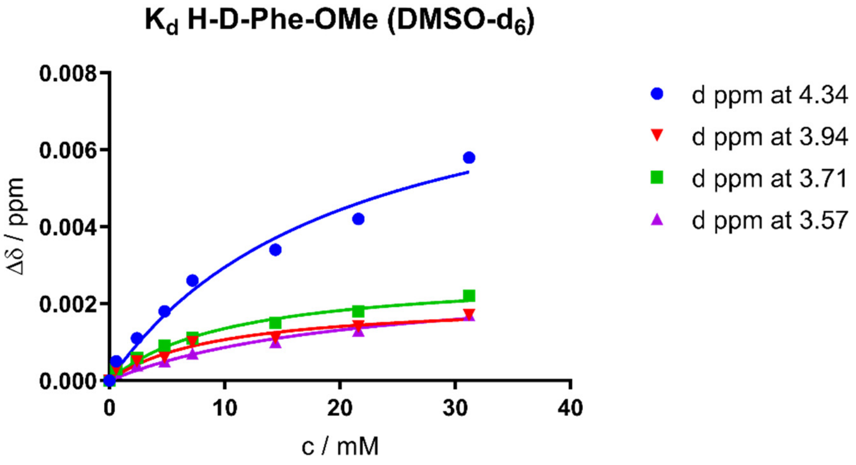

K_d values obtained by fitting the data for selected protons into the single-site specific binding model:

Kd	21.86	13.87	15.01	23.24
R squared	0.9795	0.9651	0.9833	0.978

K_d = 18.50 ± 4.10 mM → K_a = 54 ± 12 M^{-1}

H-L-Pro-OMe x HCl to receptor 1:

^{1}H NMR spectra:

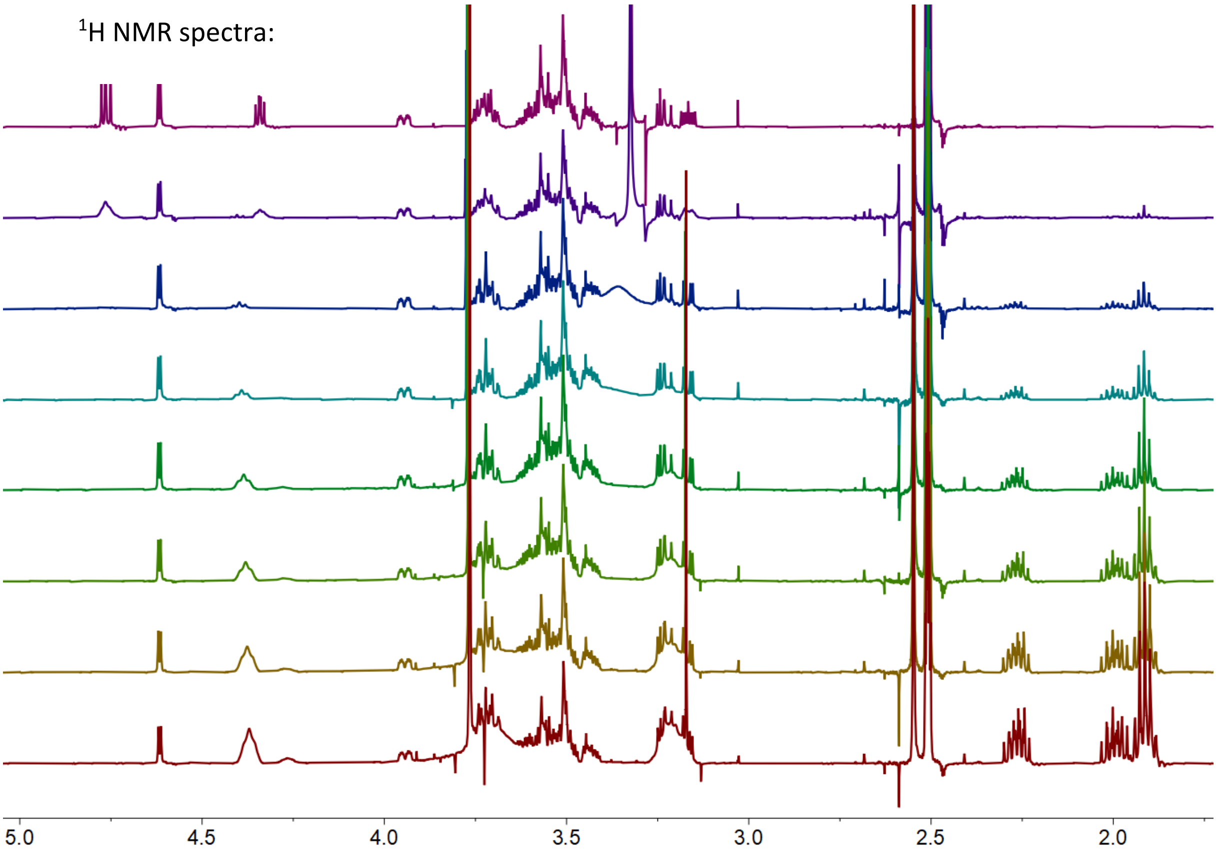

Summary of chemical shift changes:

Titration	4.62	Δppm	3.94	Δppm	3.71	Δppm	3.69	Δppm
0	4.6177	0.0000	3.9463	0.0000	3.711	0.0000	3.6885	0.0000
1	4.6174	0.0003	3.9459	0.0004	3.7105	0.0005	3.6881	0.0004
2	4.6172	0.0005	3.9455	0.0008	3.7099	0.0011	3.6879	0.0006
3	4.6171	0.0006	3.945	0.0013	3.7095	0.0015	3.6876	0.0009
4	4.6169	0.0008	3.9447	0.0016	3.7088	0.0022	3.6874	0.0011
5	4.6167	0.0010	3.9444	0.0019	3.7083	0.0027	3.6866	0.0019
6	4.6164	0.0013	3.9435	0.0028	3.7077	0.0033	3.6862	0.0023
7	4.6162	0.0015	3.9433	0.0030	3.707	0.0040	3.686	0.0025

Plot of chemical shift change vs guest concentration:

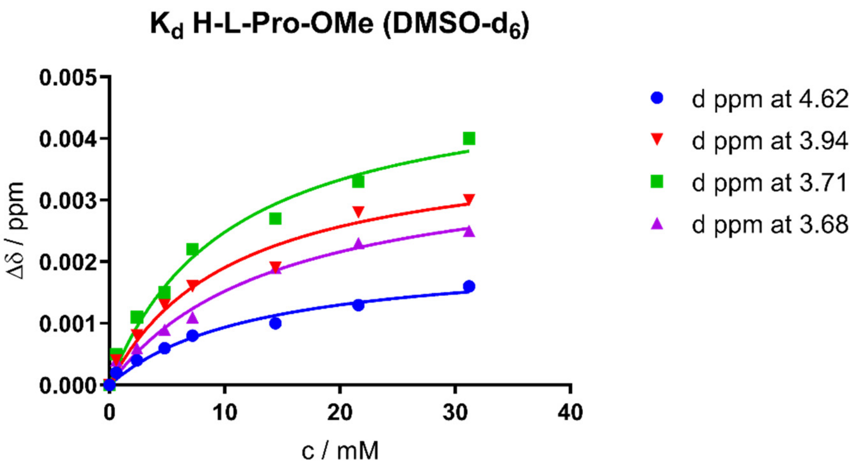

K_d values obtained by fitting the data for selected protons into the single-site specific binding model:

**Kd**	11.19	10.91	15.17	17.93
**R squared**	0.9798	0.9719	0.9867	0.9827

K_d = 13.80 ± 2.92 mM → K_a = 72 ± 15 M^{-1}

H-D-Pro-OMe x HCl to receptor 1:

^{1}H NMR spectra:

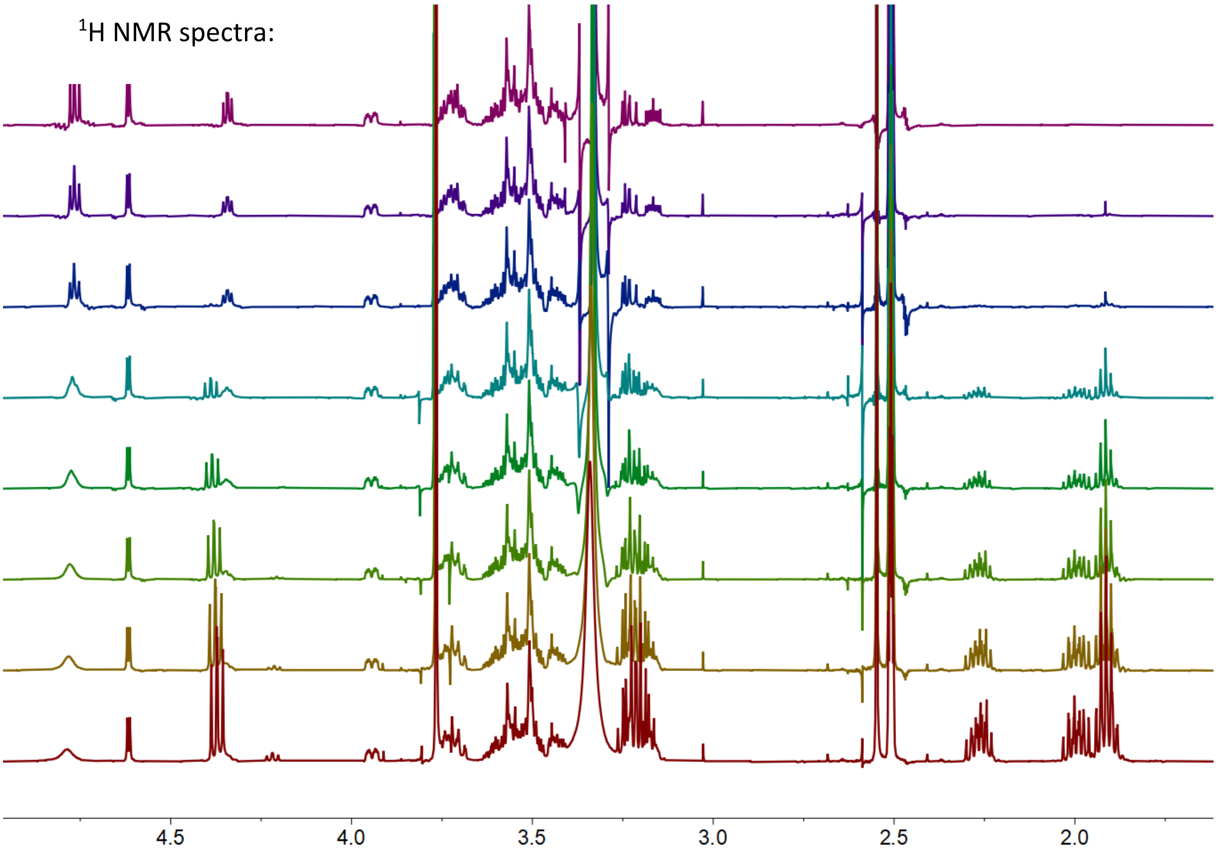

Summary of chemical shift changes:

Titration	4.76	Δppm	3.94	Δppm	3.68	Δppm	3.5	Δppm
0	4.7663	0.0000	3.9463	0.0000	3.688	0.0000	3.5045	0.0000
1	4.7668	0.0005	3.9461	0.0002	3.6878	0.0002	3.5043	0.0002
2	4.7672	0.0009	3.9457	0.0006	3.6876	0.0004	3.5042	0.0003
3	4.7716	0.0053	3.9454	0.0009	3.6875	0.0005	3.5040	0.0005
4	4.7747	0.0084	3.945	0.0013	3.6871	0.0009	3.5039	0.0006
5	4.7781	0.0118	3.9445	0.0018	3.6867	0.0013	3.5035	0.0010
6	4.7826	0.0163	3.9438	0.0025	3.6862	0.0018	3.5033	0.0012
7	4.7846	0.0183	3.943	0.0033	3.6857	0.0023	3.5028	0.0017

Plot of chemical shift change vs guest concentration:

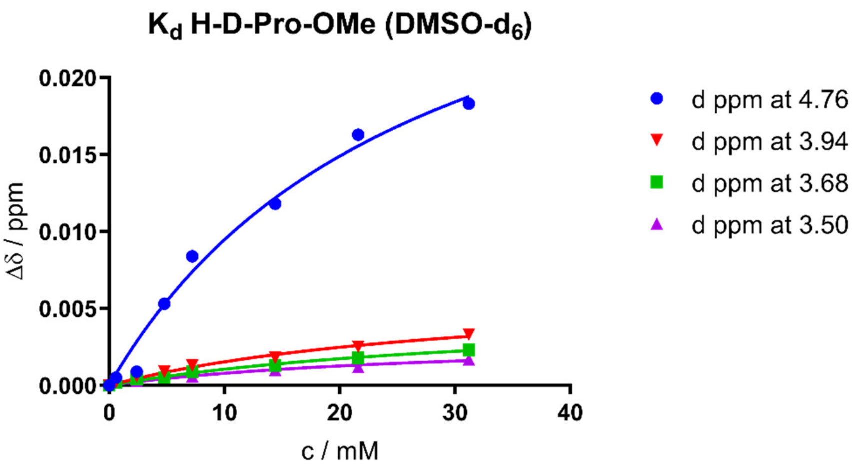

K_d values obtained by fitting the data for selected protons into the single-site specific binding model:

**Kd**	29.03	34.77	29.71	32.8
**R squared**	0.9833	0.9881	0.9922	0.9759

K_d = 31.58 ± 2.33 mM → K_a = 32 ± 2 M^{-1}

H-L-Thr-OMe x HCl to receptor 1:

^{1}H NMR spectra:

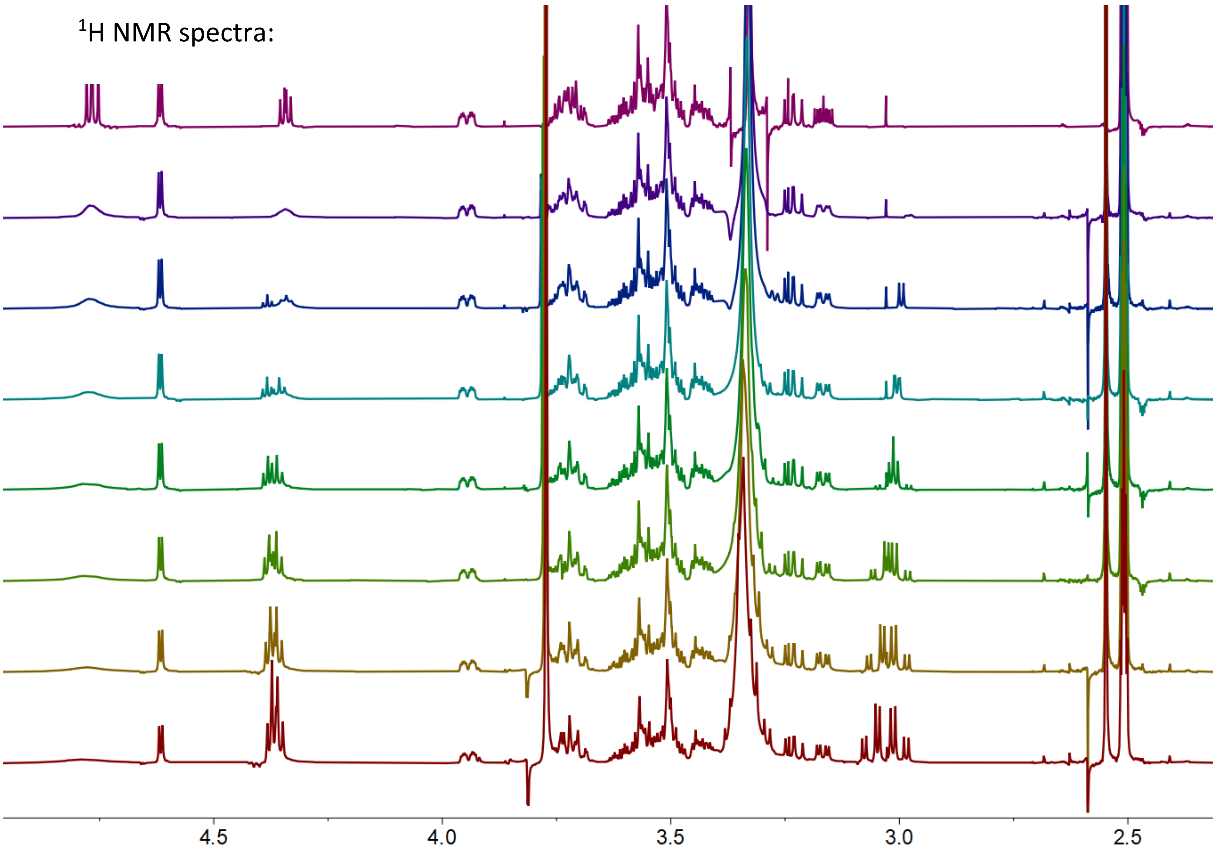

Summary of chemical shift changes:

Titration	4.61	Δppm	3.94	Δppm	3.71	Δppm	3.23	Δppm
0	4.6175	0.0000	3.9466	0.0000	3.7122	0.0000	3.2322	0.0000
1	4.6172	0.0003	3.9458	0.0008	3.7117	0.0005	3.232	0.0002
2	4.617	0.0005	3.9454	0.0012	3.7115	0.0007	3.2318	0.0004
3	4.6169	0.0006	3.9452	0.0014	3.7113	0.0009	3.2317	0.0005
4	4.6168	0.0007	3.9449	0.0017	3.7111	0.0011	3.2315	0.0007
5	4.6165	0.0010	3.9444	0.0022	3.7108	0.0014	3.2312	0.0010
6	4.6162	0.0013	3.9435	0.0031	3.7104	0.0018	3.2309	0.0013
7	4.616	0.0015	3.9433	0.0033	3.7099	0.0023	3.2306	0.0016

Plot of chemical shift change vs guest concentration:

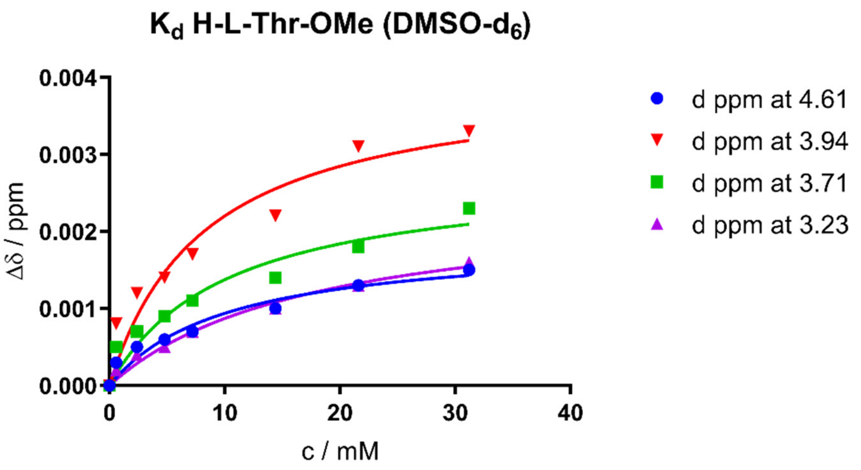

K_d values obtained by fitting the data for selected protons into the single-site specific binding model:

**Kd**	10.6	8.55	10.58	8.81
**R squared**	0.954	0.9358	0.9337	0.9829

K_d = 9.64 ± 0.96 mM → K_a = 104 ± 10 M^{-1}

H-D-Thr-OMe x HCl to receptor 1:

^{1}H NMR spectra:

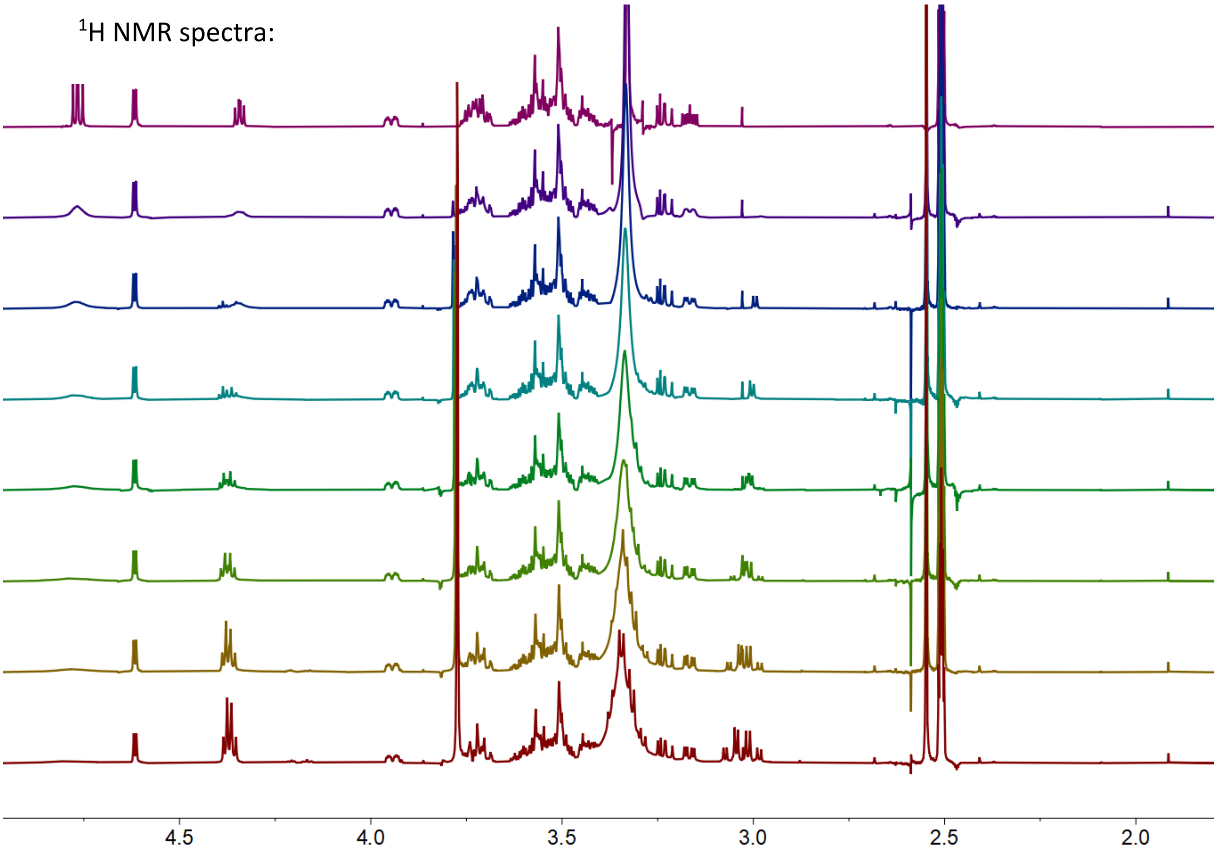

Summary of chemical shift changes:

Titration	4.62	Δppm	3.94	Δppm	3.71	Δppm	3.23	Δppm
0	4.6174	0.0000	3.9466	0.0000	3.7106	0.0000	3.2322	0.0000
1	4.6172	0.0002	3.9462	0.0004	3.7099	0.0007	3.232	0.0002
2	4.6171	0.0003	3.9458	0.0008	3.7098	0.0008	3.2318	0.0004
3	4.617	0.0004	3.9453	0.0013	3.7095	0.0011	3.2317	0.0005
4	4.6168	0.0006	3.945	0.0016	3.709	0.0016	3.2314	0.0008
5	4.6165	0.0009	3.9444	0.0022	3.7086	0.0020	3.2312	0.0010
6	4.6163	0.0011	3.9438	0.0028	3.7081	0.0025	3.2308	0.0014
7	4.616	0.0014	3.9431	0.0035	3.7073	0.0033	3.2304	0.0018

Plot of chemical shift change vs guest concentration:

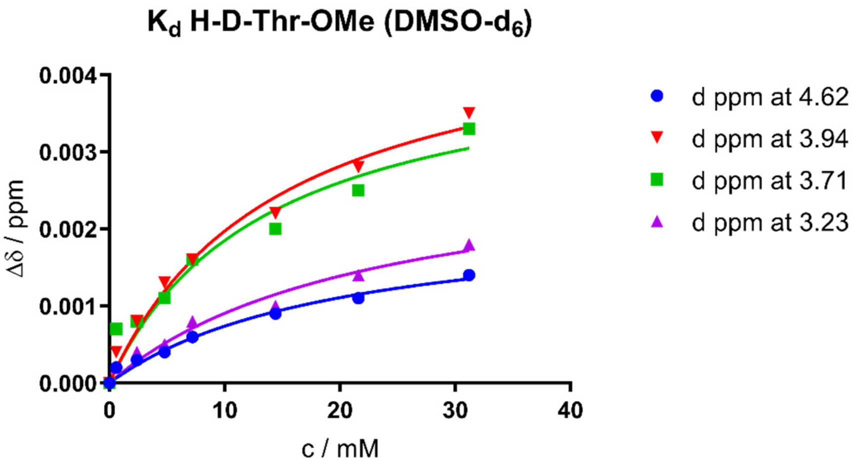

K_d values obtained by fitting the data for selected protons into the single-site specific binding model:

**Kd**	21.28	15.71	14.34	14.3
**R squared**	0.9839	0.9827	0.9457	0.9763

K_d = 16.40 ± 2.87 mM → K_a = 61 ± 11 M^{-1}

H-L-Val-OMe x HCl to receptor 1:

^{1}H NMR spectra:

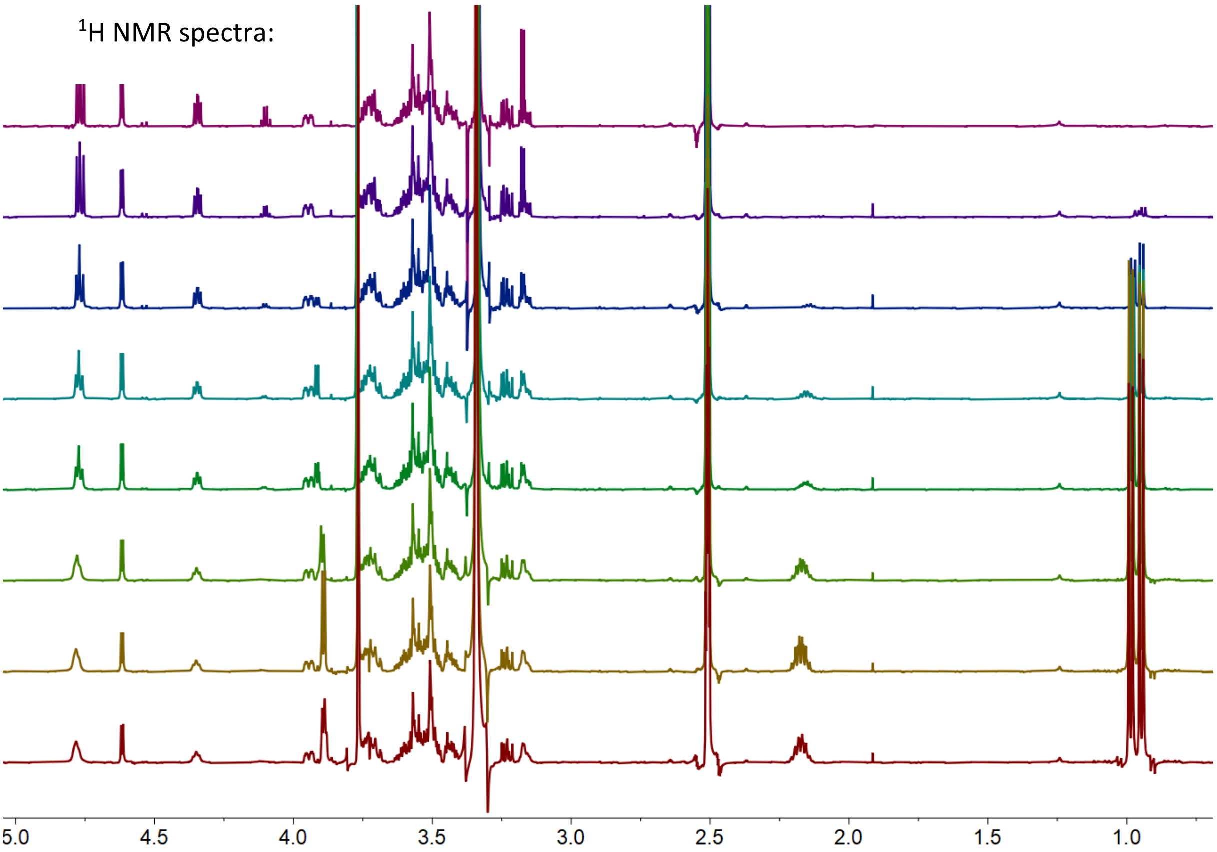

Summary of chemical shift changes:

Titration	4.34	Δppm	3.94	Δppm	3.59	Δppm	3.54	Δppm
0	4.3444	0.0000	3.9467	0.0000	3.5944	0.0000	3.5415	0.0000
1	4.3453	0.0009	3.9464	0.0003	3.5939	0.0005	3.5409	0.0006
2	4.3459	0.0015	3.9458	0.0009	3.5937	0.0007	3.5404	0.0011
3	4.3464	0.0020	3.9457	0.0010	3.5935	0.0009	3.5402	0.0013
4	4.3466	0.0022	3.9454	0.0013	3.5933	0.0011	3.5401	0.0014
5	4.3486	0.0042	3.9448	0.0019	3.593	0.0014	3.5400	0.0015
6	4.3488	0.0044	3.9446	0.0021	3.5929	0.0015	3.5397	0.0018
7	4.3489	0.0045	3.9445	0.0022	3.5929	0.0015	3.5395	0.0020

Plot of chemical shift change vs guest concentration:

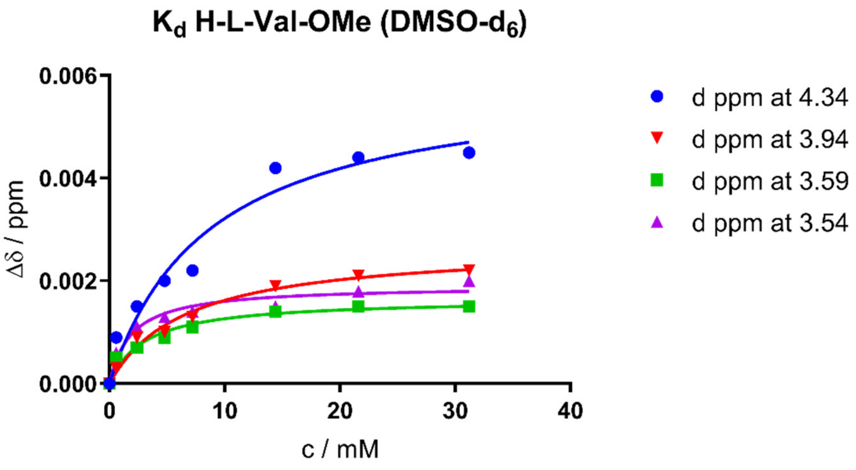

K_d values obtained by fitting the data for selected protons into the single-site specific binding model:

**Kd**	8.04	6.37	3.76	3.04
**R squared**	0.958	0.9876	0.9676	0.9627

K_d = 5.30 ± 2.00 mM → K_a = 189 ± 72 M^{-1}

H-D-Val-OMe x HCl to receptor 1:

^{1}H NMR spectra:

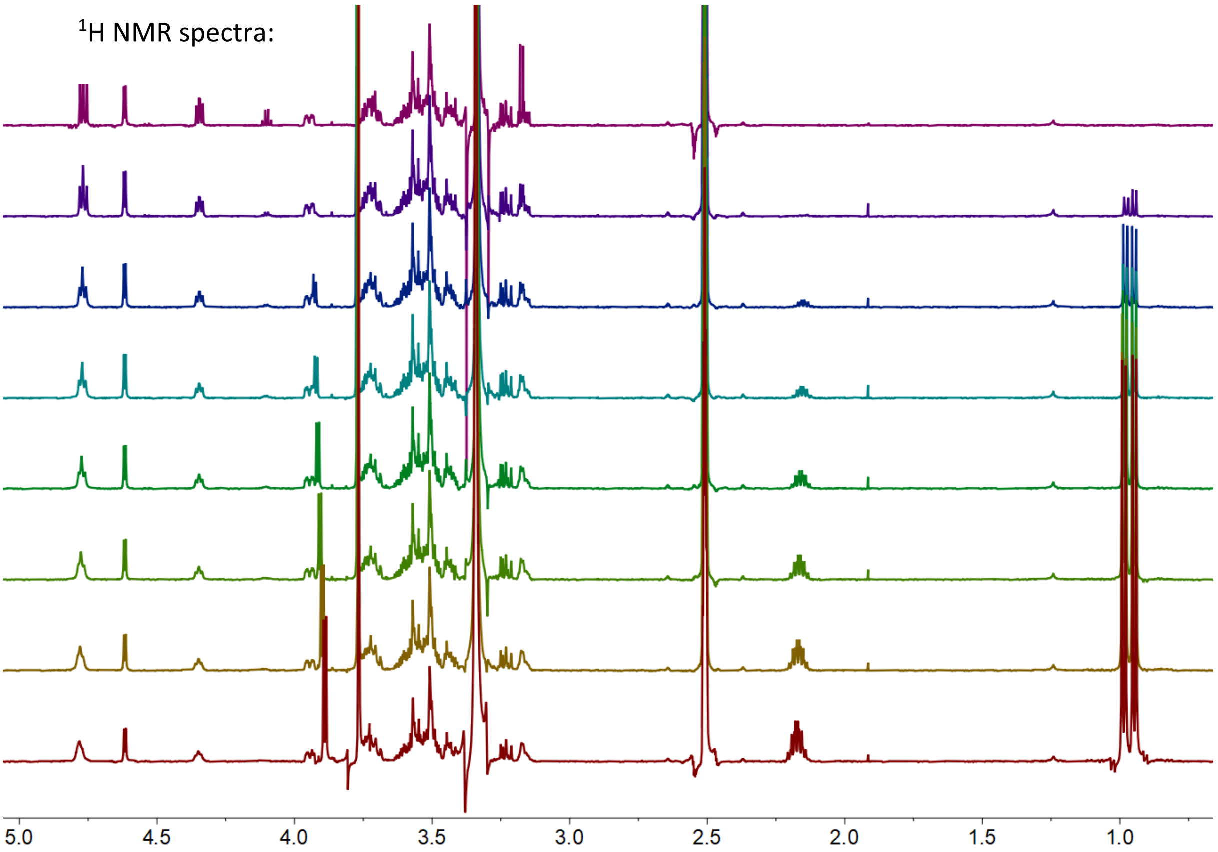

Summary of chemical shift changes:

Titration	4.76	Δppm	4.34	Δppm	3.94	Δppm	3.71	Δppm
0	4.768	0.0000	4.3448	0.0000	3.9465	0.0000	3.7074	0.0000
1	4.7687	0.0007	4.3451	0.0003	3.9463	0.0002	3.7071	0.0003
2	4.7706	0.0026	4.3455	0.0007	3.9457	0.0008	3.7068	0.0006
3	4.7719	0.0039	4.3459	0.0011	3.9455	0.0010	3.7065	0.0009
4	4.7739	0.0059	4.3469	0.0021	3.9452	0.0013	3.7062	0.0012
5	4.7764	0.0084	4.3477	0.0029	3.9448	0.0017	3.7058	0.0016
6	4.779	0.0110	4.3488	0.0040	3.9443	0.0022	3.7053	0.0021
7	4.7807	0.0127	4.3494	0.0046	3.9439	0.0026	3.7048	0.0026

Plot of chemical shift change vs guest concentration:

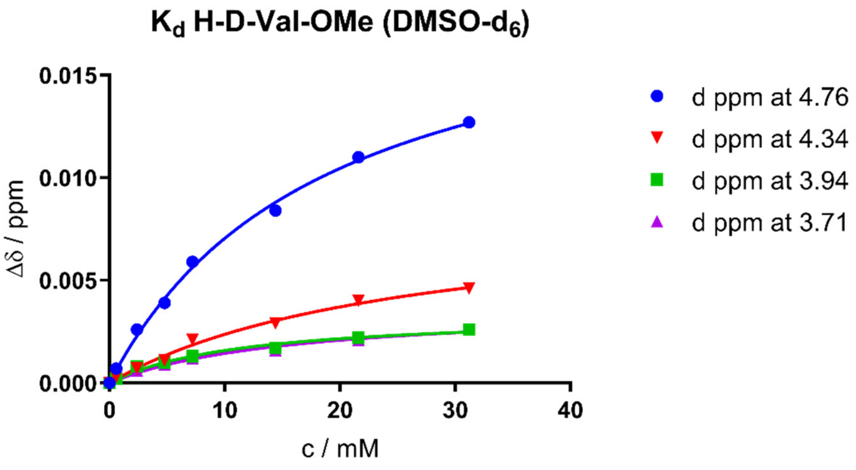

K_d values obtained by fitting the data for selected protons into the single-site specific binding model:

**Kd**	19.9	25.62	13.94	16.93
**R squared**	0.9982	0.9948	0.9838	0.9835

K_d = 19.10 ± 4.32 mM → K_a = 52 ± 12 M^{-1}

5. NMR spectra – receptor 1.

$CDCl_3$: ^{1}H NMR spectrum

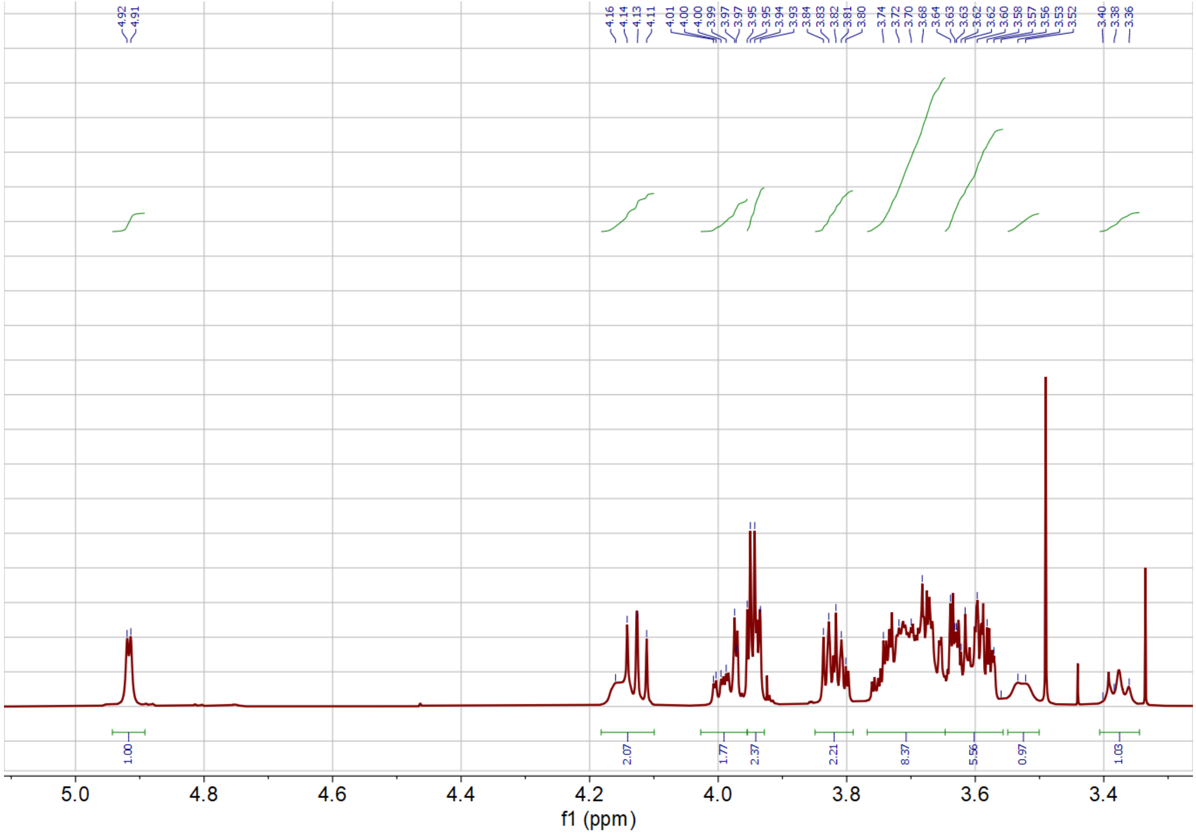

^{13}C NMR spectrum

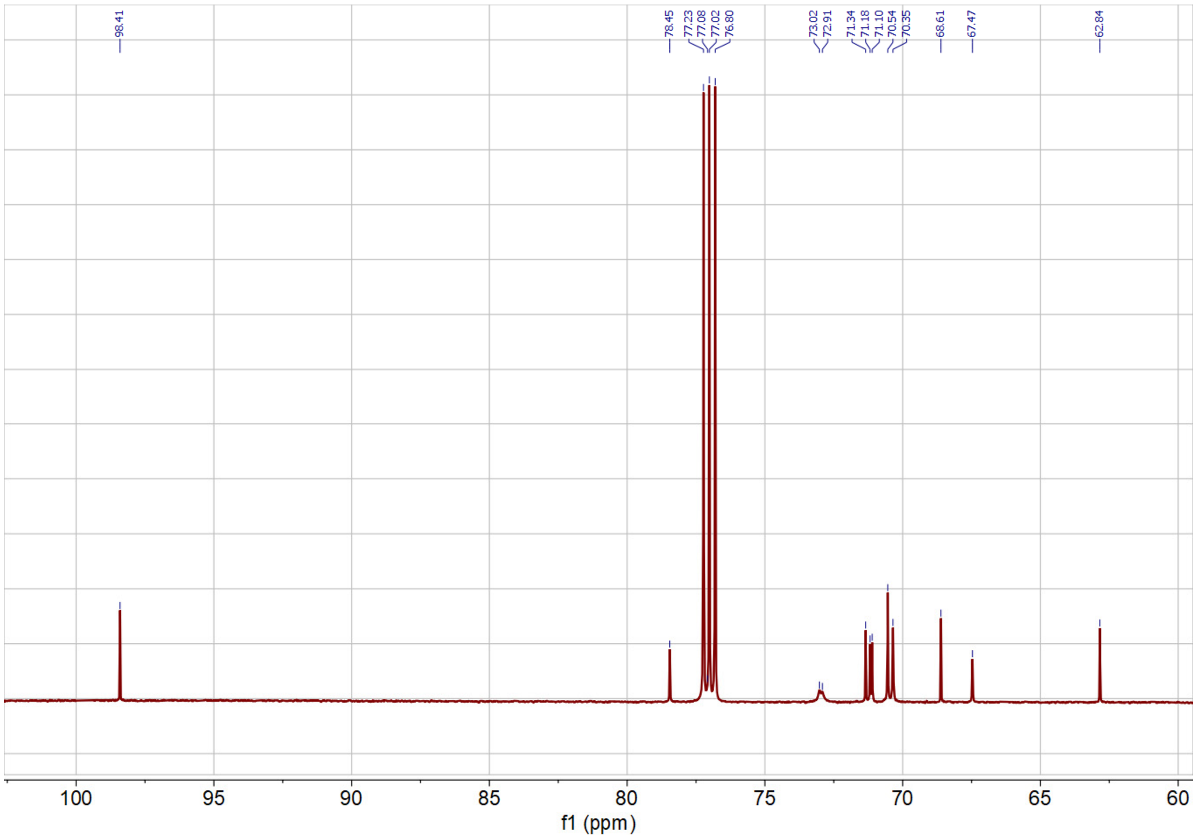

2D COSY spectrum

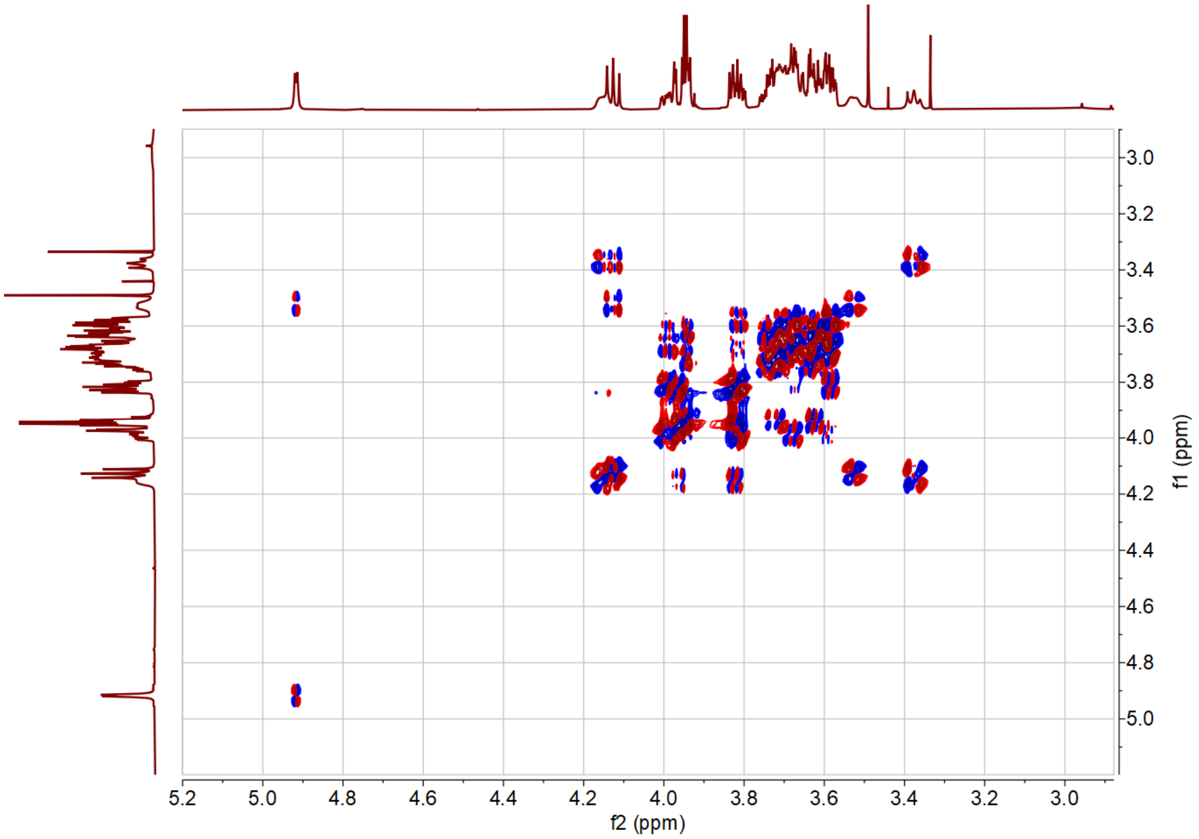

2D HSQC spectrum

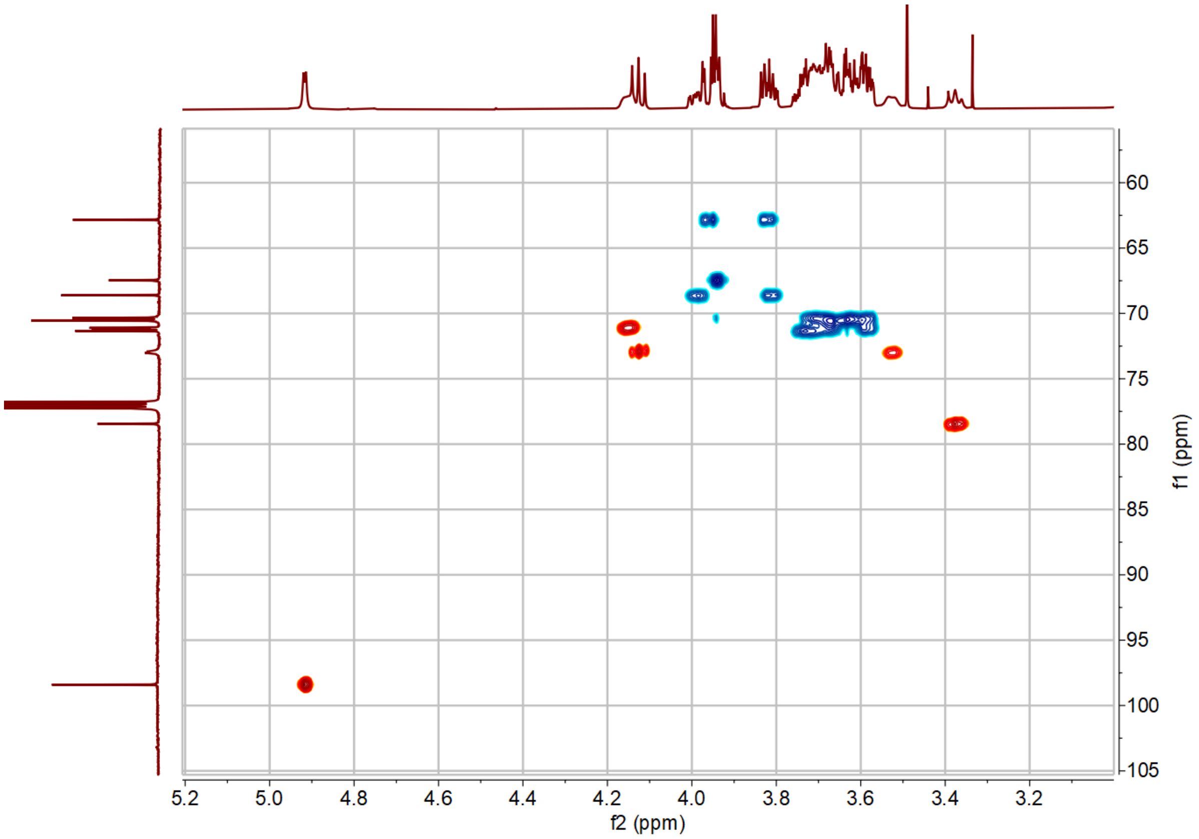

2D HMBC spectrum

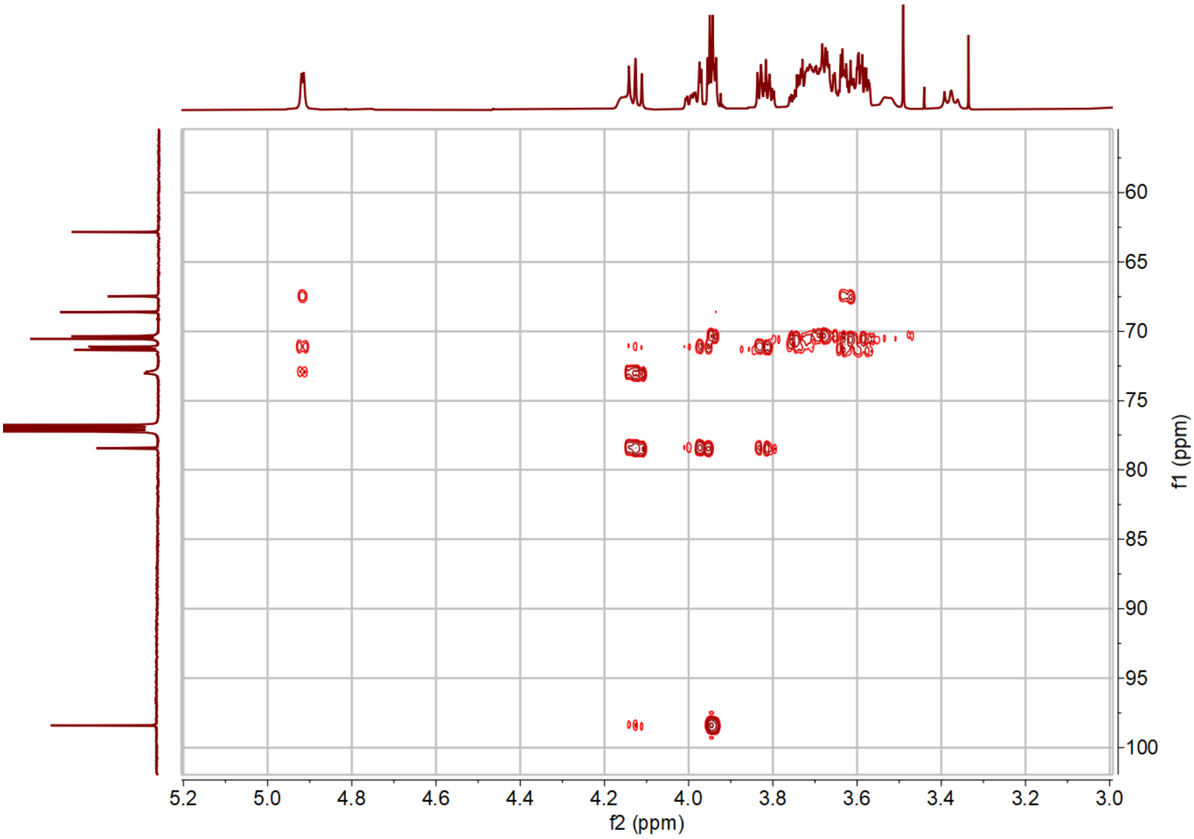

2D NOESY spectrum

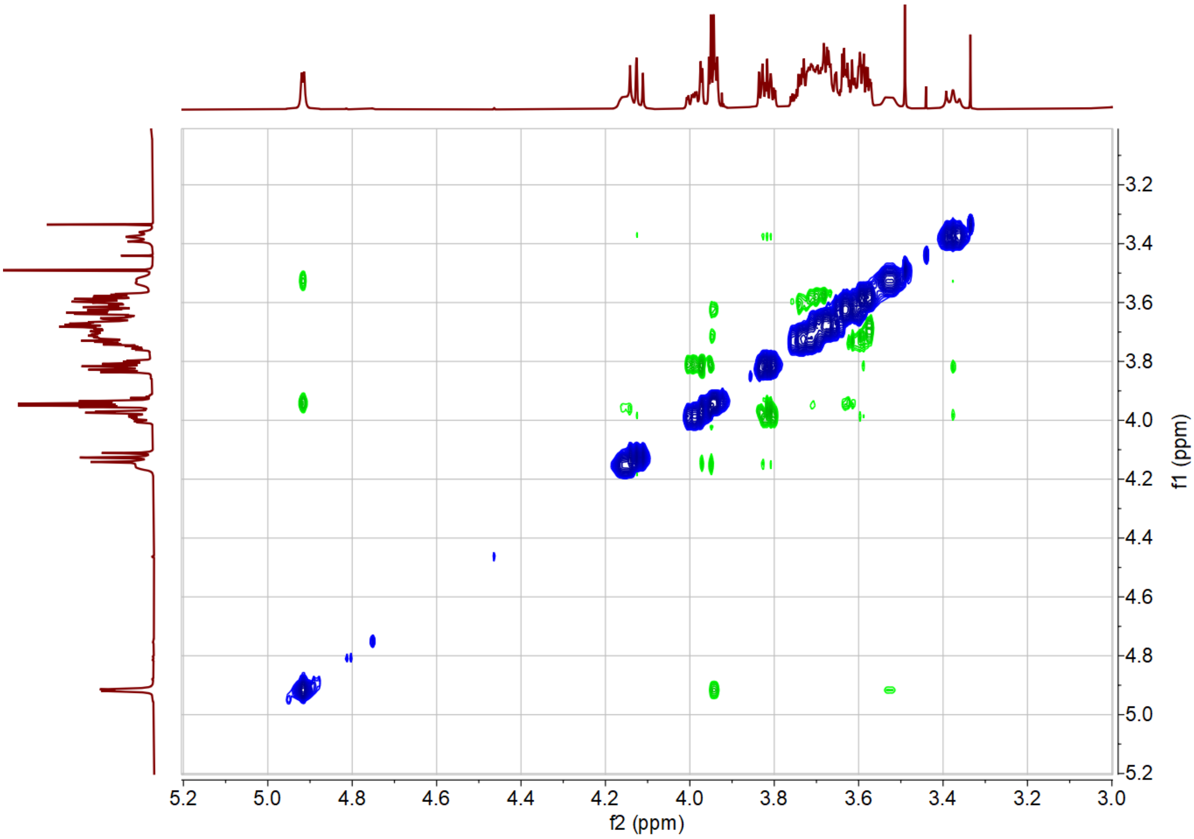

CD₃CN: ¹H NMR spectrum

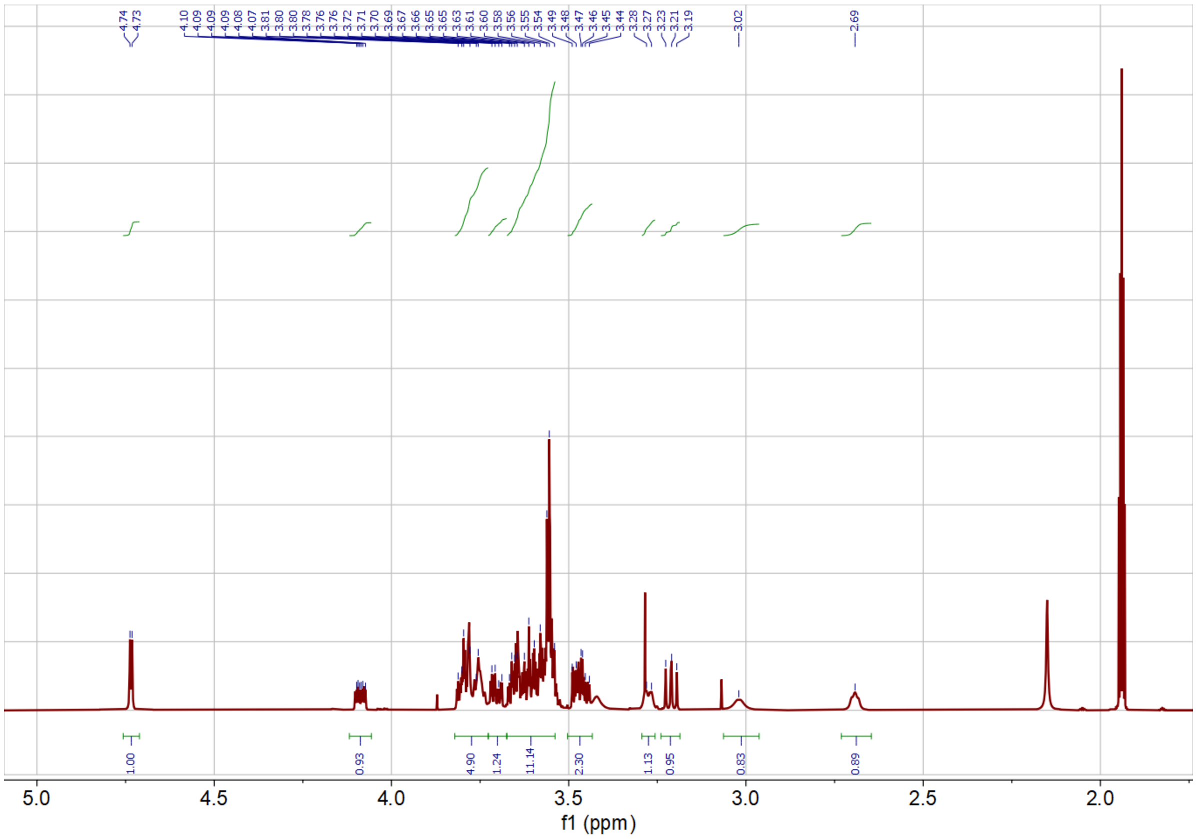

¹³C NMR spectrum

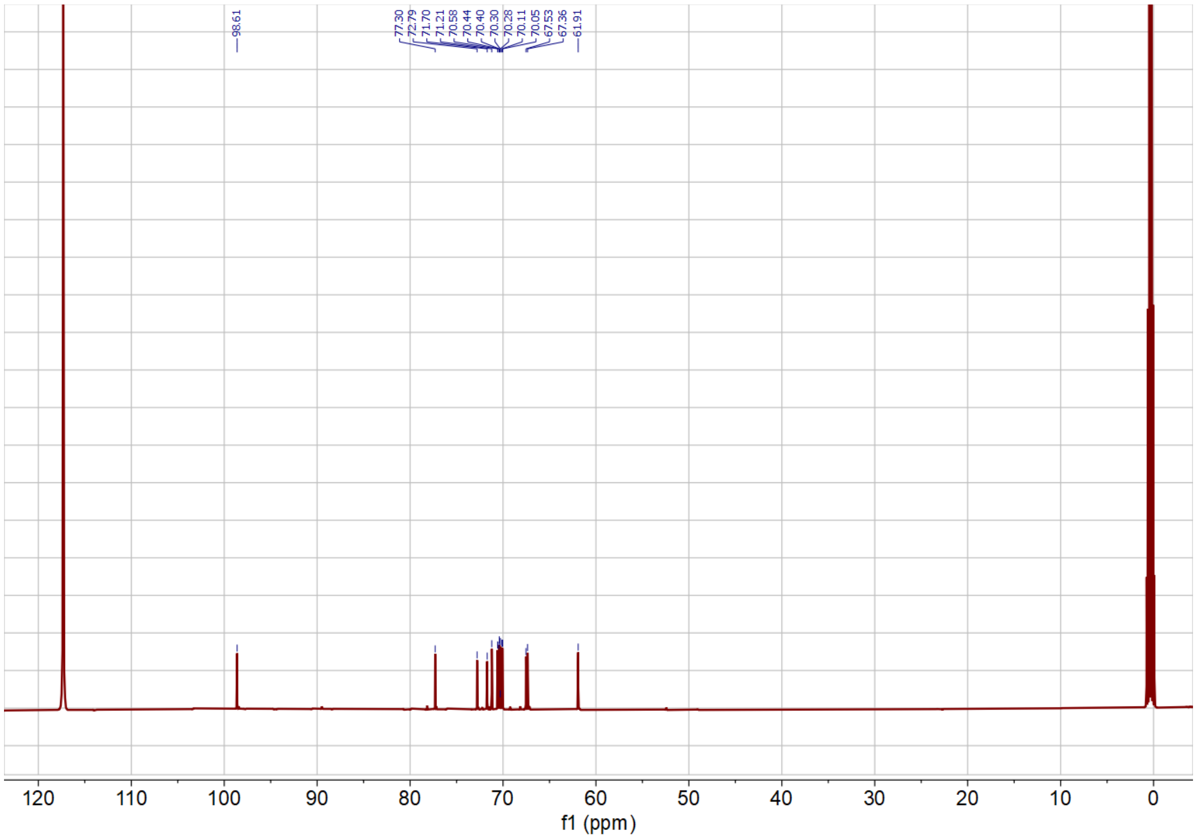

2D COSY spectrum

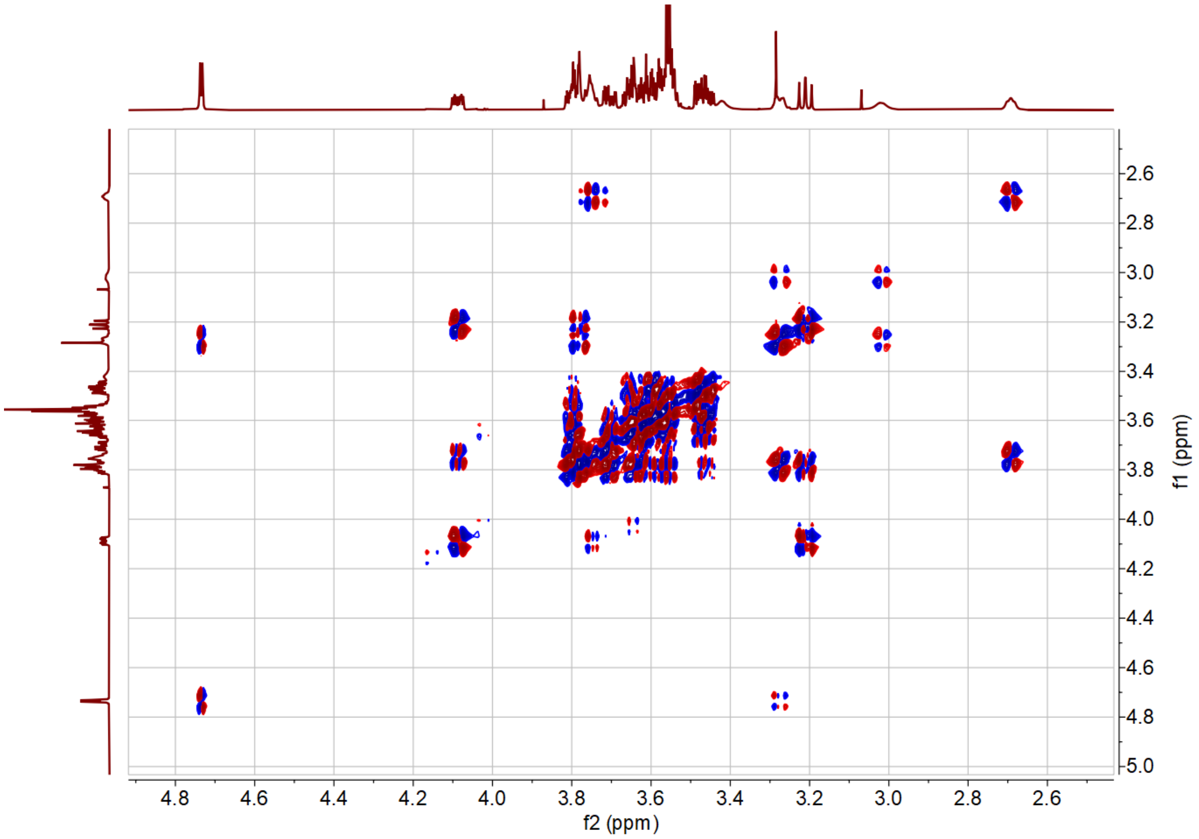

2D HSQC spectrum

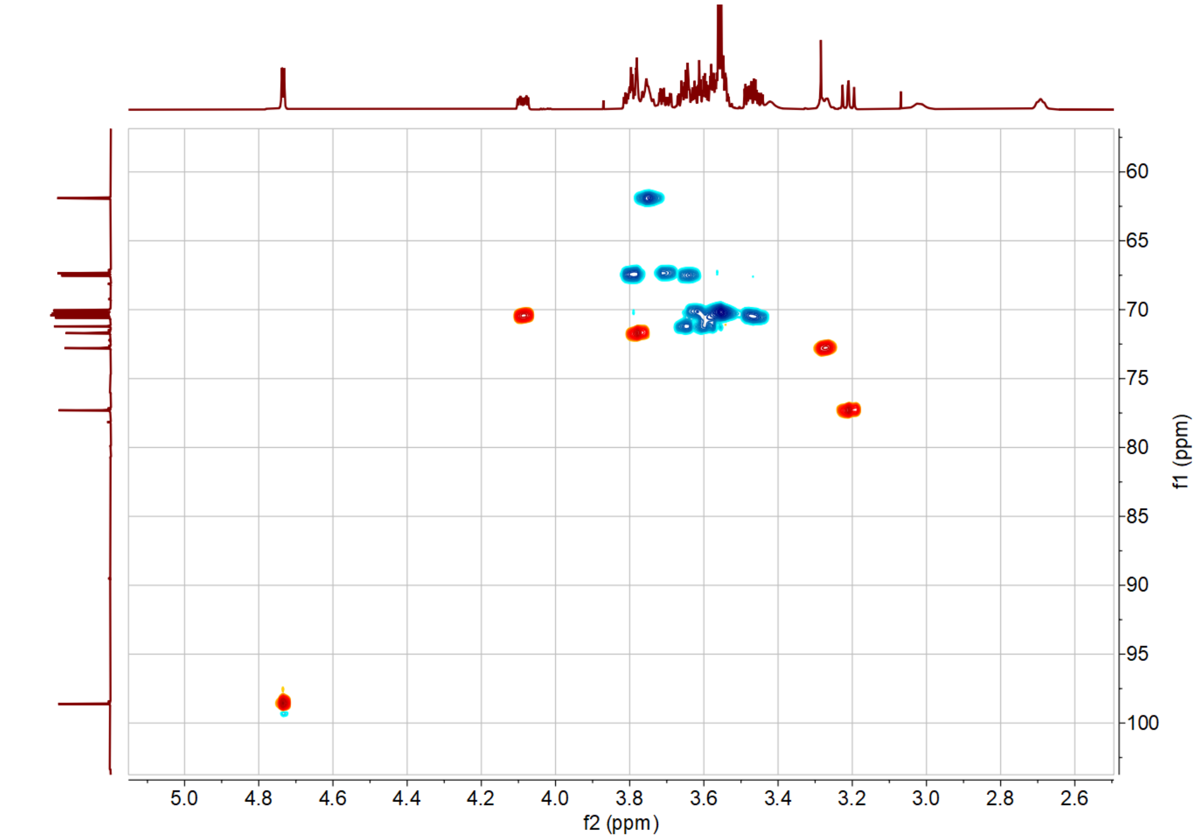

2D HMBC spectrum

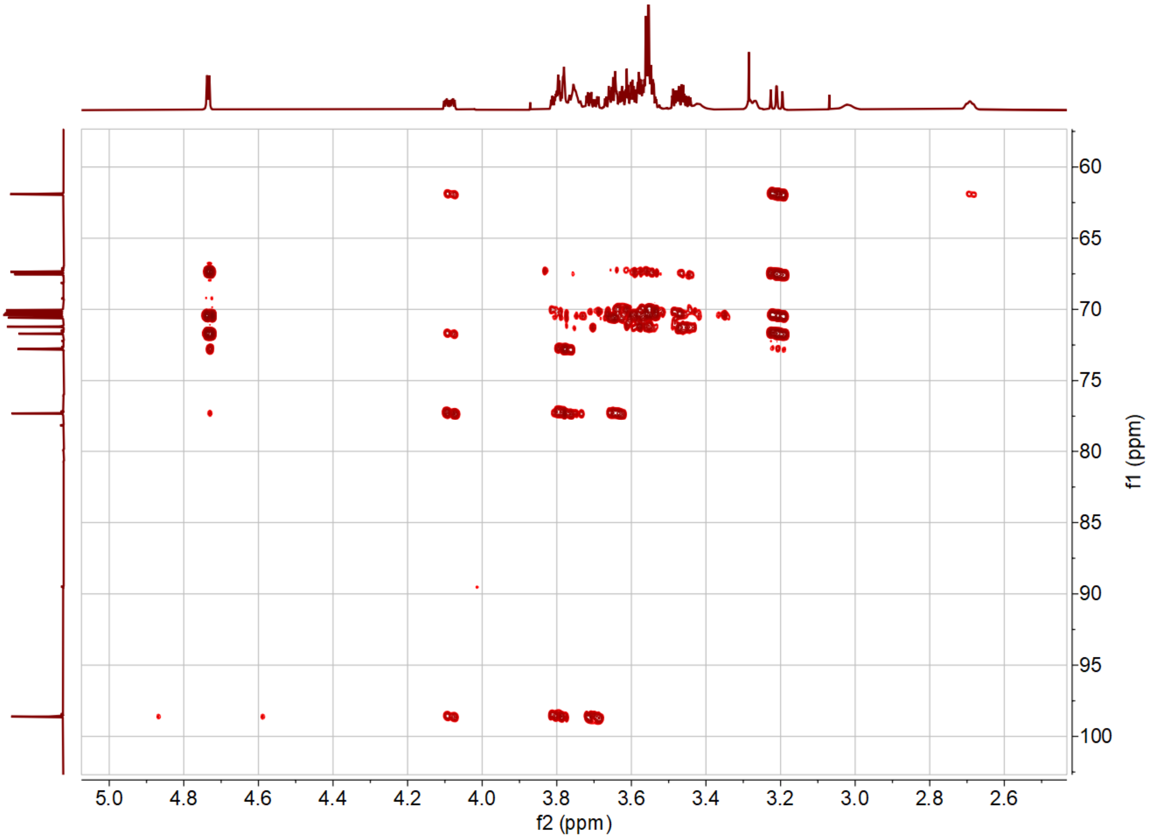

DMSO-d$_6$: ^{1}H NMR spectrum

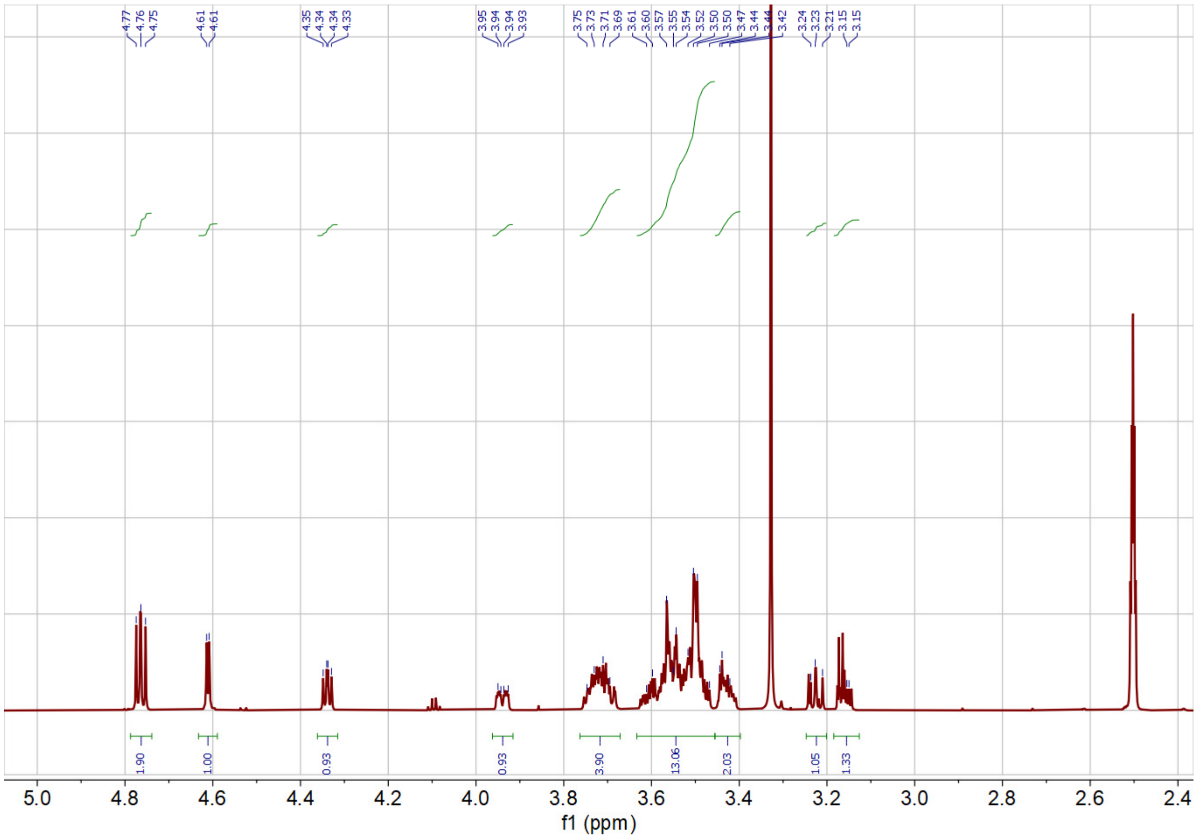

^{13}C NMR spectrum

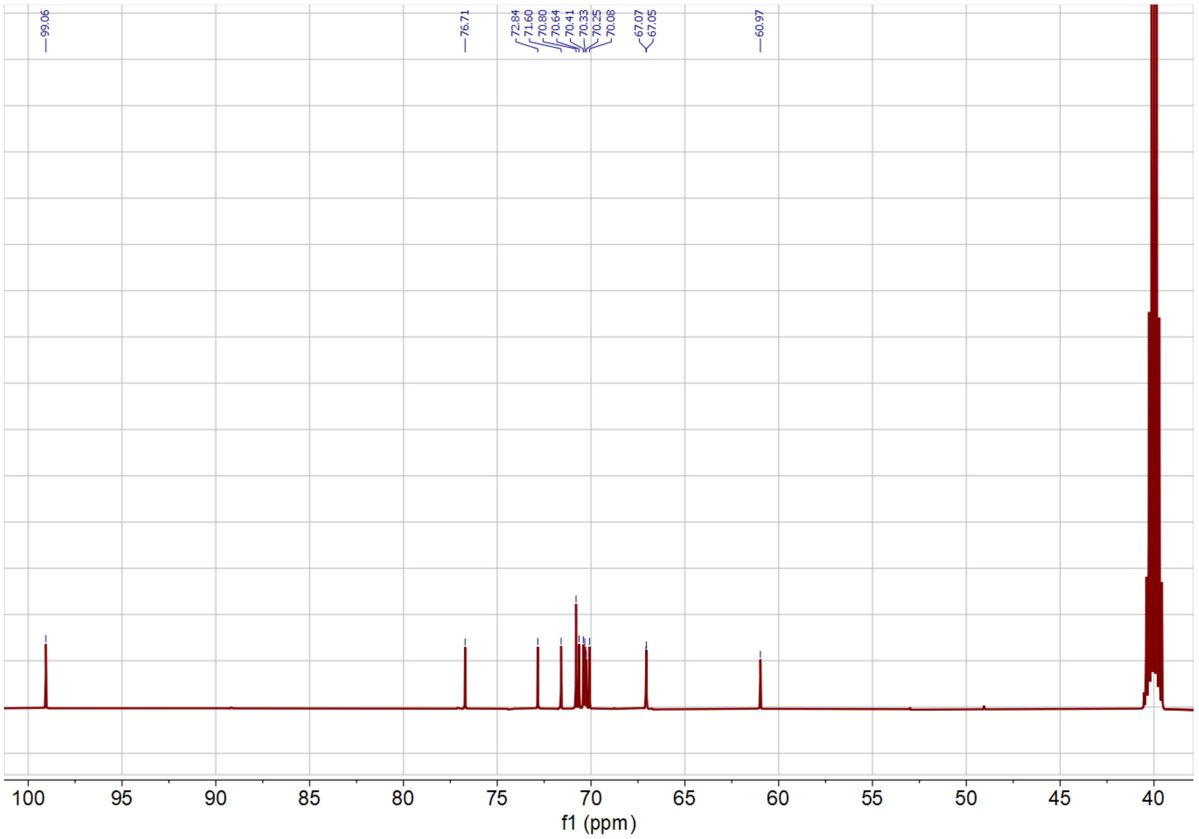

2D COSY spectrum

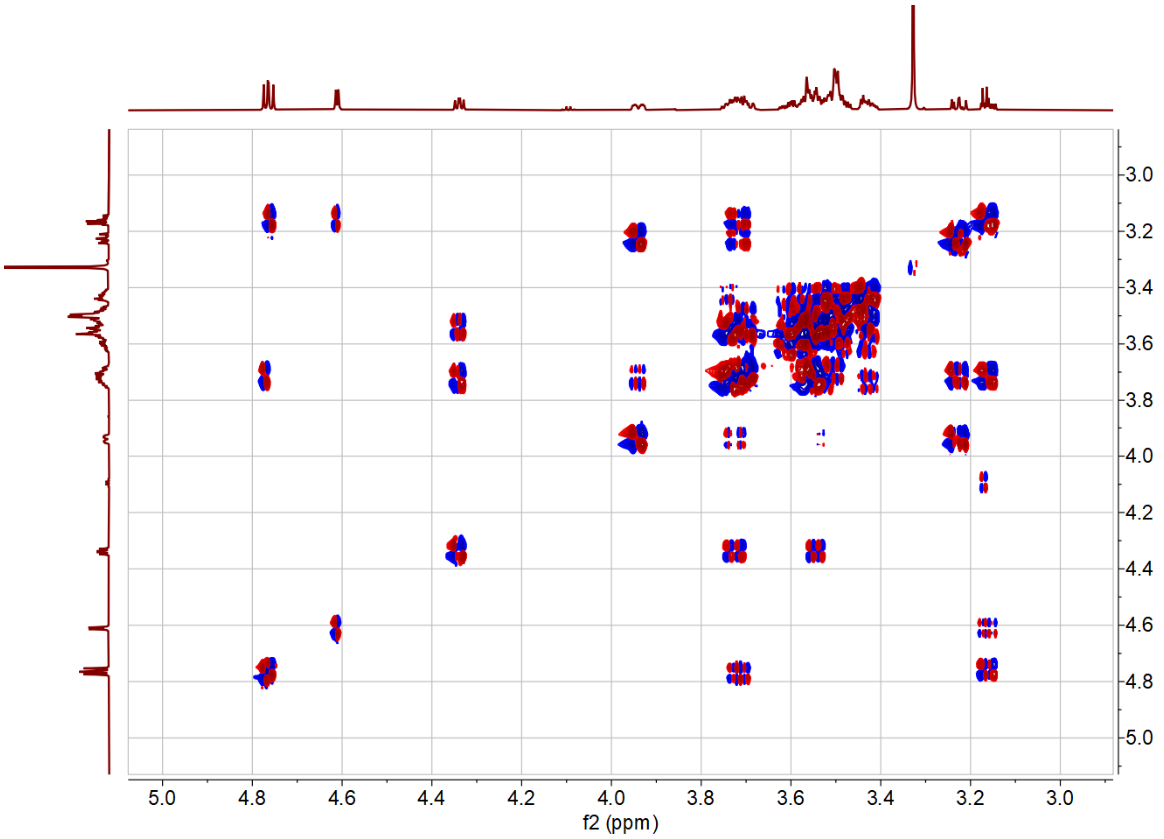

2D HSQC spectrum

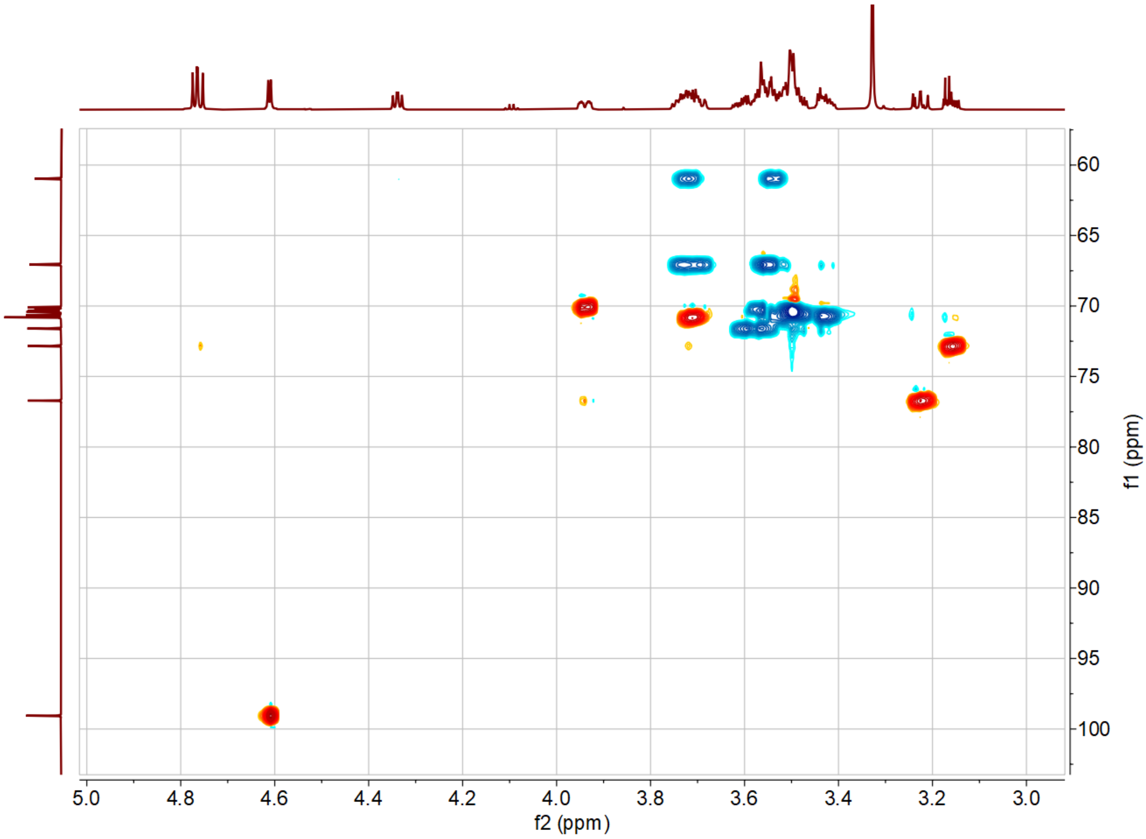

2D HMBC spectrum

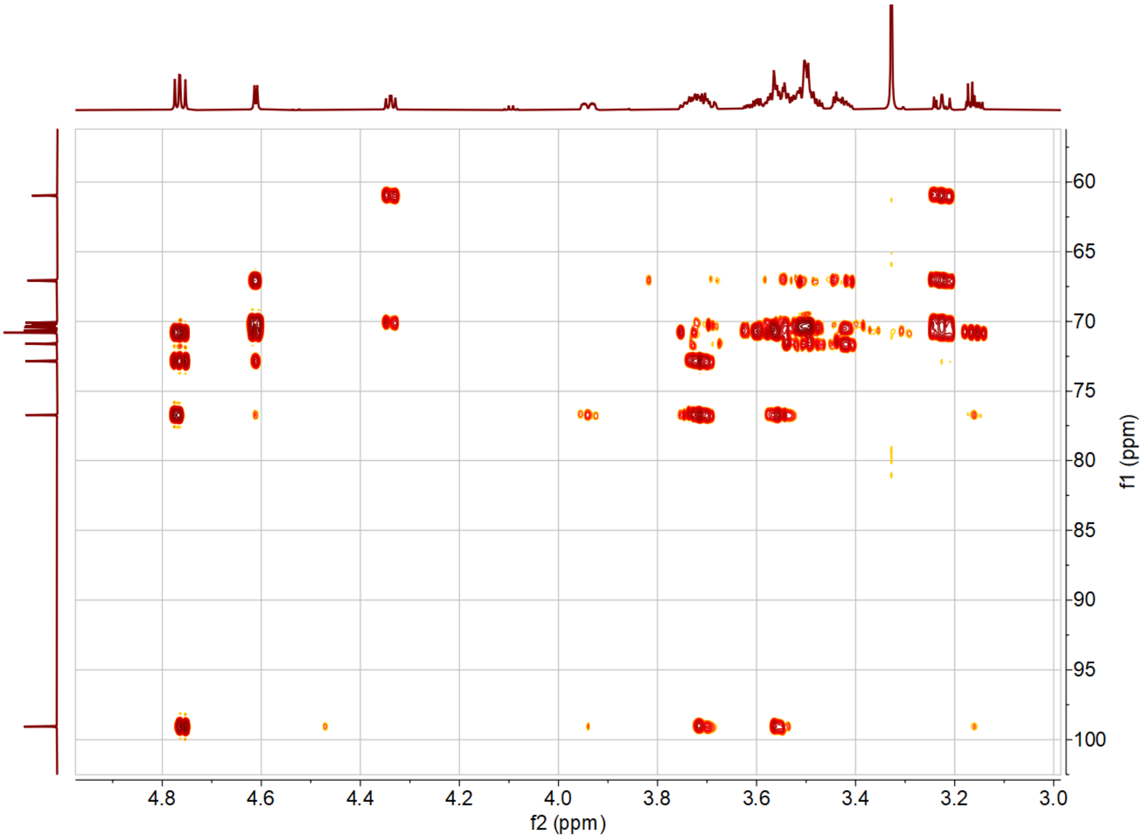

6. NMR spectra of 1 + H-Val-OMe complexes in $CDCl_3$.

1+H-L-Val-OMe: ^{1}H NMR spectrum

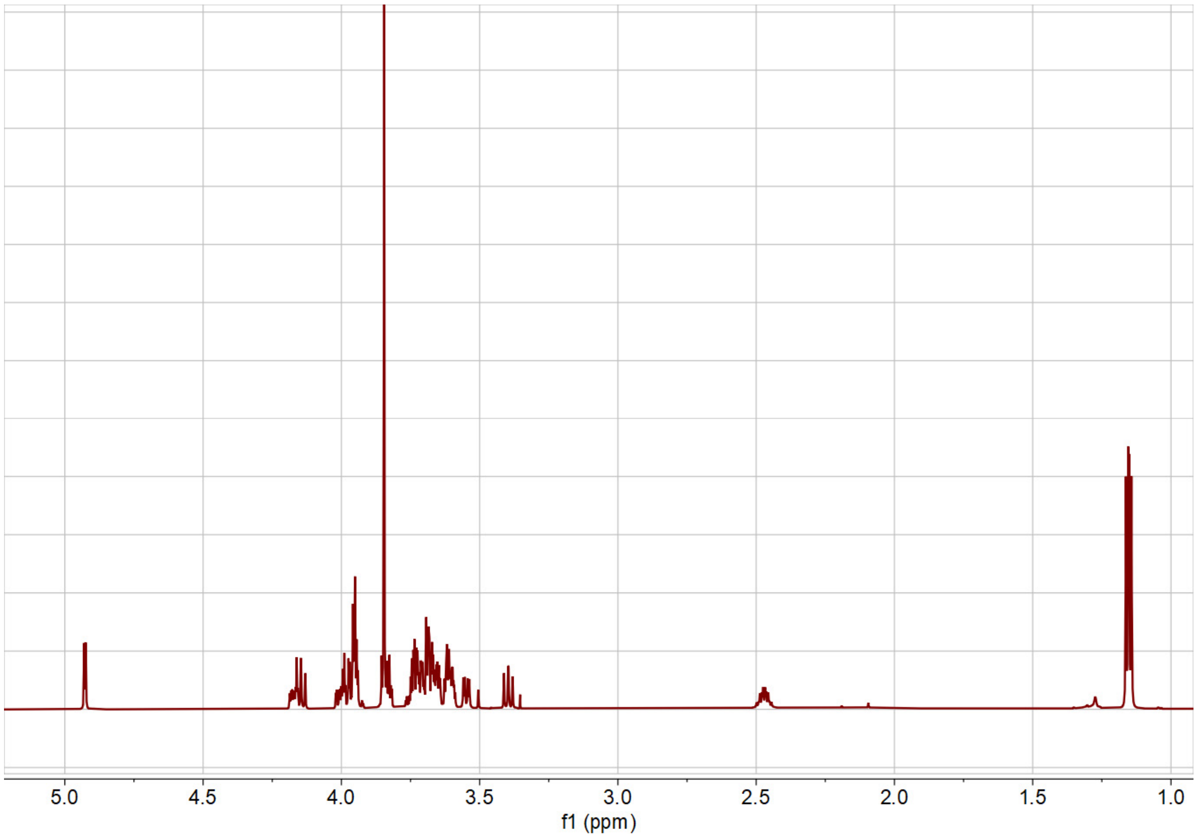

2D COSY NMR spectrum

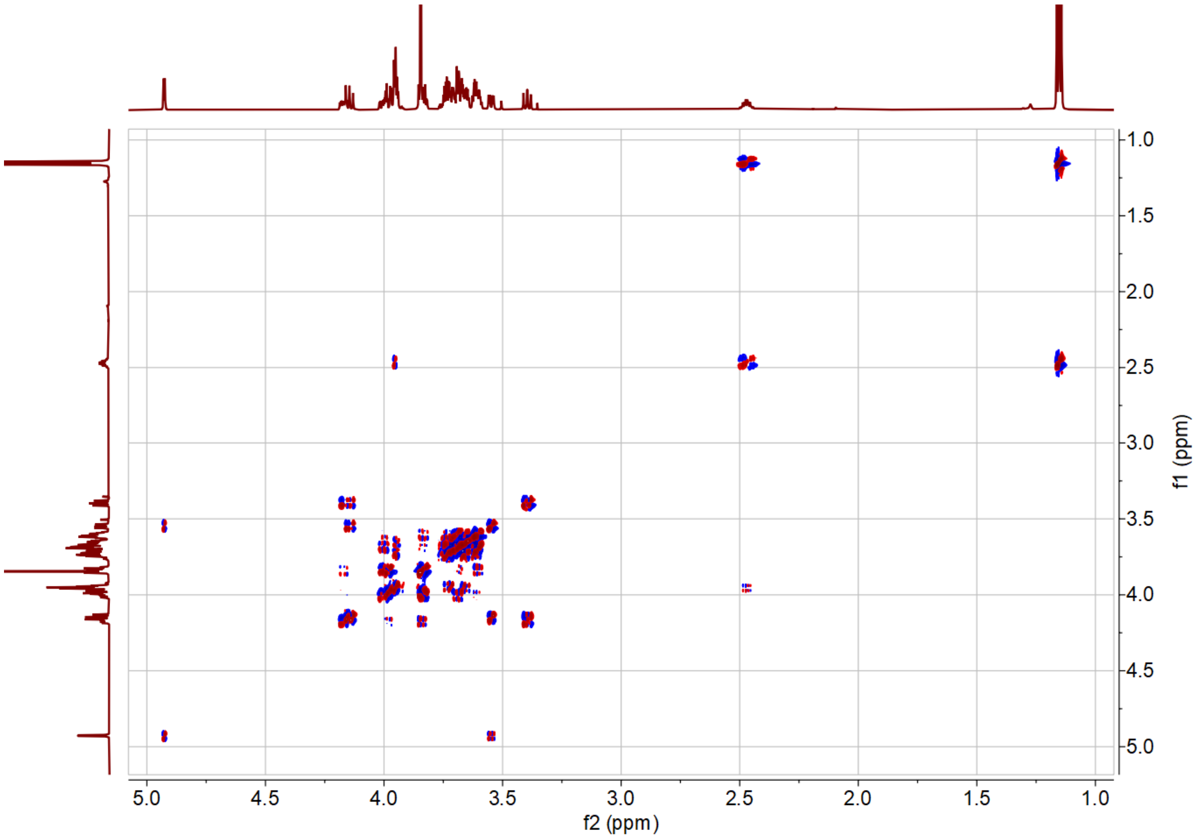

2D NOESY spectrum

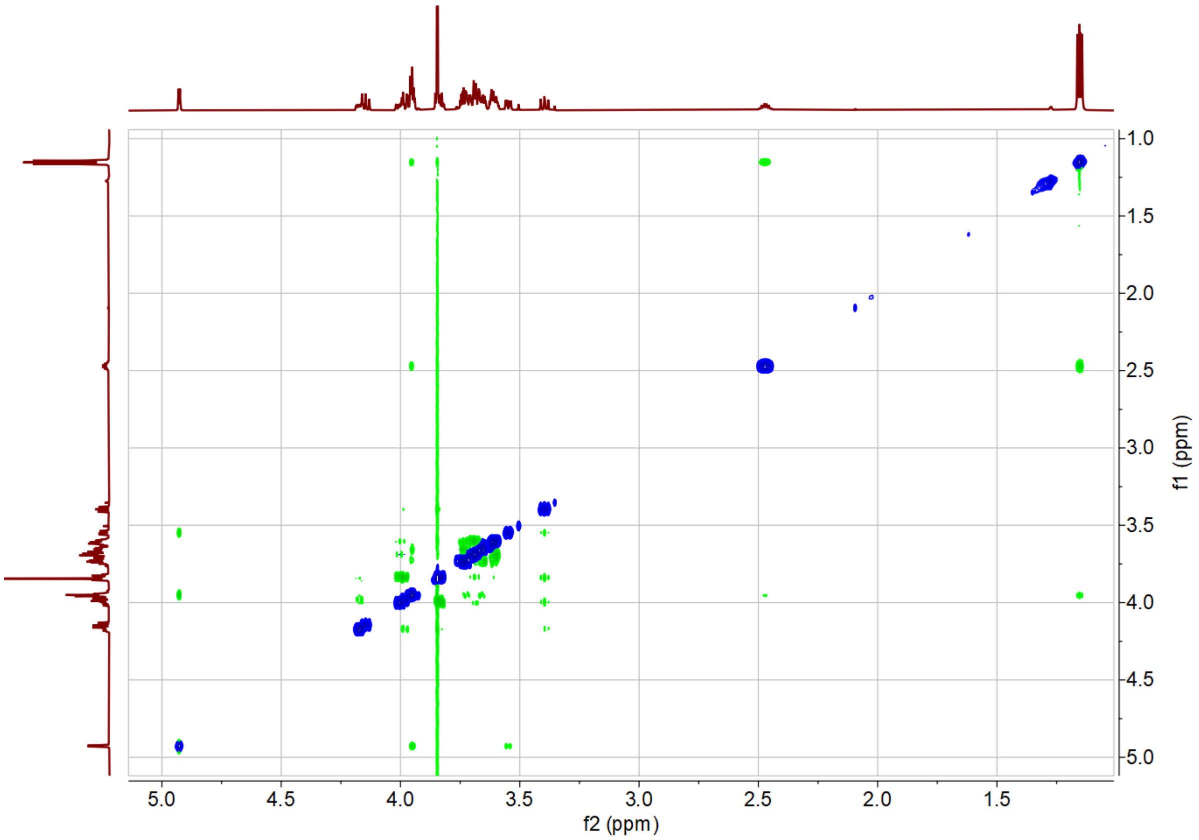

1+H-D-Val-OMe: ^{1}H NMR spectrum

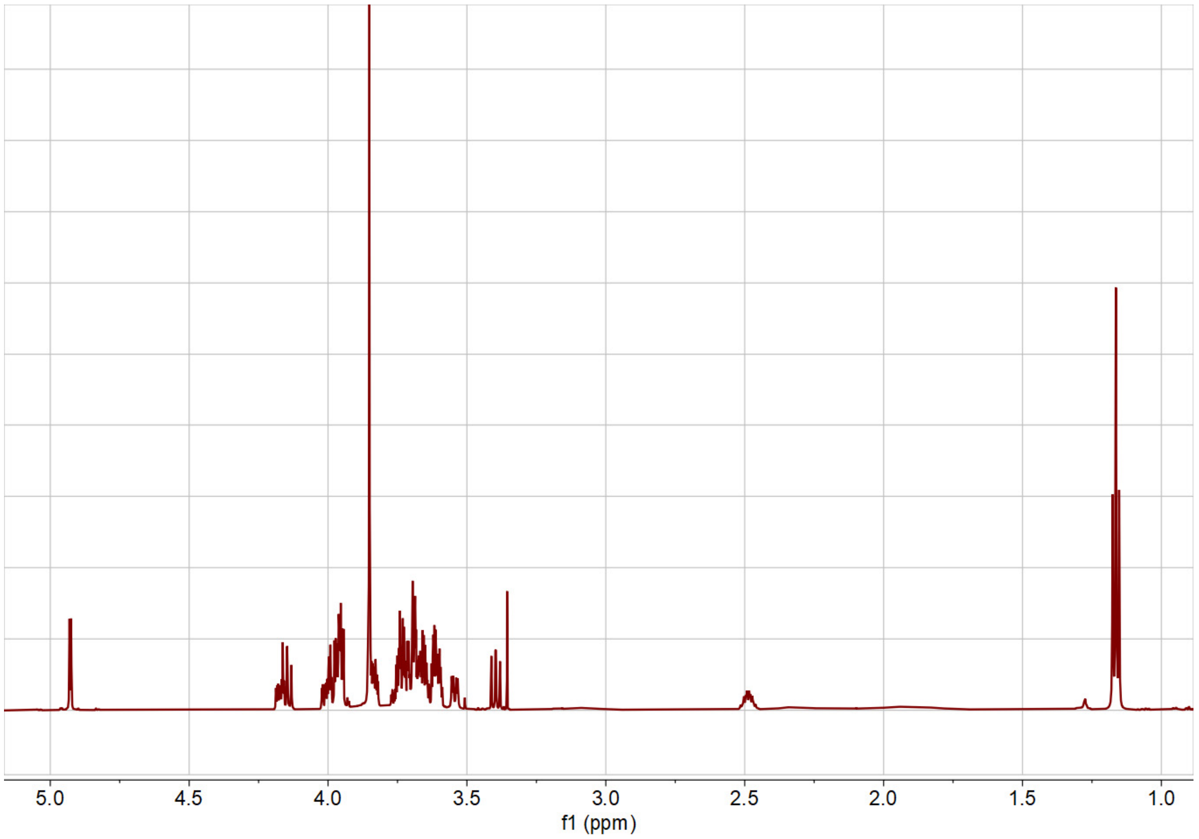

2D COSY NMR spectrum

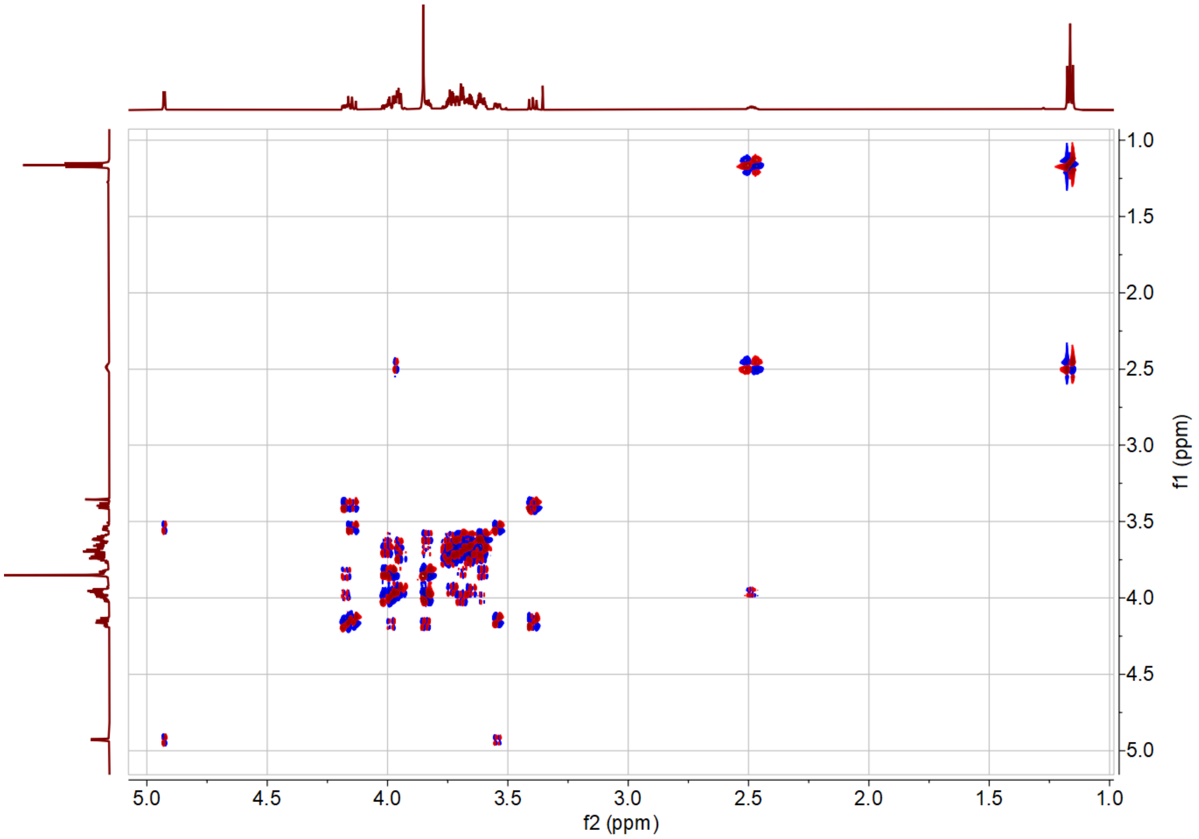

2D NOESY spectrum

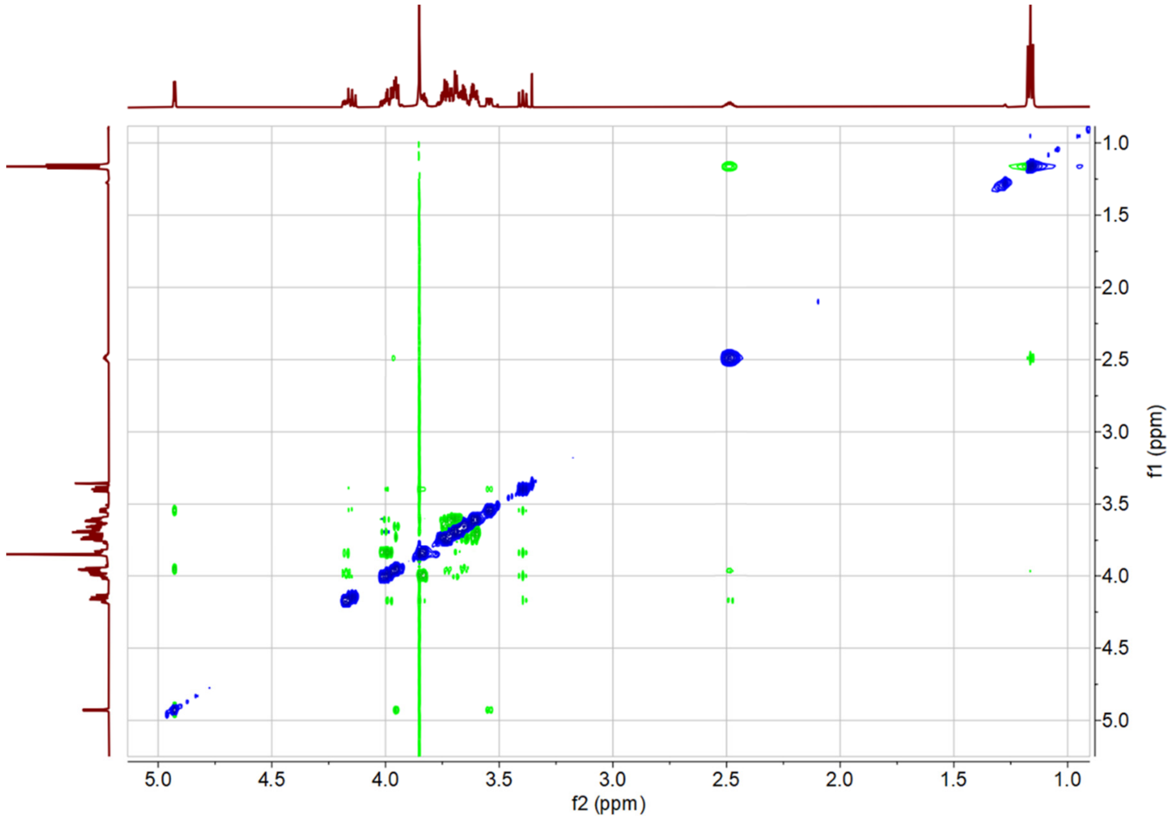

7. HR MS spectrum of receptor 1.

Acquisition Parameter

Method:	ETH_HyStar_HPLC_QTOF_POS_LowMass_Loop-AS.m			Acquisition Date:	08.04.2021 12:25:55
File Name:	D:\Data\bmax0115xx\BMAX011597.d			Operator:	Daniel Wirz
Source Type	ESI	Ion Polarity	Positive	Set Nebulizer	1.6 Bar
Focus	Not active	Set Capillary	4500 V	Set Dry Heater	200 °C
Scan Begin	50 m/z	Set End Plate Offset	-500 V	Set Dry Gas	8.0 l/min
Scan End	1300 m/z	Set Collision Cell RF	200.0 Vpp	Set Divert Valve	Source

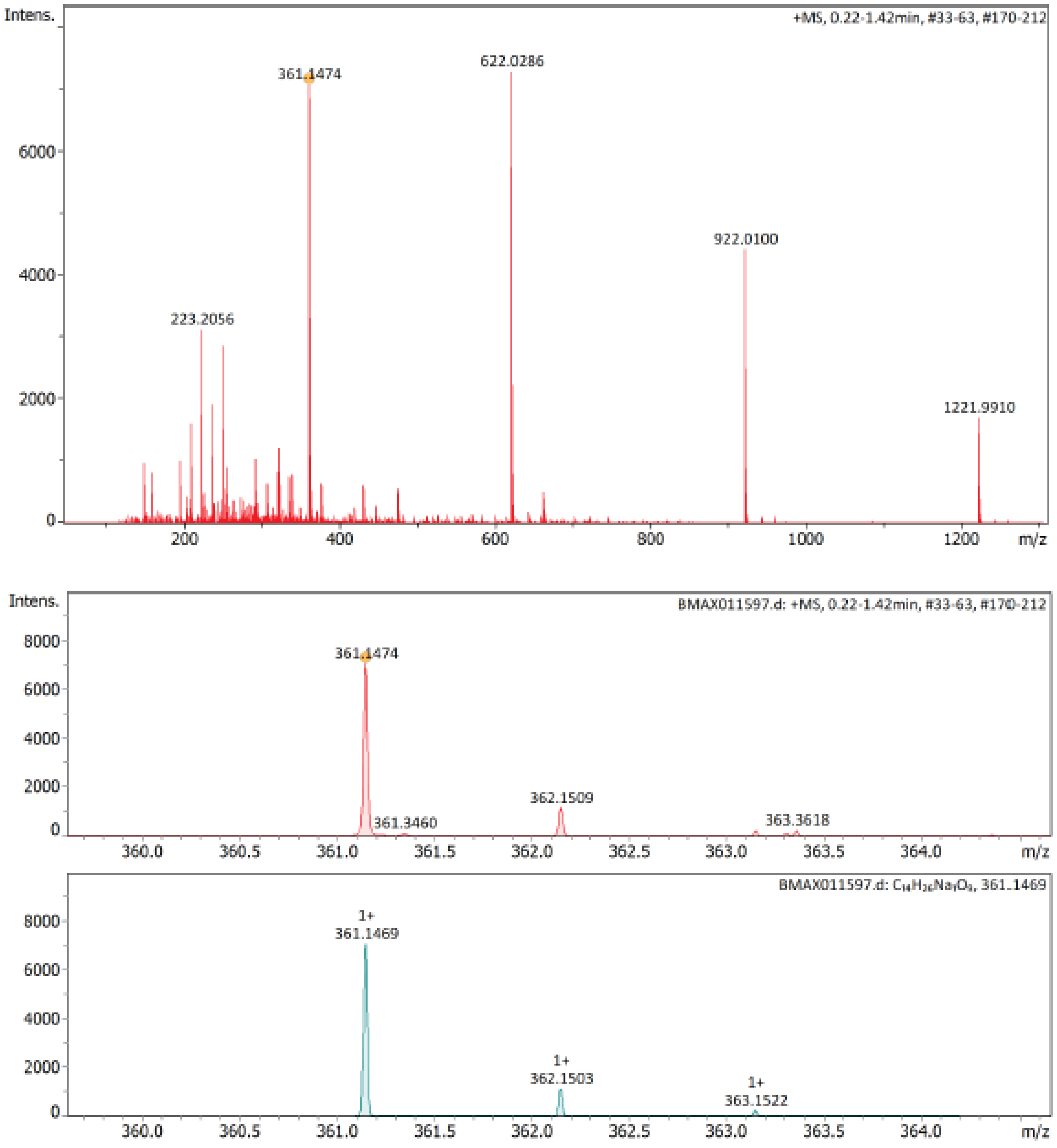

Evaluation Spectra / Validation Formula:

#	Ion Formula	Adduct	m/z	z	Meas. m/z	mSigma	N-Rule	err [mDa]	err [ppm]
1	C14H26NaO9	M+Na	361.1469	1+	361.1474	1.7	ok	-0.5	-1.3
